# Heterogeneity within the Oregon Health Insurance Experiment: An application of causal forests

**DOI:** 10.1371/journal.pone.0297205

**Published:** 2024-01-18

**Authors:** Zaid Hattab, Edel Doherty, Andrew M. Ryan, Stephen O’Neill

**Affiliations:** 1 J.E. Cairnes School of Business and Economics, University of Galway, Galway, Ireland; 2 Department of Mathematics, An-Najah National University, Nablus, State of Palestine; 3 Department of Health Services, Policy, and Practice, Brown University, Providence, Rhode Island, United States of America; 4 Department of Health Services Research and Policy, London School of Hygiene and Tropical Medicine, London, United Kingdom; Cairo University, EGYPT

## Abstract

Existing evidence regarding the effects of Medicaid expansion, largely focused on aggregate effects, suggests health insurance impacts some health, healthcare utilization, and financial hardship outcomes. In this study we apply causal forest and instrumental forest methods to data from the Oregon Health Insurance Experiment (OHIE), to explore heterogeneity in the uptake of health insurance, and in the effects of (a) lottery selection and (b) health insurance on a range of health-related outcomes. The findings of this study suggest that the impact of winning the lottery on the health insurance uptake varies among different subgroups based on age and race. In addition, the results generally coincide with findings in the literature regarding the overall effects: lottery selection (and insurance) reduces out-of-pocket spending, increases physician visits and drug prescriptions, with little (short-term) impact on the number of emergency department visits and hospital admissions. Despite this, we detect quite weak evidence of heterogeneity in the effects of the lottery and of health insurance across the outcomes considered.

## Section 1: Introduction

Access to healthcare is one of the main components of the Human Development Index (HDI) [1]. Yet, even in developed countries, such as the United States (US), significant barriers to healthcare remain. Cost is one such barrier, leading to underutilization and potentially a reduction in population health, affecting millions of families in the US and beyond [2, 3]. Health Insurance (HI) is one potential mechanism by which this barrier can be addressed [4, 5]. Notwithstanding this, in the US, 29.6 million people among the nonelderly population remained uninsured in 2019 [6]. This is despite the implementation of policies designed to expand coverage such as the Affordable Care Act (ACA) in 2010, which represented the largest expansion in coverage since the establishment of Medicaid and Medicare in 1965, and other significant changes may also occur following the recent passage of the Inflation Reduction Act in 2022 which will allow Medicare to negotiate prescription drug prices. However, HI policy in the US remains controversial and has undergone a number of modifications since its inception. This is unsurprising given the questions about the economic efficiency of health insurance [7], and concerns such as health-based insurance provision and pricing, and adverse selection [8].

HI has been found to have desirable, albeit modest, impacts on health, healthcare utilization, and quality of life outcomes in studies using observational data [9–12]. After the implementation of the ACA, multiple studies found that the program and its expansion substantially improved young adults’ perceptions of their overall physical, and mental health [13–15], and so it led to a general improvements in their self-reported health [16, 17]. Other work has shown that Medicaid expansion following the ACA reduced mortality [18], resulting in approximately 19,000 few deaths in the 4 years following implementation [19]. Experimental evidence from an IRS outreach campaign also found that coverage expansion following the ACA reduced mortality [20].

One important weakness of the literature in this area is that it has largely relied on observational data, creating difficulties for identifying causal impacts of HI on health and other outcomes. A well-established issue is that there may be significant observed and unobserved differences in individuals’ baseline characteristics between the insured and uninsured groups. Such differences due to self-selection into HI can lead to biased estimates of the effect of health insurance if unaccounted for [21]. Studies that considered randomized assignment of HI such as the RAND Health Insurance Experiment (HIE), conducted from 1971 to 1986, and the Oregon Health Insurance Experiment (OHIE), conducted in 2008, allow a more robust analysis of the effect of expanding public health insurance. In this paper, we focus on the OHIE which is a randomized controlled study where individuals who signed up to a waiting list were given the opportunity to apply for Medicaid coverage if they were selected randomly by lottery, while those not selected were not offered this opportunity. This randomization allows unobserved confounding which limits observational studies’ ability to credibly identify causal effects to be overcome.

A number of studies have been conducted using data from the OHIE to estimate the effects of expanding coverage on various types of outcomes. Baicker et al. [22], which we refer to here as ’the original study’, report that Medicaid coverage did not significantly improve measured physical health outcomes, although they found it increased the use of health care services, improved diabetes detection and management, lowered rates of depression, and reduced financial strain. It should be noted that the follow-up period of the study was relatively short (approximately 2 years), and it is possible that clinical effects on physical outcomes take longer than this to manifest [22]. HI was found by Finkelstein et al. [23] to improve self-reported health as measured by the mental and physical component scores obtained using the short form medical outcomes questionnaire (SF-8) [24]. Moreover, Baicker et al. [25] reported that HI reduced the prevalence of undiagnosed depression. HI was found to significantly increase healthcare utilization captured by outcomes such as the number of emergency department visits [26] and number of medications used [27] and to decrease financial hardship outcomes such as the amount of out-of-pocket spending on healthcare [22, 28].

While these studies provide credible evidence of the effects of lottery selection and HI on a range of health, healthcare and other outcomes, beyond exploring effects in a limited set of subgroups separately, they generally do not explore effect modification, whereby the effects of HI differ depending on the individuals’ characteristics. In the clinical and economic evaluation literature, there has been a growing interest in ‘personalized’ medicine, which recognizes the need to evaluate and estimate the heterogeneous effects of health interventions [29]. Similarly, there is increased recognition of the importance of heterogeneity when evaluating the effects of health policies. Determining whether the impacts of health insurance coverage are heterogeneous among different subpopulations could enable HI provision to be targeted to subpopulations who are anticipated to benefit most from it [30].

Surprisingly, little attention has been given to factors influencing the uptake of HI among winners of the OHIE lottery. One exception is Allen et al. [31], who report a descriptive analysis of uptake and self-reported reasons for not applying or the rejection of their application, concluding that the imperfect take-up of Oregon Health Plan Standard (OHP-standard) coverage appeared to be attributable both to ineligibility and to difficulty obtaining coverage for the truly eligible. For instance, the application materials for the OHP-standard coverage sent to winners included a two-page application form which could be accompanied by up to eight supplemental forms, and documentation of identity and citizenship and proof of income had to be returned with the completed form [31]. As noted by Allen et al. [31], exploring take up is interesting in this context as Oregon differs from standard Medicaid programs because the standard Oregon Health Plan (OHP-standard) does not allow “conditional coverage” where individuals sign up for coverage only when they require medical care. Here, individuals selected in the lottery had only forty-five days from that point to apply for insurance [31]. Hence individuals had little opportunity to wait until becoming ill or Pregnant before applying. Such delayed uptake has been identified as a driver of low Medicaid uptake. Therefore, we might expect that the demand for OHP- standard for those eligible to be higher for OHP-standard than for Medicaid more generally.

To explore the effects of lottery selection on HI uptake, we use Causal Forests [32], a non-parametric machine learning approach which builds on the random forest method [33] and on causal trees [34]. CF has been shown to perform well in estimating causal effects in Monte Carlo simulations [35] and to provide credible estimates in real case studies [36–38]. The analysis shows that winning the lottery has a differential impact on enrollment rates into HI according to individual characteristics. Specifically, winning the lottery increases the probability of uptake of health insurance significantly more for whites and/or those aged 49–64 than those aged 19–34 and/or non-white races (including black, Hispanic, and other races), which has not previously been shown within the literature on HI selection. To aid understanding of these results we further explore heterogeneity in the likelihood of submitting an application to obtain HI and on the probability that this application will be approved. We find that heterogeneity arises at different stages of the uptake process for different groups, firstly in terms of the decision to apply, and then in approval rates and finally in uptake rates.

Next we turn to the effects of lottery selection and HI on outcomes. While most of the previous studies have focused on overall aggregate effects of HI (or only include limited pre-specified subgroup analyses ([39, 40]), a recent literature has begun to explore heterogeneity in the effects of HI using data from the OHIE [41–45]. However, these studies have focused on a narrower set of subgroups and outcomes, primarily examining the effects of HI. For instance, Denteh & Liebert [44] applied the causal forest approach to the OHIE data using a one year time horizon, concentrating solely on the HI effects on the emergency department visits outcome. Similarly, Johnson et al. [45] investigated the heterogeneous effects of HI on the number of days an individual’s health condition did not impede their routine activities, using IV-matching, where similar pairs of individuals are found but where one unit has not been selected in lottery and the other is selected (i.e. the IV is used in place of treatment in typical matching procedures), and applying the classification and regression trees (CART) to paired difference in outcome to identify subgroups. Closed testing of heterogeneous complier average causal effect is carried out to control for familywise error rate [46]. In addition, Qiu et al. [41] utilizing the same data subset as in our study, employed a two-stage regression LASSO model. This parametric approach incorporates a penalty parameter into the method originally used in the original study [22], and explored the heterogeneity within a limited number of subgroups. Our study explores heterogeneity of effects within the OHIE non-parametrically and at a more granular level. Specifically, we estimate individual level effects allowing effects to be determined by complex interactions of individuals’ characteristics, and then aggregate these for pre-specified (for this analysis) subgroups of interest. We focus on three main types of outcomes that have previously been shown in the literature to be affected by health insurance: self-reported health, health care utilization, and financial strain. In addition to exploring the effects of lottery selection using the causal forest method, we also conduct an Instrumental Variable (IV) [47] analysis to estimate the effects of HI coverage, using lottery selection as an instrument for whether the individual is covered by HI, since an individual randomly selected in the lottery has a higher probability of being enrolled in HI, but have similar characteristics to those not selected given the lottery was random (See supplement for this analysis).

In accordance with findings in the literature, the (short-term) effects of health insurance on the overall sample are found to be fairly modest. We find some evidence of heterogeneity in effect sizes, albeit the study is underpowered to detect effects in subgroups, rendering the evidence of heterogeneous effects of health insurance questionable. We conclude that larger samples (e.g. administrative data) may therefore be required to improve the targeting of HI.

The remainder of this paper is organized as follows. Section 2 presents the data description and Section 3 explains the methods. Section 4 presents our main findings for intent-to-treat analysis while Section 5 presents the discussion and conclusions. Results of the IV analysis of the effects of health insurance and additional analyses are available in the supplementary appendix.

## Section 2: Data description

A total of 89,824 residents of Oregon submitted their names in a lottery held in 2008 to determine eligibility to apply to enroll in the OHP-standard. Our sample contains a subset of 12,229 individuals who satisfied the inclusion criteria for the original study [22] and who responded to the in-person survey by October 2010. This was in contrast to the mail responders, which comprised 23,741 individuals used in Finkelstein et al. [23]. The smaller sample size was chosen because it yielded a higher response rate and allowed for a longer time-horizon analysis. One limitation of this data is the fact that it is restricted to the Portland-metro area for logistical reasons. However, additional analyses have been conducted on the mail-survey-respondent sample for comparison, albeit with a reduced set of variables since baseline comorbidities were not captured in this survey. The results of these analyses can be found in the supplementary appendix. These OHIE data are publicly accessible from the National Bureau of Economic Research (NBER) [48]. In our analysis, we consider eight outcomes that can be classified into three categories: (i) Self-reported health outcomes which include the physical and mental component scores measuring health-related quality of life, obtained using the short form medical outcomes survey of the SF-8 questionnaire; (ii) financial strain which captures the amount of out-of-pocket spending on medical services; and (iii) five health care utilization outcomes, including the numbers of: prescription drugs, office visits, hospital admissions, outpatient surgery visits, and emergency department visits. The heterogeneous effects of lottery selection and enrollment in the standard Oregon health insurance plan are estimated for each outcome for each individual based on 19 baseline covariates. These individual level effects were then aggregated for subgroups defined by age group, gender, race, whether the individual was diagnosed with depression and whether the individual was considered ‘high risk’, defined as having had a diagnosis of diabetes, hypertension, hypercholesterolemia, myocardial infarction, or congestive heart failure before the lottery took place [22]. All analyses in the next sections are weighted using survey weights included in the OHIE dataset to account for the differential probability that OHIE investigators targeted each household for non-response follow-ups [42]. These weights ensure that our sample is representative of the full sampling base [22], since they are based on the likelihood that each individual is included in a given lottery draw, accounting for selection bias between waves [22]. Throughout the analysis we cluster by household.

## Section 3: Methodology: Causal forest (intent-to-treat analysis)

The data are publicly accessible at the National Bureau of Economic Research (NBER) [48] and were anonymized before we accessed them on July 5th, 2021. Hence, we did not have access to any details that could identify individual participants during or after the data collection. Ethical approval was obtained from the Research Ethics Committee at the University of Galway. The original study [22] explicitly confirms the receipt of approvals from multiple institutional review boards and emphasizes the acquisition of written informed consent from all study participants.

For each individual *i* = 1,…,12,229 in the dataset, we observe a binary health insurance indicator *D*_*i*_, a binary indicator of lottery selection Z_i_, a vector of baseline characteristics of individual *i* denoted by ***X***_*i*_ that includes the 19 covariates which are shown in Table 1 and a set of outcomes, *Y*_*ij*_, where *j* indexes the outcomes. The outcomes are listed in Table 2. We consider a generic outcome Y_i_ for the methods’ description. With the exception of uptake of HI, two analyses were conducted for each outcome: (i) an intent-to-treat analysis where the effects of lottery selection (*Z*_*i*_) are estimated using the CF method, and (ii) an IV analysis where the effects of HI (denoted by *D*_*i*_) are estimated using the instrumental forest method described in the supplementary material (See supplement and S1 File) using lottery selection (denoted by *Z*_*i*_) as the IV. While the IV forest method has the advantage that it protects against endogeneity/unobserved confounding and focusses on the effects of HI rather than the lottery, this comes at the cost of a significant loss of efficiency. We describe each method using the Neyman-Rubin potential outcomes framework [49, 50].

**Table 1:** Baseline characteristics’ distribution*.

	Not Selected in Lottery (N = 5842)	Selected in Lottery (N = 6387)	Standardized Difference	Not Enrolled in insurance (N = 10130)	Enrolled in insurance (N = 2099)	Standardized Difference
**Age, %**						
**19–34 years**	36.0%	35.2%	0.028	36.4%	31.4%	0.107
**34–49 years**	36.4%	37.0%	0.006	36.4%	38.1%	0.019
**49–64 years**	27.6%	27.7%	0.023	27.1%	30.5%	0.091
**Sex, %**						
**Female**	56.8%	56.0%	0.011	56.1%	58.2%	0.04
**Male**	43.1%	44.0%	0.011	43.9%	41.8%	0.04
**Race, %**						
**White**	68.9%	68.5%	0.006	67.4%	75.8%	0.204
**Black**	10.5%	10.06%	0.025	9.8%	12.5%	0.066
**Hispanic**	17.2%	18.0%	0.012	19.25%	9.2%	0.299
**Other race**	14.8%	15.0%	0.009	15.2%	13.2%	0.064
**Preferred English material, %**	90.7%	89.4%	0.024	88.7%	97.1%	0.34
**Pre-Lottery diagnosis, %**						
**Asthma**	19.9%	18.8%	0.033	18.9%	21.5%	0.060
**Diabetes**	7.2%	6.9%	0.003	7.2%	6.4%	0.036
**Hypertension**	18.1%	18.0%	0.003	17.9%	18.9%	0.021
**High cholesterol**	12.7%	12.4%	0.003	12.40%	13.2%	0.021
**Heart attack**	2.0%	1.8%	0.006	1.80%	2.5%	0.060
**Congestive heart failure**	1.0%	1.10%	0.012	1.00%	1.2%	0.014
**Emphysema/ COPD**	2.3%	2.2%	0.009	2.00%	3.5%	0.071
**Kidney failure**	1.8%	1.7%	0.013	1.77%	1.6%	0.008
**Cancer**	4.3%	4.3%	0.013	4.10%	5.2%	0.068
**Depression**	35.0%	33.1%	0.039	33.30%	38.5%	0.099
**In high risk subgroup** ^†^ **, %**	27.3%	26.7%	0.003	26.70%	28.5%	0.044
**Household size** ^‡^ **,%**						
**1**	78.8%	71.8%	0.211	74.4%	80.2%	0.108
**2**	21.2%	27.9%	0.207	25.5%	19.6%	0.109
**3**	0.03%	0.23%	0.055	0.12%	0.17%	0.016

*In addition to the covariates that are presented in Table 1, households’ income ranged from (0$-$2,500) to (over $50,000) were also reported before the lottery, however we do not include this covariate in the main analysis as it contains a large number of missing values. We assess the sensitivity of our results to including income categories along with an indicator for missing income, and find results are very similar to those of the main analysis (available from authors on request).

^†^ A high-risk diagnosis was defined as a diagnosis of diabetes, hypertension, hypercholesterolemia, myocardial infarction, or congestive heart failure before the lottery (i.e., before March 2008) [22].

^‡^ The household size is defined as the number of people in the household who are on the lottery waiting list.

**Table 2:** Average (intent-to-treat) treatment effects of OHIE lottery selection using causal forest compared to findings of original study.

Effect of Lottery Selection (95% CI)									
Outcome	Uptake of health insurance (%)	Mental component score	Physical component score	Amount of out-of-pocket spending ($)	Number of prescription drugs	Number of office visits	Number of hospital admissions	Number of out-patient surgery visits	Number of emergency department visits
**Original study ATE**	26.49 (25.1, 27.9)	0.44 (-0.02, 0.90)	0.3 (-0.11, 0.70)	-53 (-101, -6)	0.15 (0.047, 0.26)	0.69 (0.25, 1.13)	0.02 (-0.004, 0.044)	0.008 (-0.006, 0.02)	0.031 (-0.05, 0.11)
**CF Analysis ATE**	26.84 (25.57, 28.12)	0.48 (0.11, 0.86)	0.32 (-0.03, 0.66)	-66 (-109, -24)	0.18 (0.10, 0.27)	0.57 (0.16, 0.99)	0.011 (-0.012, 0.033)	0.004 (-0.010, 0.017)	0.008 (-0.060, 0.076)

In the main analysis, lottery selection is considered as the treatment variable, such that the people who are not selected in the lottery (*Z*_*i*_ = 0) make up the control group while those who are selected (*Z*_*i*_ = 1) constitute the treated group. Here the effect of interest is the effect of the opportunity to apply for HI rather than of HI itself. Theoretically, two potential outcomes are possible: *Y*_*i*_(0) if individual *i* were not selected in the lottery, and *Y*_*i*_(1) if he/she were selected. However, the fundamental problem of causal inference [51] arises since only one of the two potential outcomes is ever observed for an individual. The observed outcome *Y*_*i*_ can be represented as *Y*_*i*_ = *Z*_*i*_×*Y*_*i*_(1) + (1−*Z*_*i*_)×*Y*_*i*_(0), and the individual effect of the lottery on the outcome will be *τ*_*i*_ = Y_i_(1)−Y_i_(0).

In this study, three parameters of interest are obtained by aggregating the τ_i_′*s*: the Average Treatment Effect (ATE) capturing the overall effect on the population, the Conditional Average Treatment Effects (CATE) capturing the individualized average lottery effect for an individual taking account of their baseline characteristics and finally the subgroups’ Average Treatment Effects (GATE) capturing the effect for prespecified subgroups of interest, defined respectively as:

$${ATE}_{Lottery}=E(\tau_{i})$$ (1)

$$CATE(x)=\tau_{i}(x)=E(\tau_{i}|X_{i}=x)$$ (2)

$${GATE}_{Lottery}=E(\tau_{i}|G_{i}=g)$$ (3)

where *g* is one of the pre-specified subgroups defined by the covariates.

When incorporating the covariates X into the model, a reformulation of the observed outcome *Y*_*i*_ can be articulated as follows [52]:

$$Y_{i}=\mu_{i}(X)+Z_{i}\times \tau_{i}(X)+error$$ (4)

where *μ*_*i*_(*X*) represents the prognostic effect of the baseline covariates **X** (or a subset of them), while the moderators of the impact of *Z* are captured by τ_i_(*X*). The conditional mean of *Y* can be represented as [52]:

$$E(Y_{i}|X_{i}=x)=\mu_{i}(x)+e_{i}(x)\times \tau_{i}(x)≔m_{i}(x)$$ (5)

where e_i_(*x*) is the propensity score that is estimated by regressing *Z* on the covariates, and *m*_*i*_(*x*) is referred to as the marginal mean.

To estimate the overall, subgroups’, and individualized effects of lottery selection, we apply the CF method [53], which is a generalization of the random forest of Breiman [33]. Athey & Imbens [34] modified the classification and regression tree (CART) approach to construct a ‘causal tree’ which focuses on estimating conditional treatment effects, *τ*_*i*_(*x*), rather than predicting the outcome (*Y*_*i*_), as is done in a traditional CART. To achieve this, Eq (5) is rewritten as [54]:

$$(Y_{i}|X_{i}=x)=m_{i}(x)+\tau_{i}(X)(Z_{i}-e_{i}(x))+error$$ (6)

This representation enables the estimation of the treatment effects τ_i_(*x*) through a two-step process initiated by regressing the outcome and lottery selection on the covariates to obtain estimates of the marginal mean $\hat{m_{i}}(x)$ and the propensity $\hat{e_{i}}(x)$, respectively. Regression forests are used for this step. Subsequently, the estimands of interest, ${\hat{\tau }}_{i}(x)$, are estimated by finding τ_i_(*X*) which minimizes the locally centered loss function [52]:

$$Loss=\frac{1}{2}{\left[Y_{i}-\hat{m_{i}}(x)-\tau_{i}(x)(Z_{i}-\hat{e_{i}}(x)))\right]}^{2}$$ (7)

This local centering enhances the model’s robustness to potential confounding effects [55].

Further, Athey & Imbens [34] propose an ‘honest’ estimation where the split points for the trees and the effects are estimated on distinct subsamples to prevent overfitting and provide correct inference. A causal tree is obtained by changing the splitting criterion from minimizing the sum of squared errors for the predicted outcome in CART to minimizing the expected mean squared error (EMSE) of the treatment effects, defined as [34]:

$$-\hat{{EMSE}_{\tau }}(S^{tr},N^{est}.T)=\frac{1}{N^{tr}}\sum_{i\in S^{tr}}{\hat{\tau }}^{2}(X_{i}|S^{tr}),T)-(\frac{1}{N^{tr}}+\frac{1}{N^{est}})\sum_{L\in T}\left(\frac{S_{S_{treated}^{tr}(L)}^{2}}{p}+\frac{S_{S_{control}^{tr}(L)}^{2}}{1-p}\right)$$ (8)

where, *S*^*tr*^ is the training subsample that is used to construct the tree *T*, *S*^*est*^ is the estimation subsample which is different from the training subsample, *N*^*est*^ is the number of individuals in the estimation sample, *N*^*tr*^ is the number of individuals in the training subsample, *L* is a ‘leaf’ (i.e. a subgroup defined by the splits) in tree *T*, $S_{S_{treated}^{tr}(l)}^{2}\;and\;S_{S_{control}^{tr}(l)}^{2}$ are the within-leaf variances of outcomes for treated individuals and control individuals respectively, and *p* is the share of treated units.

This splitting criterion prefers leaves (subgroups) with heterogeneous effects by maximizing the first term of (8) which represents the variance of the estimated treatment effect across leaves, and leaves with a good fit by minimizing the second term capturing within-leaf variance. However, an individual tree can be noisy. To overcome this, Wager & Athey [32] proposed the CF which generates an ensemble of *B* causal trees, each of which produces an estimate ${\hat{\tau }}_{b}(X)$, which are then aggregated to obtain a CATE estimate, $\hat{\tau }(X)$. The ${\hat{\tau }}_{b}(X)\prime s$ are estimated using an adaptive locally weighted estimator [54] such that:

$${\hat{\tau }}_{i}(x)=\frac{\sum_{i=1}^{n}\alpha_{i}(x)(Y_{i}-{\hat{\mu }}^{\left(-i\right)}(X_{i}))(Z_{i}-{\hat{e}}^{\left(-i\right)}(X_{i}))}{\sum_{i=1}^{n}\alpha_{i}(x){\left(Z_{i}-{\hat{e}}^{\left(-i\right)}(X_{i})\right)}^{2}}$$ (9)

where the superscript (−*i*) denotes the out-of-bag predictions which are obtained from the subsample of trees where observation *i* was not used to determine the splits, $\hat{\mu }(x)$ is the estimated conditional mean outcome $E[Y_{i}|X_{i}=x]$ obtained by fitting a regression forest, $\hat{e}(x)$ is the estimated conditional propensity score ℙ[*Z*_*i*_ = 1|*X*_*i*_ = *x*] obtained by fitting another regression forest, and *α*_*i*_(*x*) is the weight given to observation *i* which measures how often observation *i* is assigned to the same leaf that the point (*x*) lies within [54]. This method is implemented in the generalized random forest R package *grf* [47]. We estimate GATEs for our pre-specified subgroups by taking the estimated individualized treatment effects and plugging them into an AIPW estimator [56] of group average treatment effects [57]. The strength of the AIPW estimator [55] stems from its double robustness property which means that the estimates of the average treatment effects of the population and the subgroups remain consistent even if one of the propensity or outcome regression forests is mis-specified [58].

In our study, the AIPW scores that are averaged to obtain the ATE and GATE estimates are obtained using the following formula [59]:

$${\hat{\gamma }}_{i}=\hat{m_{i}}(X_{i},1)-\hat{m_{i}}(X_{i},0)+\frac{\left(Y_{i}-\hat{m_{i}}(X_{i},Z_{i}))(Z_{i}-\hat{e}(X_{i})\right)}{\hat{e}(X_{i})(1-\hat{e}(X_{i}))}$$ (10)

where $\hat{m_{i}}(x,z)=E[Y_{i}(z)|X_{i}=x]$ denote the nonparametric estimate of the conditional mean of the treatment group.

We implement the CF for each outcome using 20,000 trees, while all other tuning hyperparameters (sample fraction used to build each tree, number of variables tried for each split, minimum number of individuals in each tree leaf, honesty fraction, and parameters which determine the imbalance of the splits) are determined using cross -validation using 1,000 forests to fit the tuning model, with a minimum of 500 trees in each forest. The number of random parameter values considered when using the model to select the optimal parameters is 5,000.

The forests were fitted in two stages. During the first stage, the model is fitted over all covariates. The second stage considers only the most important covariates, i.e. those whose importance exceeds 20% of the average importance (see S3 and S4 Tables in the appendix), where importance is defined as the simple weighted sum of how many times each covariate was split at each depth in the forest [54]. To test for heterogeneity, omnibus heterogeneity tests were performed, and their results are presented in the supplement material (See supplement, S3 and S4 Files).

## Section 4: Results: Effects of lottery selection–causal forest analysis

Baseline characteristics of the individuals are displayed in Table 1. Standardised differences are used to check for meaningful differences between the control and treatment groups in terms of the means of their covariates, first using lottery selection as the treatment and then separately using Medicaid coverage as the treatment instead. Characteristics are generally well balanced for the lottery selection (column 4) as one might expect given the randomization, although there is some evidence of imbalance for household size, preferring English materials and depression albeit differences are relatively small in magnitude. In contrast, meaningful differences are observed between the insured and the non-insured individuals across most characteristics (column 7), particularly in age, race and depression status. These findings confirm the importance of instrumenting by the lottery to mitigate possible unobserved confounding when assessing the effects of health insurance (See supplement and S2 File).

**Fig 1:**
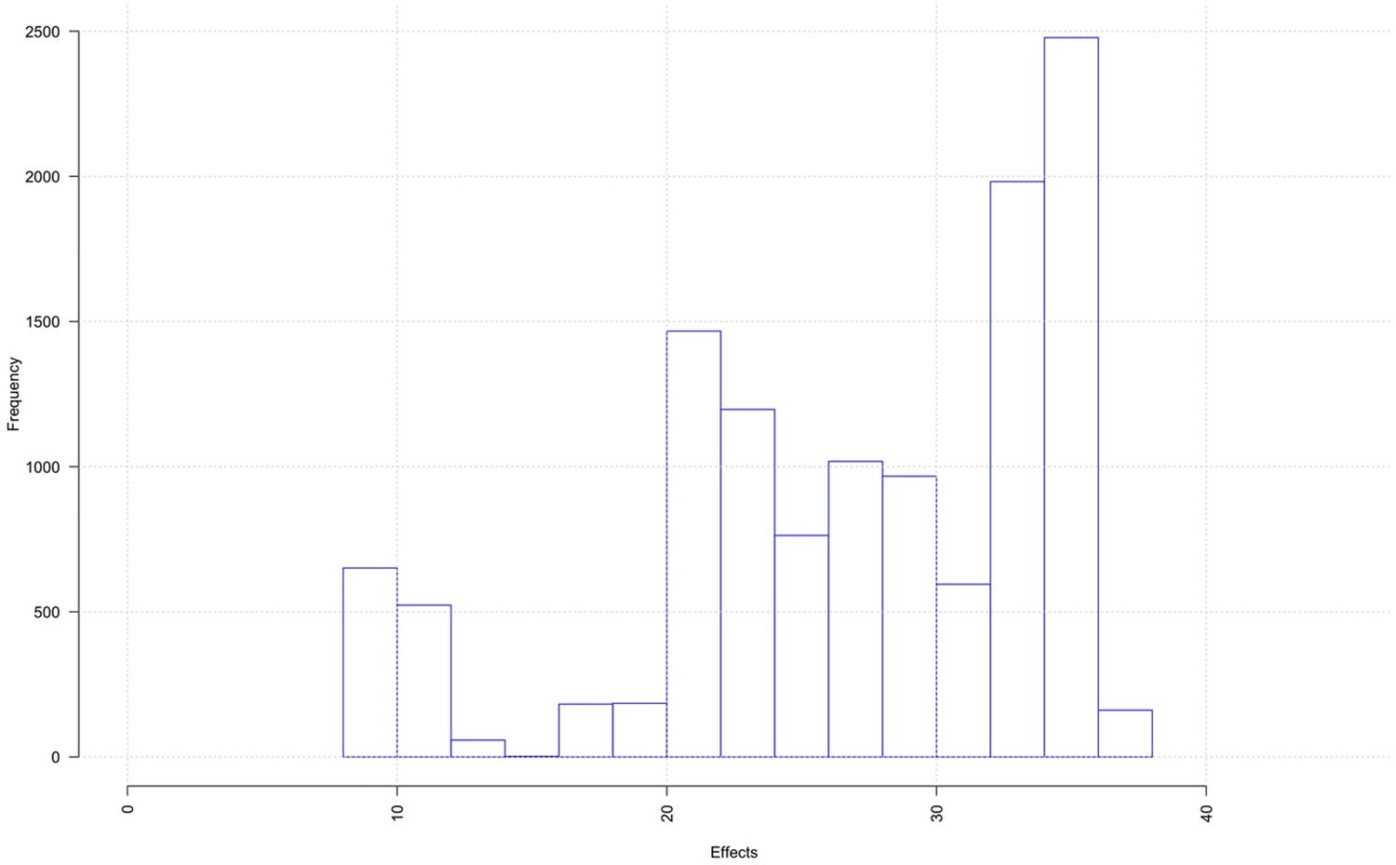
Individualized estimated treatment effects of lottery selection on the probability of OHP-standard uptake. A histogram that shows the distribution of the estimated individualized effects.

**Fig 2:**
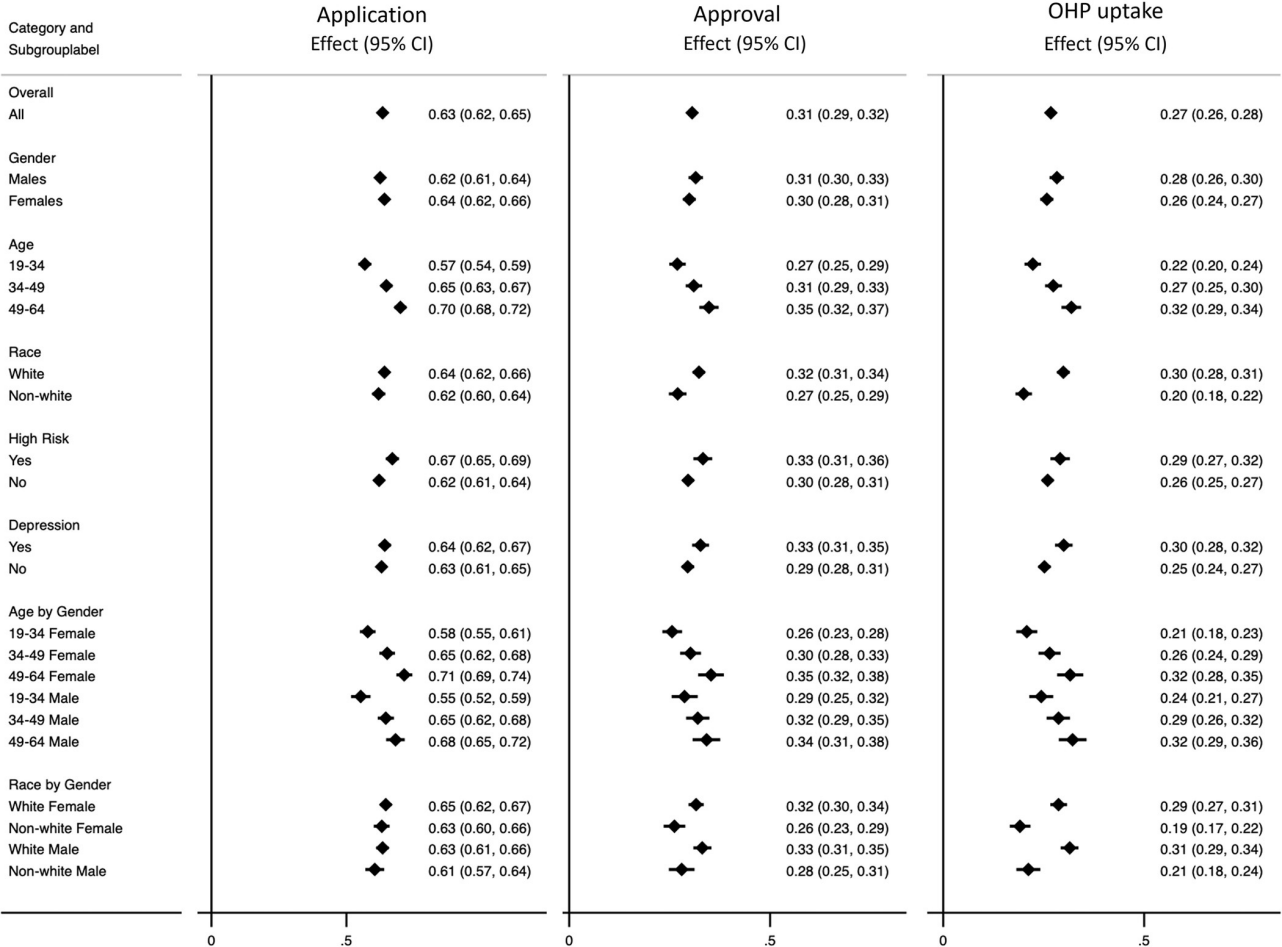
Forest plot for subgroups’ conditional average treatment effects of lottery selection on submitting (left panel) and approval (middle panel) of OHP application, and uptake of OHP (Right panel). This plot shows the point estimates of the GATE effects with a 95% confidence intervals.

While our primary interest is in subgroup effects, the intent-to-treat analysis considering the overall ATE of lottery selection for each outcome using the CF method are shown in Table 2 for validation purposes. The table also reports the findings from the original study [22] for comparison. In general, the CF estimates are similar to the original study’s estimates, although we detect a significant overall effect for the mental health component score (0.48, 95% CI: 0.11 to 0.86) unlike the original study albeit the point estimate is very similar (0.44, 95% CI: -0.02 to 0.90). An important finding of the intent-to-treat analysis is that while being selected in the lottery increases the probability of enrollment in the Medicaid coverage program by more than 26 percentage points on average, there is considerable heterogeneity in the effects of the lottery across individuals and subgroups. Fig 1 shows the distribution of the estimated effects of the lottery on the probability of enrolling in Medicaid for each individual, with effects ranging from less than 10 to approximately 40 percentage points. This is supported by the GATEs for each prespecified subgroup which are reported in Table 3 and displayed in Fig 2 (Right panel). Several confidence intervals of the GATEs do not include the average overall effect (26.84%) indicating heterogeneity in enrollment. The average effect for the subgroup of whites (29.9%, CI: 28.3% to 31.5%) is significantly higher than the effect for the subgroup of non-white races (19.9%, CI: 18.0% to 22.1%). Similarly, effects for the subgroups of individuals aged 49–64 years old, white males, are significantly higher than the overall effect, while effects for non-whites and individuals aged 19–34 years are significantly lower than the overall effect. Thus, there appears to be some self-selection into HI, although some of these differences may be attributable to different success rates for applicants’ post-lottery selection, since lottery selection conferred the right to apply for Medicaid but did not guarantee enrollment for applicants. Notably, of the 6,837 individuals selected in the lottery in our sample of 12,229 individuals, only 4,095 (64.1%) subsequently applied for health insurance, with 2,282 (35.7%) opting not to apply for a variety of reasons. Of those that did apply, 2,138 (52.2%) had their application rejected and 1,957 (47.8%) had their application approved.

**Table 3:** Subgroups’ effects of lottery selection (intent-to treat) based on causal forest.

Effect of Lottery Selection (95% CI)									
Outcome Subgroup	Uptake of health insurance (%)	Mental component score	Physical component score	Amount of out-of-pocket Spending ($)	Number of prescription drugs	Number of office visits	Number of hospital admissions	Number of out-patient surgery visits	Number of emergency department visits
**Overall**	26.84 (25.57, 28.12)	0.48 (0.11, 0.86)	0.32 (-0.03, 0.66)	-66 (-109, -24)	0.18 (0.10, 0.27)	0.57 (0.16, 0.99)	0.011 (-0.012, 0.033)	0.004 (-0.010, 0.017)	0.008 (-0.060, 0.076)
**Males**	28.25 (26.46, 30.0)	0.36 (-0.18, 0.90)	0.14 (-0.38, 0.67)	-59 (-118, -0.5)	0.16 (0.03, 0.28)	0.37 (-0.18, 0.91)	0.020 (-0.013, 0.054)	0.008 (-0.012, 0.027)	0.045 (-0.052, 0.142)
**Females**	25.76 (24.11, 27.41)	0.58 (0.07, 1.01)	0.45 (-0.005, 0.90)	-72 (-131, -12)	0.20 (0.09, 0.32)	0.73 (0.13, 1.33)	0.003 (-0.028, 0.039)	0.001 (-0.018, 0.020)	-0.020 (-0.115, 0.073)
**19–34 years**	22.27 (20.22, 24.32)	0.33 (-0.27, 0.95)	0.1 (-0.42, 0.62)	-127 (-191, -63)	0.14 (0.05, 0.24)	-0.16 (-0.84, 0.52)	-0.0008 (-0.035, 0.034)	-0.0196 (-0.04, 0.001)	-0.063 (-0.188, 0.063)
**34–49 years**	27.44 (25.34, 29.54)	0.49 (-0.13, 1.11)	0.17 (-0.41, 0.76)	-9 (-81, 64)	0.22 (0.08, 0.37)	1.37 (0.65, 2.1)	0.011 (-0.027, 0.049)	0.019 (-0.003, 0.041)	0.042 (-0.071, 0.155)
**49–64 years**	31.88 (29.44, 34.32)	0.66 (-0.06, 1.39)	0.79 (0.07, 1.50)	-67 (-154, 20)	0.18 (-0.02, 0.39)	0.44 (-0.32, 1.19)	0.025 (-0.022, 0.072)	0.013 (-0.015, 0.041)	0.052 (-0.058, 0.162)
**White**	29.91 (28.32, 31.50)	0.40 (-0.06, 0.86)	0.41 (-0.02, 0.84)	-71 (-126, -16)	0.21 (0.10, 0.32)	0.78 (0.24, 1.31)	0.013 (-0.015, 0.041)	0.010 (-0.007, 0.027)	0.012 (-0.073, 0.096)
**Non-white**	20.04 (17.98, 22.08)	0.67 (0.02, 1.32)	0.11 (-0.46, 0.68)	-57 (-120, 7)	0.12 (-0.01, 0.26)	0.12 (-0.51, 0.75)	0.0054 (-0.032,0.043)	-0.010 (-0.032, 0.012)	-0.001 (-0.114, 0.112)
**High risk**	29.10 (26.69, 31.50)	-0.04 (-0.79, 0.70)	0.54 (-0.16, 1.25)	-79 (-178, 20)	0.20 (-0.04, 0.43)	0.41 (-0.49, 1.31)	0.020 (-0.036, 0.076)	0.011 (-0.020, 0.042)	0.076 (-0.065, 0.217)
**Non-high risk**	26.0 (24.53, 27.47)	0.68 (0.25, 1.11)	0.23 (-0.16, 0.63)	-62 (-79, 16)	0.18 (0.10, 0.26)	0.63 (0.17, 1.10)	0.007 (-0.016, 0.030)	0.001 (-0.013, 0.016)	-0.017 (-0.094, 0.060)
**Depressed**	30.01 (27.82, 32.20)	0.27 (-0.43, 0.96)	0.08 (-0.55, 0.71)	-77 (-161, 8)	0.21 (0.02, 0.39)	0.72 (-0.18, 1.62)	0.041 (-0.007, 0.088)	0.009 (-0.019, 0.037)	0.006 (-0.144, 0.146)
**Non-depressed**	25.21 (23.67, 26.74)	0.59 (0.15, 1.03)	0.44 (0.03, 0.85)	-61 (-109, -13)	0.17 (0.09, 0.26)	0.50 (0.07, 0.92)	-0.005 (-0.029, 0.019)	0.001 (-0.013, 0.016)	0.011 (-0.06, 0.082)
**19–34 years females**	20.80 (18.18, 23.43)	0.60 (-0.21, 1.41)	0.13 (-0.55, 0.81)	-144 (-232, -57)	0.15 (0.02, 0.28)	-0.37 (-1.36, 0.62)	-0.002 (-0.051,0.046)	-0.022 (-0.050, 0.007)	-0.094 (-0.268, 0.080)
**34–49 years females**	26.46 (23.71, 29.22)	0.87 (0.01, 1.73)	0.57 (-0.21, 1.4)	-0.5 (-108, 107)	0.27 (0.06, 0.47)	2.06 (0.99, 3.12)	-0.013 (-0.066, 0.040)	0.023 (-0.009, 0.054)	0.011 (-0.144, 0.167)
**49–64 years females**	31.61 (28.35, 34.87)	0.15 (-0.85, 1.14)	0.72 (-0.21, 1.66)	-68 (-189, 54)	0.20 (-0.08, 0.49)	0.44 (-0.61, 1.49)	0.032 (-0.026, 0.091)	0.004 (-0.034, 0.043)	0.035 (-0.112, 0.183)
**19–34 years males**	24.40 (21.42, 27.38)	-0.05 (-0.96, 0.86)	0.06 (-0.75, 0.86)	-101 (-194, -10)	0.14 (0.01, 0.27)	0.13 (-0.71, 0.96)	0.0017 (-0.045, 0.048)	-0.016 (-0.045, 0.014)	-0.017 (-0.191, 0.157)
**34–49 years males**	28.65 (25.74, 31.56)	0.026 (-0.85, 0.90)	-0.32 (-1.17, 0.53)	-19 (-112, 75)	0.17 (-0.03, 0.36)	0.53 (-0.39, 1.44)	0.041 (-0.013, 0.094)	0.015 (-0.014, 0.045)	0.079 (-0.082, 0.240)
**49–64 years males**	32.21 (28.75, 35.67)	1.30 (0.26, 2.34)	0.87 (-0.21, 1.95)	-66 (-190, 59)	0.16 (-0.14, 0.45)	0.43 (-0.66, 1.53)	0.015 (-0.060, 0.09)	0.024 (-0.017, 0.07)	0.073 (-0.091, 0.237)
**White females**	28.73 (26.65, 30.81)	0.51 (-0.11, 1.14)	0.64 (0.07, 1.20)	-82 (-160, -5)	0.20 (0.05, 0.35)	0.85 (0.08, 1.62)	-0.0015 (-0.038, 0.035)	0.006 (-0.018, 0.029)	-0.060 (-0.179, 0.059)
**Non-white females**	19.13 (16.57, 21.70)	0.71 (-0.16, 1.59)	0.04 (-0.72, 0.80)	-48 (-136, 40)	0.22 (0.04, 0.41)	0.45 (-0.46, 1.36)	0.014 (-0.042, 0.069)	-0.009 (-0.038, 0.021)	0.066 (-0.083, 0.215)
**White males**	31.45 (29.24, 33.66)	0.25 (-0.41, 0.91)	0.12 (-0.54, 0.77)	-55 (-131, 20)	0.23 (0.08, 0.38)	0.67 (-0.02, 1.37)	0.032 (-0.011, 0.075)	0.017 (-0.007, 0.040)	0.106 (-0.011, 0.223)
**Non-white males**	21.19 (18.21, 24.18)	0.61 (-0.32, 1.54)	0.20 (-0.65, 1.06)	-68 (-158, 21)	-0.007 (-0.20, 0.18)	-0.31 (-1.14, 0.53)	-0.005 (-0.054, 0.044)	-0.012 (-0.046, 0.021)	-0.089 (-0.26, 0.083)

To assess whether the heterogeneity in uptake arose from differences in application or approval rates, we estimated two further Causal Forests to aid our understanding of heterogeneity in uptake. First, in *CF_applied* (Fig 2, Left panel), the dependent variable equals one if the individual applied for HI (regardless of whether their application was accepted/rejected) and 0 otherwise. This allows us to explore whether the heterogeneity in uptake is driven by differences in decisions whether or not to apply after HI lottery selection. Secondly, in *CF_approved* (Fig 2, Middle panel), the dependent variable equals one if the individual’s application was approved for HI and 0 otherwise. For the sample as a whole, the reduction in the effects of lottery selection between stages (application, approval or uptake) is 33 percentage points from application to approval and 4 percentage points from approval to uptake. Fig 2 shows that heterogeneity is present among age-by-gender and race subgroups, with the pattern of heterogeneity differing somewhat by stage. For instance, despite the fact non-white races are only 2 percentage points (64% vs. 62%) less likely than whites to apply, they were 10 percentage points less likely to uptake HI (30% vs. 20%).

To explore the reasons for individual’s failure to apply or for their application to be rejected, we rely on additional data for a subset of individuals in our sample, which comes from an initial mail survey conducted by the OHIE study team from June-November 2008 [23]. This survey involved 58,405 individuals, of which 26,423 responses were received. The respondents were limited to those meeting specific criteria: they had to be born between 1944 and 1989, have an Oregon address, and not be associated with an institutional address or signed up by an unrelated third party (e.g., a company) [31]. Of the 12,229 individuals included in our analysis, 5,959 responded to this initial mail survey. It is important to note that this subset is not representative of the full sample, so caution is required in drawing conclusions more broadly from the following findings. Of the 5,959 respondents, 877 did not receive an OHP application form as of the date of survey response, and 2,290 did not win the lottery so were not eligible to apply. For 126 respondents it was unknown whether they received an application form. Of the remainder, 2,223 respondents had won the lottery and received an OHP application form, while 443 received an application without winning the lottery. Approximately 2 percent of the controls (people who did not win the lottery) gained access to OHP Standard through some alternative mechanisms-for instance, pregnant women on OHP Plus can sometimes remain on OHP Standard after giving birth. There is also the possibility that some participants were placed on OHP Plus rather than Standard, since case workers are instructed to check the eligibility of applicants for Plus before placing them on Standard [22]. S5 Table (see supplement and S2 File) reports the reasons given by these latter two subsamples for not applying for OHP-standard or for rejection of their application and presents these by race. The most commonly reported reason for denial was income or assets being too high (16.6%; 80.2% are white and 19.8% are non-white); while the most common reason not to apply related to not finishing the application (40.4%; 72.8% are white and 27.2% are non-white).

Returning to the effects of lottery selection on outcomes, Table 3 displays the estimated average treatment effects for each of the pre-specified subgroups for each of the eight outcomes and the corresponding figures for these are reported in the appendix (S2–S9 Figs, left panels). For all outcomes, there is limited evidence of heterogeneity in magnitude for the subgroup effects, although there is some heterogeneity in the statistical significance of the effects across groups (in the absence of corrections for multiple testing). For instance, most of the subgroups’ effects on the mental health component score are not significant despite the fact that the overall effect is significant (48%, CI: 11% to 86%), reflecting the smaller sample size available for subgroup analyses. Conversely, although the overall effect on the physical health component score is insignificant (32%, CI: -3% to 66%), the effects for 49–64 years, non-depressed, and white-females subgroups are statistically significant. Overall, heterogeneity is quite low for all of the outcomes considered, with the point estimates for the subgroups with the largest and the smallest estimated effects differing by less than ¼ of a standard deviation, where the standard deviation is calculated for the group not selected in the lottery.

S2 Table in the appendix and the right-hand side panels of S2–S9 Figs, report the results of the IV forest analysis, presenting the effects of health insurance, as opposed to the effects of lottery selection. Again, heterogeneity is fairly modest, with the point estimates for the subgroups with the largest and the smallest estimated effects differing by less than 1 standard deviation calculated for the group not selected in the lottery. However, the confidence intervals for the subgroup effects tend to be very wide so we do not discuss these results further here.

To test the robustness of our findings, we carried out two additional analyses and reported these in the supplementary appendix. Firstly, we apply the methods on the larger mail-survey dataset (see supplement and S5 File) as used in Finkelstein et al. [23] consisting of 23,741 observations, and obtain similar results to those using the 12,229-sample (S13 and S14 Figs). Secondly, we have studied the distribution of the baseline characteristics among the lottery draws, and found little evidence of a censoring effect (see supplement, S6 File, S15 and S16 Figs).

## Section 5: Discussion

Existing evidence suggests that access to healthcare differs by race [60], age [61], gender [62], and many other factors [63, 64]. Some subpopulations experience barriers to care and healthcare [65]. Batty et al. [66] report that policies targeted to low-income subpopulations might have larger impacts than for other subpopulations in improving access to healthcare and health outcomes and mitigating the financial burdens of health events. Therefore, it is plausible that these factors may also potentially influence enrollment into health insurance or modify the impacts of health insurance. Using causal and instrumental forest methods, we explored the uptake of HI among OHIE lottery winners, and the effects of lottery selection and health insurance on a range of outcomes.

Previous descriptive work by Allen et al. [31] noted that less than one-third of the 29,411 individuals selected from the list ended up enrolled in Oregon Health Plan Standard, with 61 percent of these winners submitting applications and 30 percent approved for coverage. The authors argue that the imperfect take-up of the Oregon Health Plan Standard coverage was attributable both to ineligibility and to difficulty obtaining coverage for the truly eligible. Our analysis extends this work by examining individual level effects of lottery selection on subsequent HI uptake, and exploring heterogeneity by subgroups. Our findings indicate that the probability of enrollment in the insurance plan among winners of the OHIE lottery is heterogeneous, varying across age-based and race-based subgroups as well as by whether the individual had ‘high risk’ diagnoses or depression. Such heterogeneity raises important questions for policy makers regarding equity of access and the targeting of health insurance to those that may be most in need. There are a number of possible explanations for these differences in uptake. Firstly, it may be the case that some winners of the OHIE failed to apply for enrollment and that this differed with regard to the individuals characteristics. Of the initial survey respondents who could have applied but did not, 23 percent reported that they believed their income or assets to be too high, while a third attributed their failure to apply to not having completed the application, finding the paperwork a hassle, or not having the appropriate documentation [31]. In our sample with higher response rates, we found broadly similar results with almost 40 percent of the respondent winners reporting they failed to apply because of complications regarding the paperwork, thus some of the heterogeneity of HI uptake among the subgroups may be attributable to language barriers. Another possibility is that these winners did apply but had a higher probability of having their applications rejected. Allen et al. [31] report that a third of those who submitted applications failed to return all of the necessary documentation in time, while 55 percent of submitted applications were denied on the basis of excessive income or assets. Our findings also indicate that denial decisions are mostly related to income issues and paperwork. We explored the extent to which heterogeneity in uptake arises through heterogeneity in applications or approvals by fitting two additional causal forests to estimate the effects of lottery selection on the probability of (a) applying for insurance and (b) having the application approved. The findings from these forests extend the understanding of HI uptake heterogeneity by showing that age-based heterogeneity arises predominantly at the application stage while race-based heterogeneity is more evident at the approval stage. While this is a striking finding, further research is required to understand whether these differences represent inequitable treatment of individuals or are justified on the basis of individuals’ circumstances. Reducing administrative barriers such as the paperwork requirements may increase uptake of HI, and to the extent that these barriers differentially impact population subgroups they may also serve to narrow disparities in uptake. However, the lack of power of the IV estimates limits the extent to which firm policy recommendations can be made While we find that HI is beneficial for almost all groups for outcomes such as the number of office visits, and the number of prescription drugs, its impact appears on some outcomes to be significant only for particular subgroups. For instance, the impact on the number of hospital admissions is found to be statistically significant solely for the white-female and depressed subgroups. While such findings warrant further research. Triangulation with estimates from analyses from larger, albeit non-randomized, datasets may be informative as to the extent to which these subgroup effects are spurious.

This paper also contributes to the growing body of evidence on the overall impact of lottery selection and Medicaid coverage on health, healthcare utilization, and financial hardship outcomes by exploiting the randomized controlled trial settings of OHIE data [25, 27, 39, 67]. Our findings of limited heterogeneity in the effects of the lottery and health insurance are broadly consistent with those of other published studies that considered a more limited range of subgroups/outcomes. Dennett & Baicker [43] conclude that there is little evidence of heterogeneity with respect to neighborhood characteristics. Qiu et al. [41] find some evidence of heterogeneity with respect to age for happiness but for other outcomes (mental component score, physical component score, and out of pocket spending) the evidence for heterogeneity is fairly weak. Tidemann [42] used quantile regression to examine heterogeneous of effects of HI on self-reported household expenditures, but considered only two subgroups that are based on health concerns and depression and focused on a single outcome, financial strain, and also used a different sample to our paper and the other two studies. It should be noted that heterogeneity may emerge over a longer time horizon than considered in these studies.

Several limitations of this study are noteworthy in terms data and methods. First, as reported in the original study [22], the estimated effects are short-term, while the effect of insurance on a particular outcome might appear after several years. However, given that data was not collected beyond two years, this is a common issue across all the studies of the OHIE. Second, the subgroups that we have considered are based on a set of baseline characteristics that are suspected to have an impact on the HI-effect. However, there might be unconsidered subgroups for whom there are heterogeneous effects. Furthermore, subgroups analyses generally suffer from false discoveries due to multiple comparisons [68] and here we consider a number of outcome and subgroups. Post-hoc corrections such as that proposed by Bonferroni [69] could be used to address this concern but tend to be conservative, correcting the p-values in line with the number of tests undertaken. Finally, analyzing the reasons for application denial and application non-submission has been conducted on a limited, non-representative subset of the data owing to a substantial percentage of missing values. Regarding the methods, causal forests generally perform less well under sparsity which motivates the use of methods such as Shrinkage Bayesian Forest [70]. Secondly, the used AIPW estimator may give extreme weights to some observations, however this is less of concern here given the OHIE design. Moreover, the results may be sensitive to the choice of tuning parameters, but our findings are found to be robust when opting to tune all, some, and none of the parameters. Furthermore, the instrumental variable approach assumes there is no essential heterogeneity (i.e., selection based on gains) this would be violated if individuals that enroll in HI are those that anticipate larger gains from being insured. Since individuals are drawn from a waiting list, this assumption may be more plausible here than in other settings. Moreover, we cannot be confident that the variability in point estimates in the IV analysis represents heterogeneity, rather than imprecision in estimates given the width of confidence intervals as it is well known that IV studies suffer from lower power [71]. Moreover, the confidence intervals of Medicaid’s effects on many health outcomes may include clinically significant impacts even if they are statistically insignificant [22]. These issues could be addressed by taking advantage of administrative data with much larger sample sizes. However, it should be noted that these data would not encompass all the outcomes that we have studied in this paper. Notwithstanding these limitations, this paper demonstrates the applicability of causal forests and instrumental forests to explore heterogeneous effects of health policies.

Overall our study explores heterogeneity in uptake and outcomes of HI. We detect some differences between population subgroups, particularly in relation to uptake of HI. In terms of our results of HI on health related outcomes in the short run, our results broadly support the findings of the original studies which focused on aggregate effects.

## Supporting information

S1 File Section1.1. Methodology: Instrumental forest analysis. (PDF)

S2 File Section1.2. Results: Instrumental forest analysis.
(PDF)

S3 File Section2.1. Test calibration for intent-to-treat analysis.
(PDF)

S4 File Section2.2. Rank-Weighted Average Treatment Effect (RATE) for insurance-effect analysis.
(PDF)

S5 File Section3.1. Analysis of larger dataset. (PDF)

S6 File Section3.2. Analysis lottery draws.
(PDF)

S1 Fig Individualized treatment effects (standardized to have the same scale for comparison purposes) of lottery selection (intent-to-treat analysis) estimated using causal forest, and effects of health insurance estimated using instrumental forest. (TIF)

S2 Fig Forest plot for subgroups’ conditional average treatment effects of lottery selection and health insurance on mental component score. (TIF)

S3 Fig Forest plot for subgroups’ conditional average treatment effects of lottery selection and health insurance on physical component score. (TIF)

S4 Fig Forest plot for subgroups’ conditional average treatment effects of lottery selection and health insurance on amount of out-of-pocket spending ($). (TIF)

S5 Fig Forest plot for subgroups’ conditional average treatment effects of lottery selection and health insurance on number of prescription drugs. (TIF)

S6 Fig Forest plot for subgroups’ conditional average treatment effects of lottery selection and health insurance on number of office visits. (TIF)

S7 Fig Forest plot for subgroups’ conditional average treatment effects of lottery selection and health insurance on number of hospital admissions. (TIF)

S8 Fig Forest plot for subgroups’ conditional average treatment effects of lottery selection and health insurance on number of out-patient surgery visits. (TIF)

S9 Fig Forest plot for subgroups’ conditional average treatment effects of lottery selection and health insurance on number of emergency department visits. (TIF)

S10 Fig Subgroup effects of lottery selection on the probability of OHP standard uptake. (TIF)

S11 Fig Targeting operator characteristic curve evaluated on mental component score, physical component score, amount of out-of-pocket spending, and number of prescription drugs from health insurance. (TIF)

S12 Fig Targeting operator characteristic curve evaluated on number of office visits, hospital admissions, outpatient surgery visits, and emergency department visits from health insurance. (TIF)

S13 Fig Forest plot for subgroups’ conditional average treatment effects of lottery selection on OHP uptake using in person and mail survey datasets. (TIF)

S14 Fig Forest plot for subgroups’ conditional average treatment effects of insurance on amount of out-of-pocket spending using in-person and mail survey datasets. (TIF)

S15 Fig Enrollment rate over the eight lottery draws. (TIF)

S16 Fig Distribution of baseline characteristics over the eight lottery draws. (TIF)

S1 Table Overall effects of health insurance using instrumental forest. (TIF)

S2 Table Subgroups’ conditional average treatment effects of health insurance estimated using instrumental forest. (TIF)

S3 Table Variable importance scores for all covariates in each analysis where percentages (bold indicates importance > 20% of the mean importance). (TIF)

S4 Table Variable importance scores for retained covariates (i.e. those with importance > 20% of the mean importance) in each analysis. (TIF)

S5 Table Summary of self-reported reasons for not applying to OHP or denial of their application for the people in our sample who returned their initial mail survey and received an OHP application form. (TIF)

S6 Table Calibration test for intent-to-treat analysis. (TIF)

S7 Table RATE estimates and standard errors. (TIF)

S1 Data (RAR)
