# Supplementary material for: Heterogeneity within the Oregon Health Insurance Experiment: An application of causal forests
Source: PLoS One. 2024 Jan 18;19(1):e0297205. doi: 10.1371/journal.pone.0297205 (PMC10796043; doi:10.1371/journal.pone.0297205)
Supplement: S1 File — (PDF) [file pone.0297205.s001.pdf]

Supplement Appendix:

S1. Analysis of effects of health insurance coverage

S1.1 Methodology: instrumental forest analysis

As in the original study [1], lottery selection is used as an instrument for Medicaid coverage (HI) to estimate the causal effect of getting the insurance on the outcomes. We obtain individual level effect estimates using the IV forest approach. In contrast to intent-to-treat analysis which presumes two potential outcomes for each individual, the effect-of-health-insurance analysis intuitively assumes that each individual has four potential outcomes. The potential outcomes of individual i, $Y(Z, D)$, will be a function of D and Z, and there will be four of potential outcomes: $Y(Z=1, D=1)$ if the individual is selected in the lottery and received health insurance, $Y(Z=1, D=0)$ if he/she is selected in lottery and did not receive health insurance, $Y(Z=0, D=1)$ if he/she is not selected in lottery but received health insurance, and $Y(Z=0, D=0)$ if he/she is neither won the lottery nor received the insurance. The observed treatment will take the following form:

$$D_i = Z_i D_{1i} + (1 - Z_i) D_{0i} \quad \text{(S1)}$$

where the health insurance indicator D_i for individual i will be either the potential treatment assignment D_{1i} if he/she is selected in the lottery ($Z_i = 1$) or the potential treatment assignment D_{0i} otherwise ($Z_i = 0$). Thus the level of the IV is assumed to influence the treatment assignment unless D_{1i}= D_{0i}.

It is essential to check whether the lottery is a plausible instrument so that the causal effects are well defined as described in the context of OHIE by Johnson et. al[2]. This can be guaranteed if Z satisfies four assumptions. First, the lottery is correlated with Medicaid enrollment, which is apparent since winning the lottery increases the probability of enrollment [3]. Second, the lottery selection is not affected by unobserved confounders which is satisfied due to the fact that it is randomized. Third, that the data does not include individuals that did not win the lottery but had enrolled in the Medicaid. Approximately, 1.6% of the sample were affected by this issue but they were already enrolled in Medicaid before the lottery

winners were announced [2]. Fourth, the exclusion restriction of the lottery is met, which states that the lottery result does not otherwise influence the outcomes except through health insurance. It is fairly plausible that individuals do not substantively change their non-HI behaviours in ways that meaningfully influence their health in the short-term in response to the lottery result.

Under these assumptions and considerations, analogous parameters to those in the intent-to-treat section can be "locally" defined [4] where "locally" refers to the set of individuals who will enroll in Medicaid once they win the lottery (i.e. ‘compliers’). The estimands of interest in this context are the Local Average Treatment Effect (LATE), the Conditional Local Average Treatment Effects (CLATE) ($\tau^{Local}{}_i(x)$) and subgroups' Local Average Effects (GLATE):

$$LATE_{Insurance} = \mathbb{E}(\mathrm{Y_i}(D=1) - \mathrm{Y_i}(D=0)|D_{1i} > D_{0i})$$

$$= \frac{\mathbb{E}[\mathrm{Y_i}|\mathrm{Z_i}=1] - \mathbb{E}[\mathrm{Y_i}|\mathrm{Z_i}=0]}{\mathbb{E}[\mathrm{D_i}|\mathrm{Z_i}=1] - \mathbb{E}[\mathrm{D_i}|\mathrm{Z_i}=0]}$$

(S2) $$= \frac{Cov\ (Y,Z)}{Cov\ (D,Z)} \quad = \frac{Intent\text{−}To\text{−}Treat\ Effect}{Proportion\ of\ Compliers}$$

(S3) $$\tau^{Local}{}_i(x) = \frac{Cov(\mathrm{Y_i}, \mathrm{Z_i}|\boldsymbol{X}_i = x)}{Cov(\mathrm{D_i}, \mathrm{Z_i}|\boldsymbol{X}_i = x)}$$

(S4) $$GLATE_{Insurance} = \mathbb{E}\left(\tau^{Local}{}_i \middle| G_i = g\right), G_i \subset \boldsymbol{X}_i$$

where $Cov()$ denotes the covariance of the arguments.

The Instrumental Forest algorithm is analogous to the causal forests described in the main text and can be described in two alternative ways: using $\tau^{Local}{}_i(x)$ as the variable to be considered in (S4) instead of τ as shown in [5–7], which is the approach taken by this study, or by computing the intent-to-treat effect then correcting for the proportion of the compliers as conducted in [4].

The instrumental forest provides estimates of the Average (Conditional) LATE, $\tau(x)$. To obtain doubly robust estimates of the GATE, two regression forests are grown to obtain estimates of (i) the propensity scores $\pi_i(x)$ and (ii) the variance of propensities, $\sigma^2 = (D_i - \pi_i(x))^2$. These are then used to obtain

debiasing weights of the form $D_i - \hat{\pi}_i(x)/\widehat{\sigma^2}$. Next a causal forest is used to estimate compliance scores for each individual. Finally, the estimand is calculated using the fitted instrumental forest given the estimated debiasing weights and compliance scores of the targeted subsample.

References

1. Baicker K, Taubman SL, Allen HL, Bernstein M, Gruber JH, Newhouse JP, et al. The Oregon Experiment — Effects of Medicaid on Clinical Outcomes. N Engl J Med. 2013 May 2;368(18):1713–22.

2. Johnson M, Cao J, Kang H. Detecting Heterogeneous Treatment Effect with Instrumental Variables. ArXiv190803652 Stat [Internet]. 2021 Jan 19 [cited 2022 Jan 24]; Available from: http://arxiv.org/abs/1908.03652

3. Finkelstein A, Taubman S, Wright B, Bernstein M, Gruber J, Newhouse JP, et al. The Oregon Health Insurance Experiment: Evidence from the First Year*. Q J Econ. 2012 Aug 1;127(3):1057–106.

4. Bargagli Stoffi FJ, Gnecco G. Causal tree with instrumental variable: an extension of the causal tree framework to irregular assignment mechanisms. Int J Data Sci Anal. 2020 Apr;9(3):315–37.

5. Athey S, Tibshirani J, Wager S. Generalized Random Forests. ArXiv161001271 Econ Stat [Internet]. 2018 Apr 5 [cited 2020 Jul 21]; Available from: http://arxiv.org/abs/1610.01271

6. Chen J er, Hsiang CW. Causal Random Forests Model Using Instrumental Variable Quantile Regression. Econometrics. 2019 Dec 16;7(4):49.

7. Wang G, Li J, Hopp WJ. An Instrumental Variable Forest Approach for Detecting Heterogeneous Treatment Effects in Observational Studies. Manag Sci. 2021 Sep 13;mnsc.2021.4084.
