# Supplementary material for: Heterogeneity within the Oregon Health Insurance Experiment: An application of causal forests
Source: PLoS One. 2024 Jan 18;19(1):e0297205. doi: 10.1371/journal.pone.0297205 (PMC10796043; doi:10.1371/journal.pone.0297205)
Supplement: S2 File — Results: Instrumental forest analysis. (PDF) [file pone.0297205.s002.pdf]

Supplement Appendix:

S1. Analysis of effects of health insurance coverage

S1.2 Results: instrumental forest analysis

Table A1 shows the overall effects of health insurance on the eight outcomes using the lottery as an instrument and applying the instrumental forest method. The table also contains the findings of the original study [1] for comparison.

Table A1. Overall effects of health insurance using instrumental forest

	Effect of Health Insurance (95% CI)							
**Outcome**	**Mental component score**	**Physical component score**	**Amount of out-of-pocket spending ($)**	**Number of prescription drugs**	**Number of office visits**	**Number of hospital admissions**	**Number of out-patient surgery visits**	**Number of emergency department visits**
**Original study overall effect**	1.95<br>(0.03, 3.88)	1.20<br>(-0.54, 2.93)	-215<br>(-409, -22)	0.66<br>(0.21, 1.11)	2.70<br>(0.91, 4.49)	0.07<br>(-0.03, 0.17)	0.03<br>(-0.03, 0.09)	0.09<br>(-0.23, 0.42)
**IVF Analysis LATE**	1.09<br>(-1.33, 3.5)	-3.02<br>(-5.94,-0.10)	-233<br>(-318, -148)	0.35<br>(-0.013, 0.71)	1.35<br>(0.33, 2.37)	0.019<br>(-0.056, 0.094)	0.019<br>(-0.009, 0.048)	0.074<br>(-0.20, 0.35)

The instrumental forest estimates differ in a number of respects from the findings of the original study, although it is important to note that in the presence of heterogenous treatment effects 2SLS does not identify the local average treatment effect (LATE). There is weaker evidence of an impact on the mental health component score and evidence of a significant reduction in the physical health component score (-3.02, 95% CI: -5.94 to -0.10) while the original study predicted it to be not significant (1.20, 95% CI: -0.54 to 2.93). The estimated reduction in out-of-pocket expenditure while similar in magnitude is more precisely estimated using the IV forest method than the 2SLS approach reported in the original study as is the case for a number of the other outcomes (number of prescription drugs, office visits and surgery visits). The instrumental forest generally predicted lower overall effects than the original study.

Figure A1 reports the distributions of individualized effects of HI obtained using the IV forest. For almost all of the outcomes the range of individualized effects are more dispersed than the effects of the lottery. This may reflect greater estimation uncertainty attributable to the IV method rather than true underlying uncertainty. We therefore assess whether the effects for our pre-specified subgroups reveal significant differences across groups.

Table A2 and the right hand panels of Figures A2 to A9 report the GATEs for the effect of HI for each of our pre-specified subgroups. While there is a reasonable degree of variability in the estimates by subgroup, the confidence intervals tend to be quite wide (a common problem with IV analyses which suffer from reduced power), meaning we cannot be confident that there is subgroup heterogeneity.

Table A2. Subgroups' conditioanl average treatment effects of health insurance estimated using instrumental forest

	Effect of Health Insurance (95% CI)							
**Outcome / Subgroup**	**Mental component score**	**Physical component score**	**Amount of out-of-pocket spending ($)**	**Number of prescription drugs**	**Number of office visits**	**Number of hospital admissions**	**Number of out-patient surgery visits**	**Number of emergency department visits**
**Overall**	1.09<br>(-1.33, 3.5)	-3.02<br>(-5.94,-0.10)	-233<br>(-318, -148)	0.35<br>(-0.01, 0.71)	1.35<br>(0.33, 2.37)	0.019<br>(-0.056, 0.094)	0.019<br>(-0.009, 0.048)	0.074<br>(-0.20, 0.35)
**Males**	0.016<br>(-3.03, 3.06)	-1.41<br>(-2.97, 0.14)	-177<br>(-339, -14)	0.32<br>(-0.15, 0.79)	0.26<br>(-1.66, 2.19)	0.011<br>(-0.095, 0.117)	0.008<br>(-0.038, 0.053)	0.133<br>(-0.318, 0.586)
**Females**	1.91<br>(-1.65, 5.48)	-4.26<br>(-9.29, 0.77)	-276<br>(-357, -194)	0.37<br>(-0.16, 0.90)	2.19<br>(1.16, 3.22)	0.025<br>(-0.08, 0.13)	0.029<br>(-0.008, 0.065)	0.028<br>(-0.319, 0.374)
**19-34 years**	3.60<br>(-2.99, 10.18)	-6.10<br>(-14.17, 1.96)	-180<br>(-358, -3)	-0.03<br>(-0.97, 0.91)	1.54<br>(-0.97, 4.05)	0.031<br>(-0.167, 0.230)	0.010<br>(-0.055, 0.076)	-0.09<br>(-0.84, 0.66)
**34-49 years**	-0.80<br>(-2.0, 0.39)	-1.77<br>(-2.91, -0.64)	-225<br>(-339, -112)	0.48<br>(0.22, 0.75)	1.08<br>(-0.02, 2.17)	0.022<br>(-0.036, 0.080)	0.030<br>(-0.004, 0.065)	0.17<br>(-0.007, 0.347)
**49-64 years**	0.36<br>(-0.82, 1.53)	-0.70<br>(-1.70, 0.30)	-309<br>(-445, -174)	0.65<br>(0.29, 1.01)	1.49<br>(0.47, 2.50)	-0.001<br>(-0.055, 0.053)	0.017<br>(-0.021, 0.054)	0.158<br>(-0.005, 0.321)
**White**	-0.19<br>(-0.95, 0.57)	-1.58<br>(-2.30, -0.87)	-289<br>(-362, -216)	0.66<br>(0.49, 0.84)	2.0<br>(1.17, 2.83)	0.074<br>(0.015, 0.132)	0.027<br>(0.002, 0.052)	0.184<br>(0.033, 0.335)
**Non-white**	3.89<br>(-3.64, 11.41)	-6.18<br>(-15.38, 3.02)	-109<br>(-326, 108)	-0.35<br>(-1.43, 0.74)	-0.06<br>(-2.77, 2.64)	-0.102<br>(-0.305, 0.102)	0.002<br>(-0.070, 0.075)	-0.167<br>(-0.99, 0. 66)
**High risk**	-0.21<br>(-1.48, 1.06)	-1.11<br>(-2.24, 0.01)	-386<br>(-515, -258)	0.57<br>(0.2, 0.94)	0.86<br>(-0.53, 2.25)	0.049<br>(-0.046, 0.144)	0.021<br>(-0.026, 0.068)	0.095<br>(-0.111, 0.301)
**Non high risk**	1.57<br>(-1.70, 4.84)	-3.73<br>(-7.7, 0.25)	-176<br>(-281, -71)	0.27<br>(-0.21, 0.74)	1.54<br>(0.24, 2.83)	0.008<br>(-0.089, 0.105)	0.019<br>(-0.016, 0.054)	0.066<br>(-0.306, 0.439)
**Depressed**	0.20<br>(-0.95, 1.36)	-1.73<br>(-2.77, -0.69)	-221<br>(-363, -78)	0.73<br>(0.43, 1.03)	2.23<br>(0.85, 3.61)	0.097<br>(0.014, 0.181)	0.018<br>(-0.021, 0.057)	0.26<br>(0.009, 0.518)
**Non-depressed**	1.54<br>(-2.07, 5.15)	-3.69<br>(-8.09, 0.71)	-239<br>(-344, -134)	0.15<br>(-0.37, 0.68)	0.90<br>(-0.47, 2.27)	-0.022<br>(-0.13, 0.084)	0.020<br>(-0.018, 0.059)	-0.024<br>(-0.424, 0.376)
**19-34 years females**	4.92<br>(-4.53, 14.36)	-8.74<br>(-22.18, 4.70)	-172<br>(-318, -26)	-0.10<br>(-1.43, 1.23)	2.80<br>(0.75, 4.84)	-0.021<br>(-0.292, 0.249)	0.011<br>(-0.066, 0.088)	-0.26<br>(-1.15, 0.63)
**34-49 years females**	0.093<br>(-1.32, 1.51)	-2.36<br>(-3.95, -0.77)	-275<br>(-406, -144)	0.53<br>(0.19, 0.86)	2.24<br>(0.56, 3.92)	0.062<br>(-0.012, 0.137)	0.066<br>(0.014, 0.117)	0.240<br>(-0.005, 0.484)
**49-64 years females**	0.20<br>(-1.37, 1.78)	-0.65<br>(-1.99, 0.68)	-419<br>(-562, -276)	0.79<br>(0.26, 1.34)	1.3<br>(-0.03, 2.64)	0.039<br>(-0.036, 0.114)	0.003<br>(-0.044, 0.051)	0.140<br>(-0.068, 0.349)
**19-34 years males**	1.72<br>(-6.89, 10.32)	-2.35<br>(-6.40, 1.70)	-193<br>(-571, 185)	0.07<br>(-1.19, 1.33)	-0.26<br>(-5.60, 5.09)	0.106<br>(-0.183, 0.396)	0.009<br>(-0.106, 0.124)	0.150<br>(-1.14, 1.44)
**34-49 years males**	-1.89<br>(-3.9, 0.12)	-1.07<br>(-2.66, 0.53)	-165<br>(-358, 28)	0.43<br>(0.02, 0.84)	-0.34<br>(-1.63, 0.95)	-0.027<br>(-0.119, 0.065)	-0.012<br>(-0.057, 0.032)	0.085<br>(-0.170, 0.340)
**49-64 years males**	0.55<br>(-1.21, 2.32)	-0.75<br>(-2.26, 0.75)	-173<br>(-418, 73)	0.47<br>(0.02, 0.92)	1.71<br>(0.14, 3.28)	-0.050<br>(-0.128, 0.028)	0.033<br>(-0.028, 0.094)	0.180<br>(-0.078, 0.438)
**White females**	0.07<br>(-0.95, 1.09)	-1.47<br>(-2.34, -0.60)	-327<br>(-423, -232)	0.56<br>(0.32, 0.79)	2.47<br>(1.25, 3.69)	0.099<br>(0.039, 0.159)	0.043<br>(0.008, 0.079)	0.172<br>(-0.01, 0.355)
**Non-white females**	5.98<br>(-5.24, 17.2)	-10.43<br>(-26.47, 5.60)	-162<br>(-317, -7.5)	-0.05<br>(-1.65, 1.56)	1.58<br>(-0.35, 3.51)	-0.139<br>(-0.451, 0.172)	-0.004<br>(-0.091, 0.083)	-0.292<br>(-1.33, 0.746)
**White males**	-0.53<br>(-1.66, 0.61)	-1.73<br>(-2.92, -0.54)	-239<br>(-356, -126)	0.8<br>(0.53, 1.08)	1.38<br>(0.34, 2.43)	0.041<br>(-0.069, 0.150)	0.006<br>(-0.029, 0.041)	0.199<br>(-0.054, 0.453)

**Non-white males**	1.20	-0.72	-41	-0.73	-2.18	-0.053	0.011	-0.008
	(-8.16, 10.56)	(-4.93, 3.50)	(-494,412)	(-2.10, 0.64)	(-7.85, 3.49)	(-0.29, 0.185)	(-0.11, 0.134)	(-1.33, 1.32)

Figure A1. Individualized treatment effects (standardized to have the same scale for comparison purposes) of lottery selection (intent-to-treat analysis) estimated using causal forest, and effects of health insurance estimated using instrumental forest.

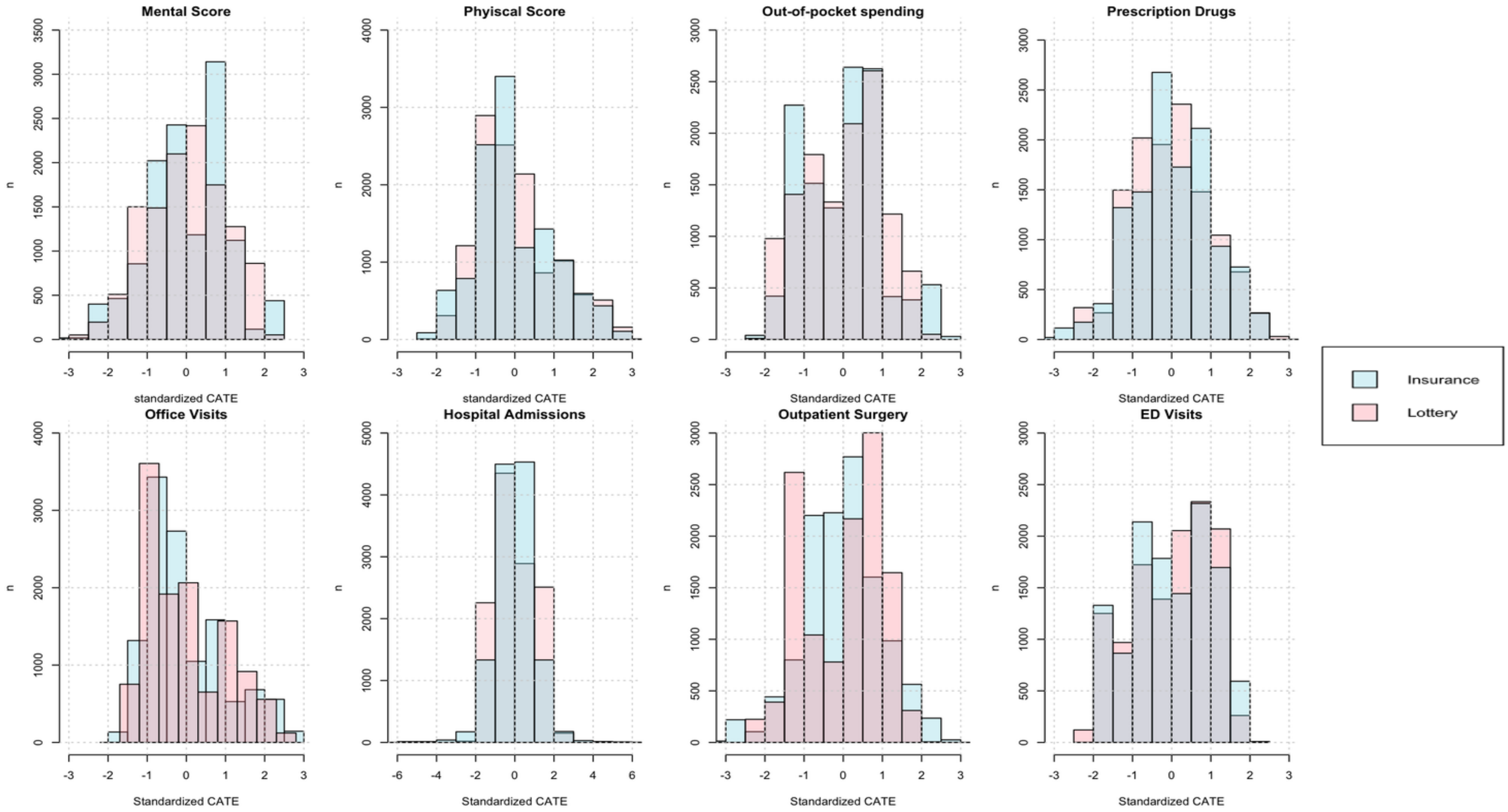

Figure A2. Forest plot for subgroups' conditional average treatment effects of lottery selection and health insurance on mental component score.

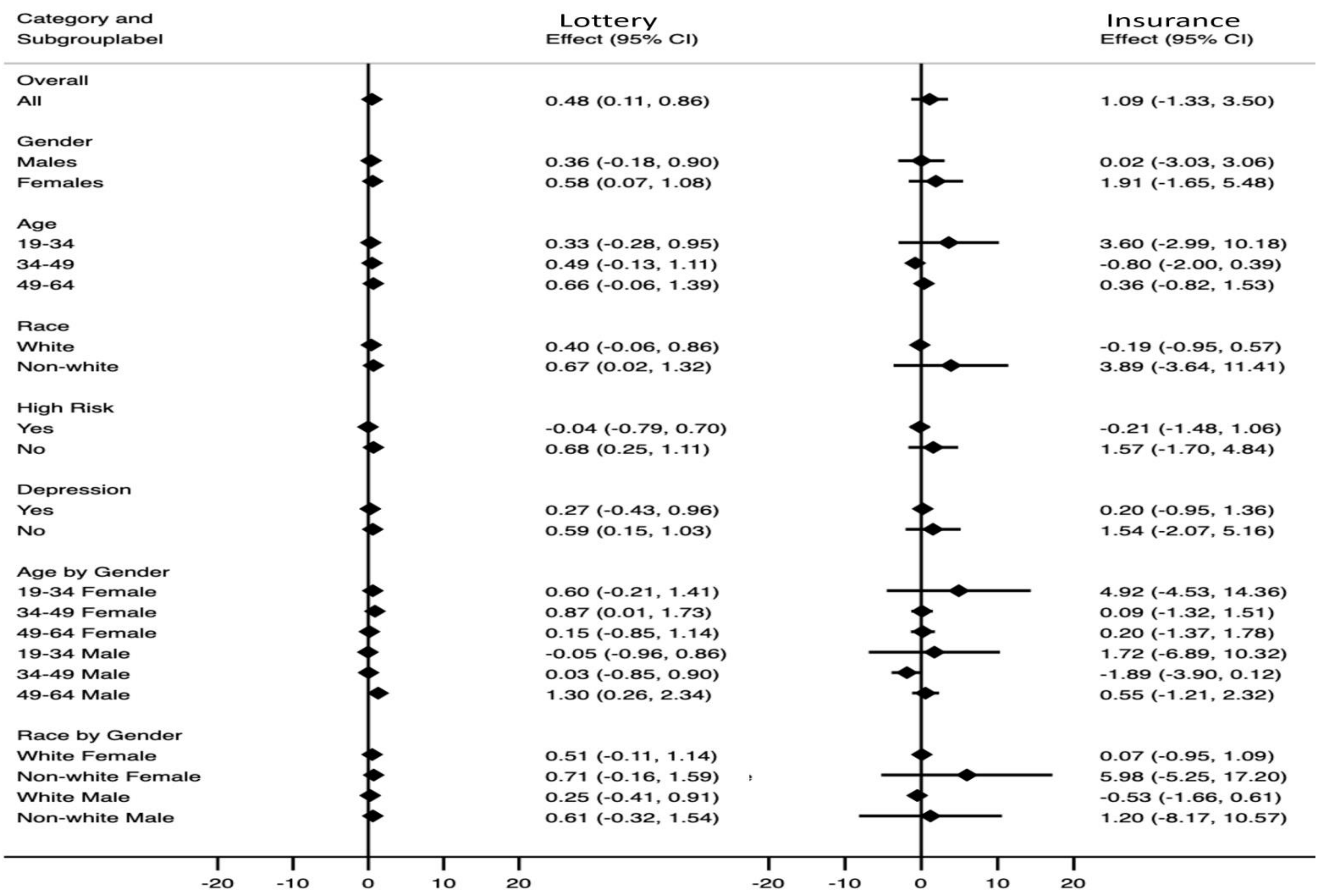

Figure A3. Forest plot for subgroups' conditional average treatment effects of lottery selection and health insurance on physical component score.

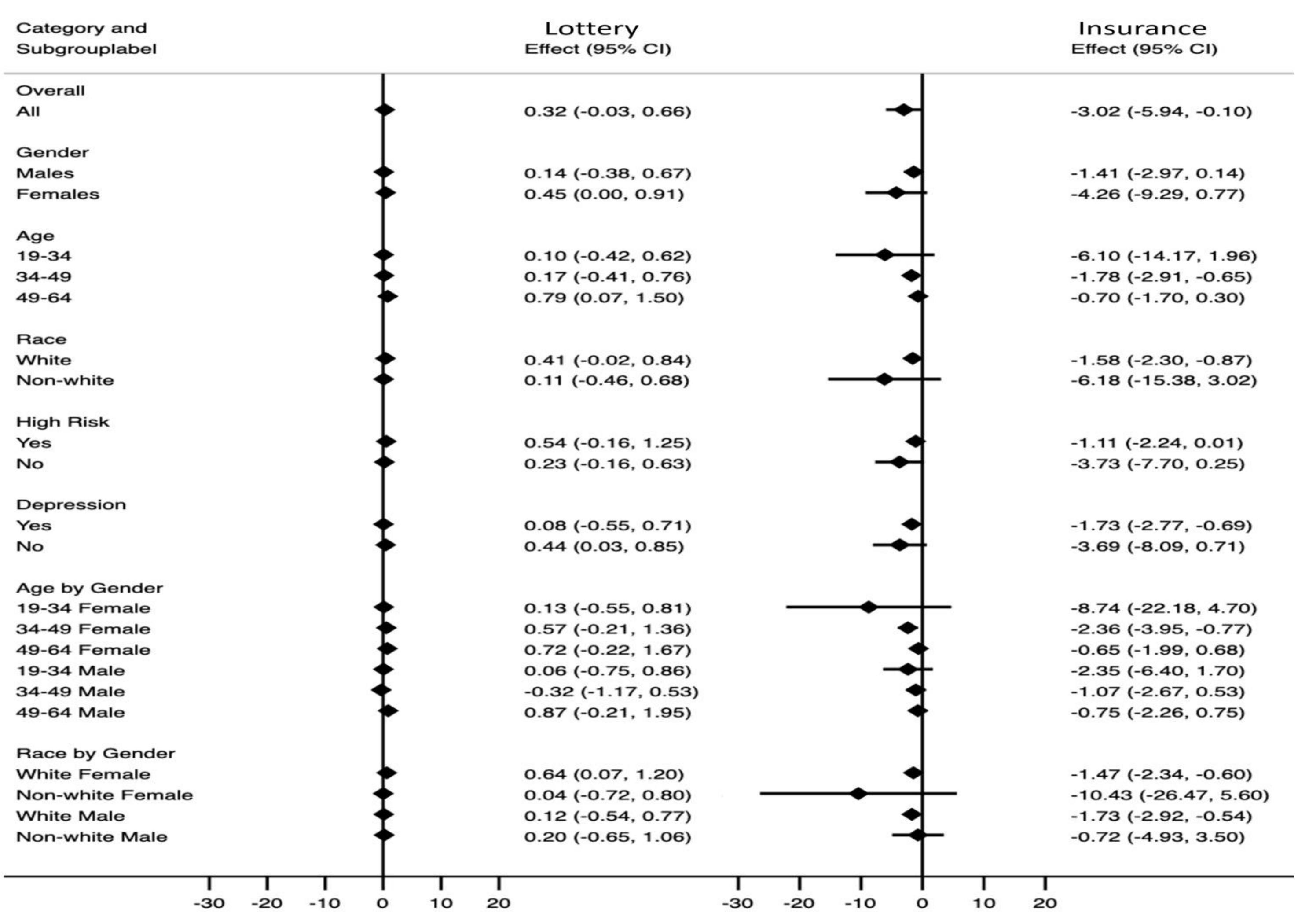

Figure A4. Forest plot for subgroups' conditional average treatment effects of lottery selection and health insurance on amount of out-of-pocket spending ($).

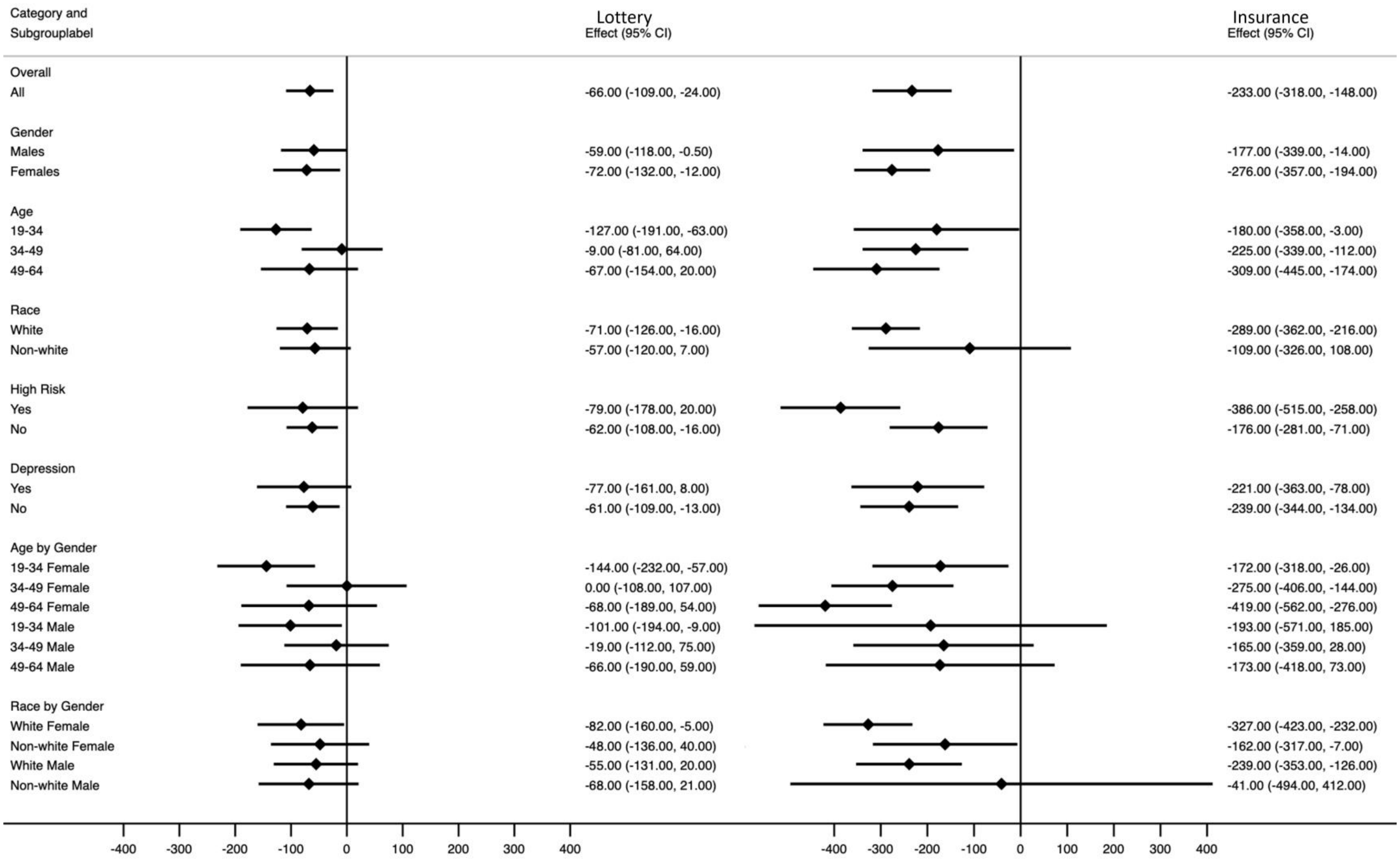

Figure A5. Forest plot for subgroups' conditional average treatment effects of lottery selection and health insurance on number of prescription drugs.

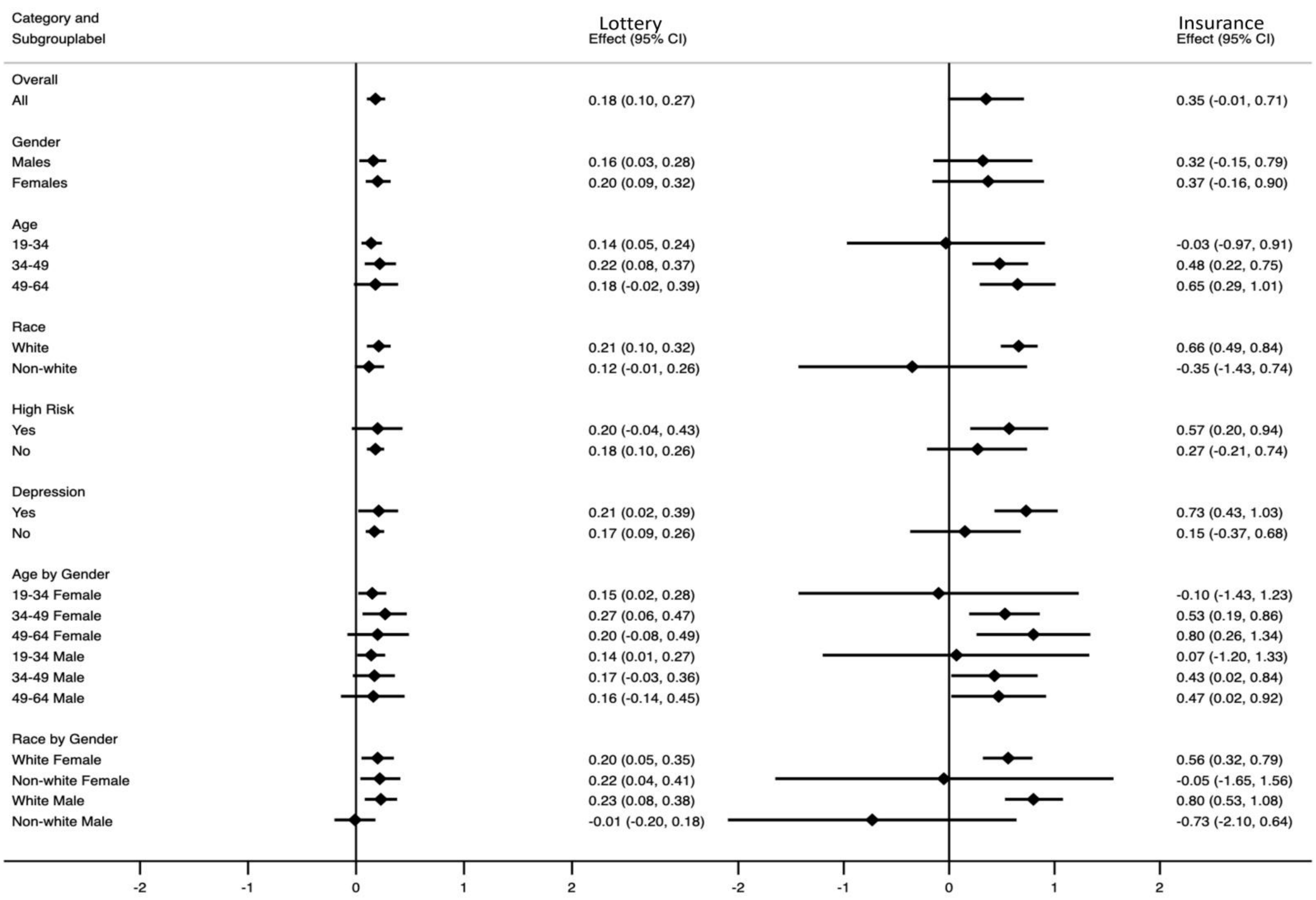

Figure A6. Forest plot for subgroups' conditional average treatment effects of lottery selection and health insurance on number of office visits.

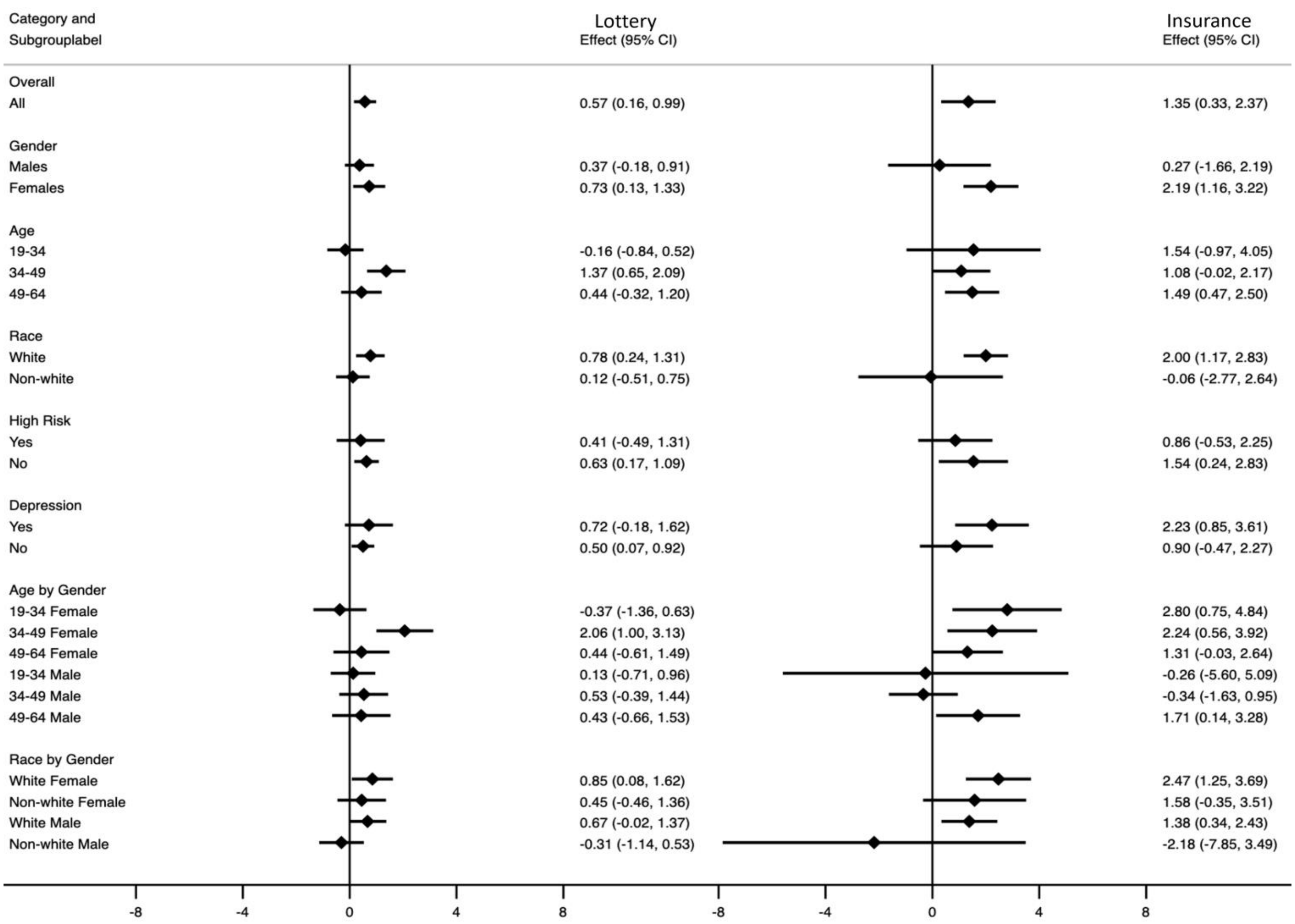

Figure A7. Forest plot for subgroups' conditional average treatment effects of lottery selection and health insurance on number of hospital admissions.

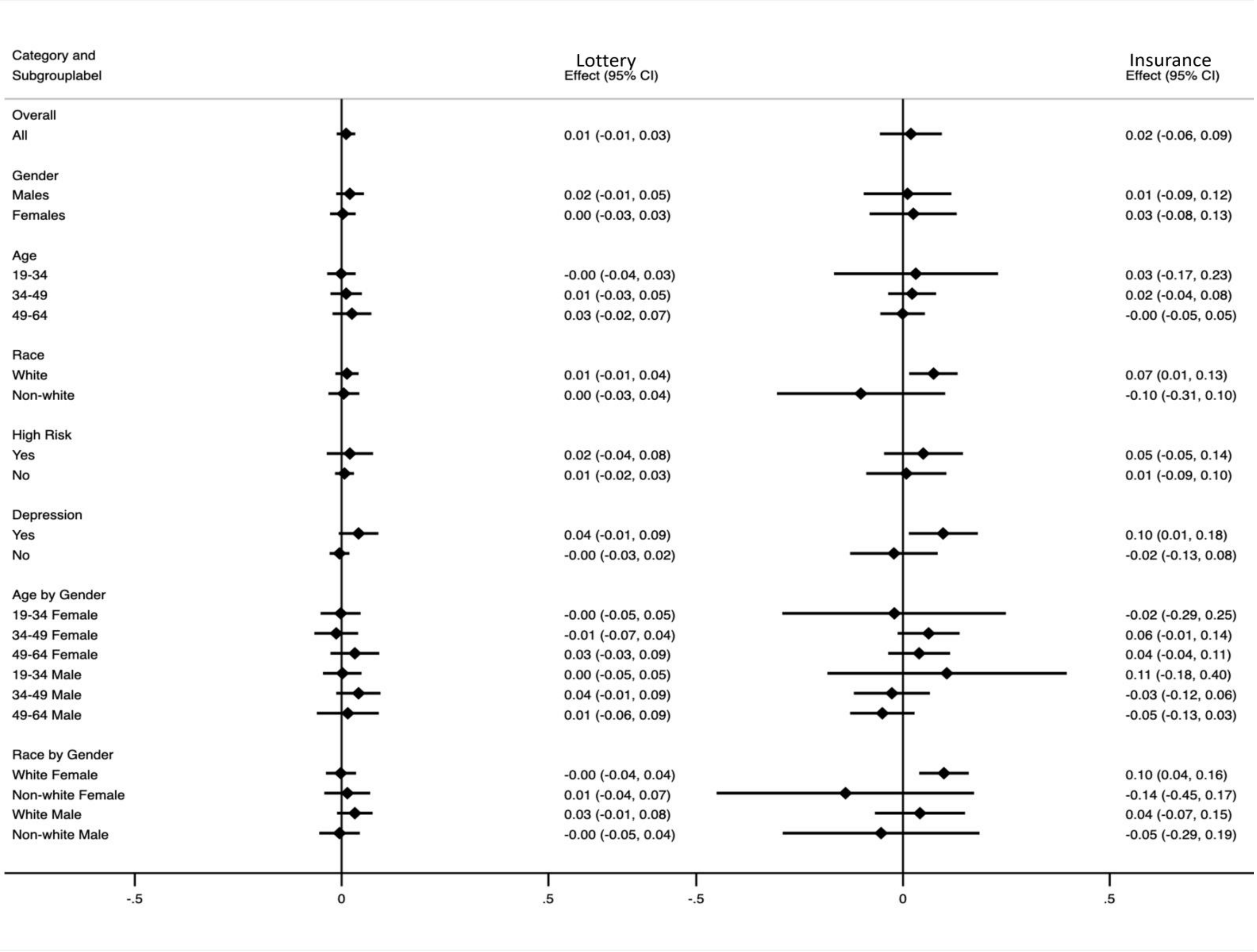

Figure A8. Forest plot for subgroups' conditional average treatment effects of lottery selection and health insurance on number of out-patient surgery visits.

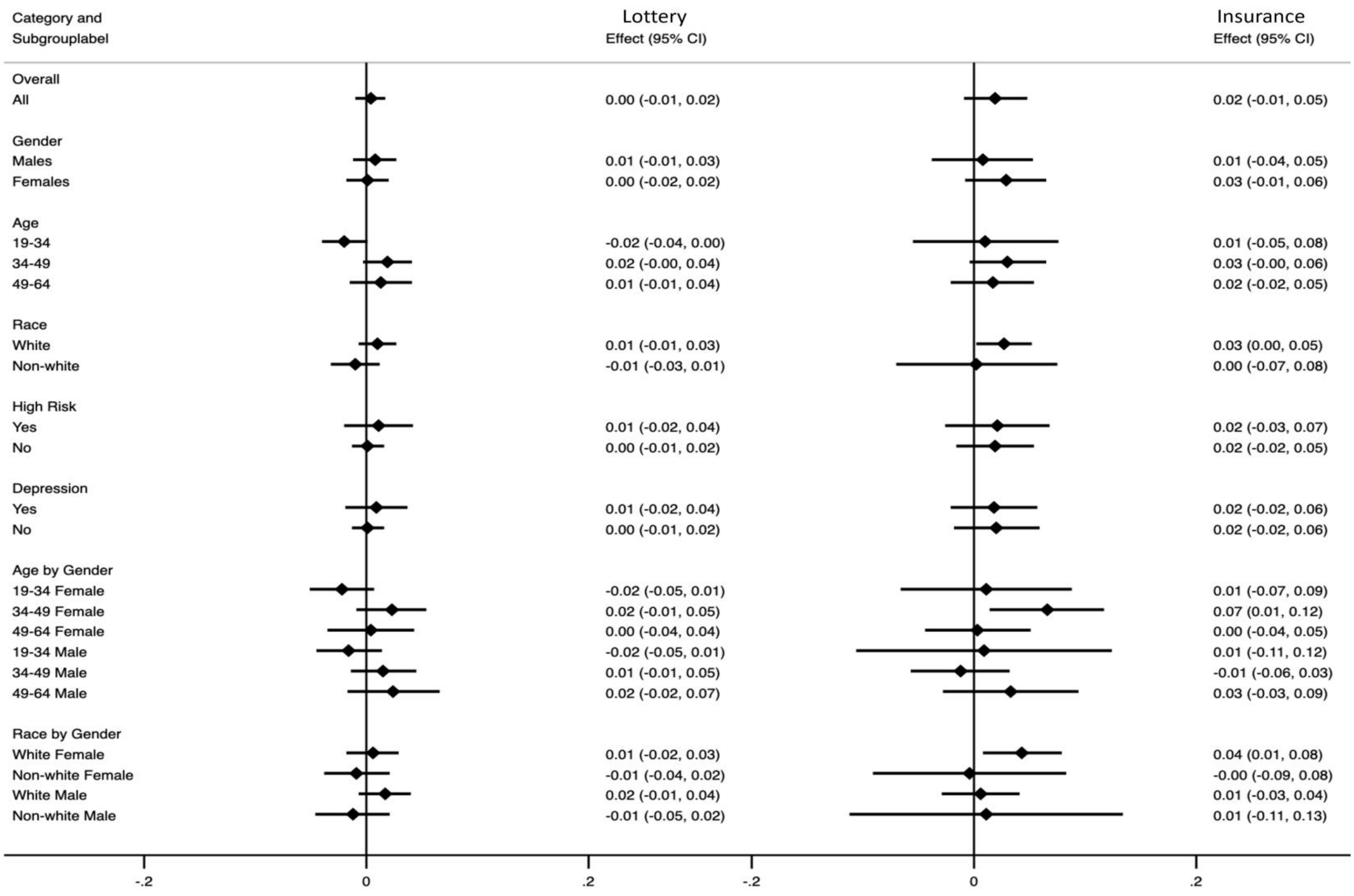

Figure A9. Forest plot for subgroups' conditional average treatment effects of lottery selection and health insurance on number of emergency department visits.

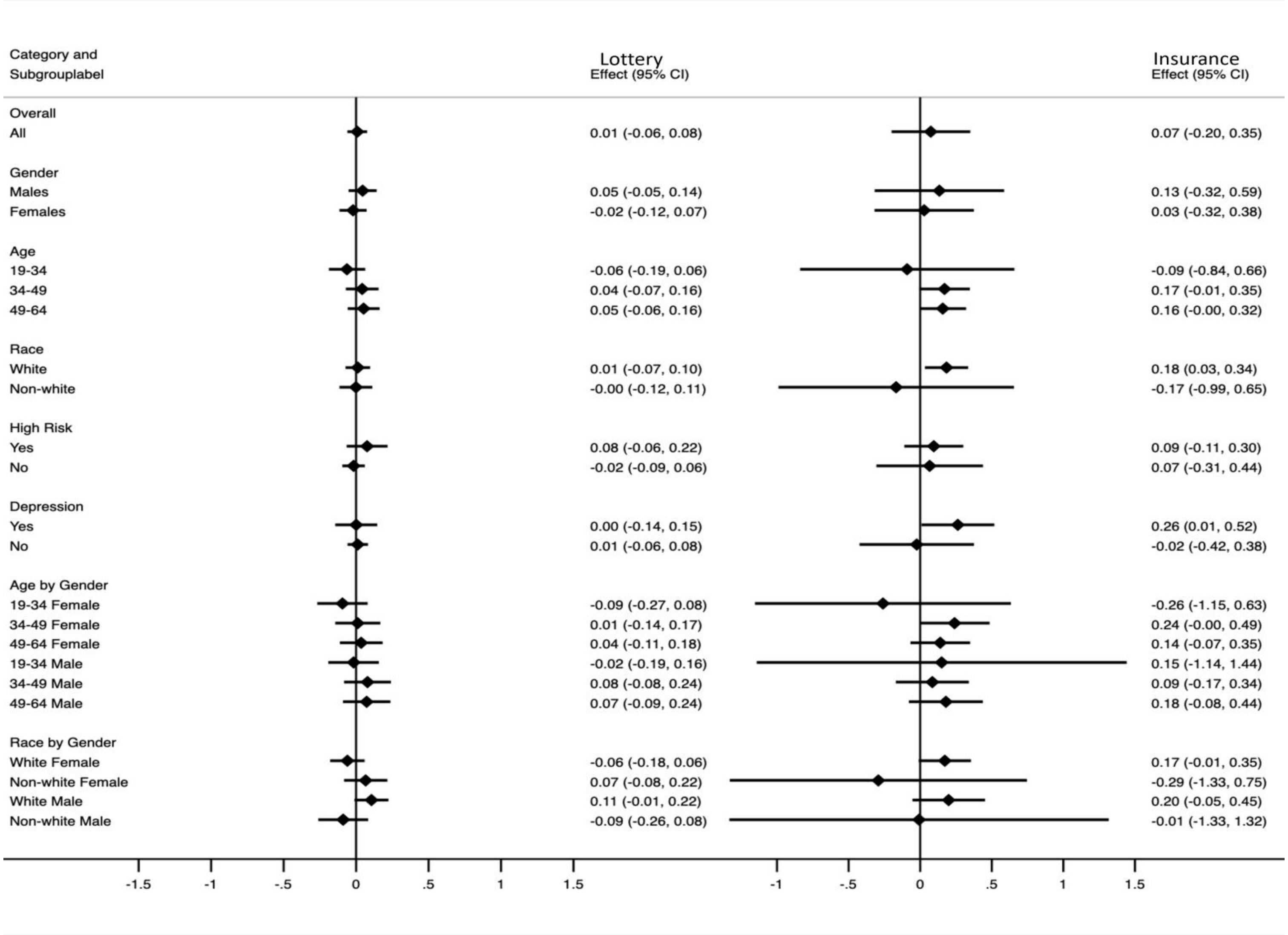

Figure A10. Subgroup effects of lottery selection on the probability of OHP standard uptake

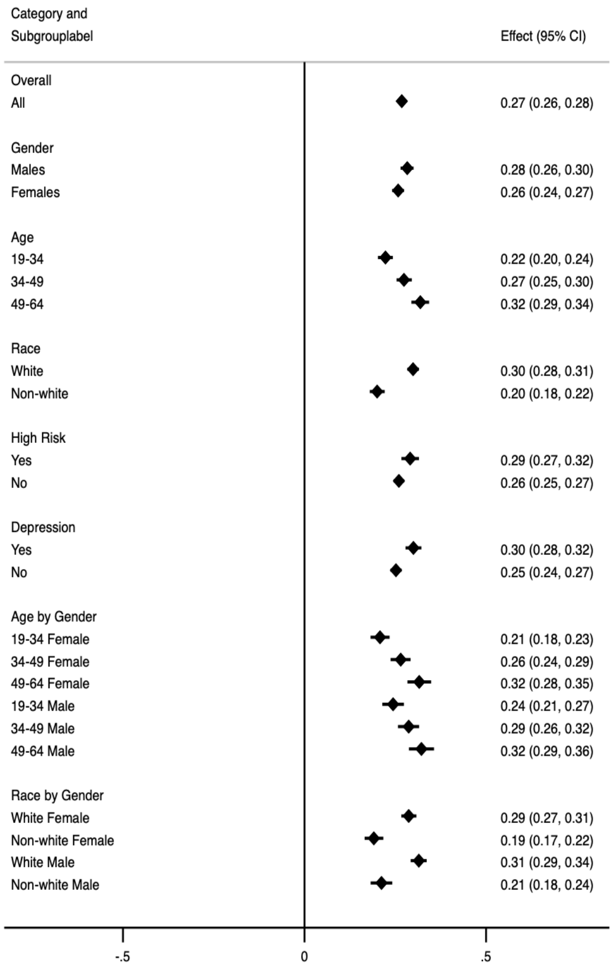

Table A3. Variable importance scores for all covariates in each analysis where percentages (bold indicates importance > 20% of the mean importance).

Outcome	Mental component score		Physical component score		Amount of out-of-pocket spending		No. prescription drugs		No. office visits		Hospital admissions		Outpatient surgery		Emergency department visits		OHP uptake
**Covariate**	**CF**	**INS**	**CF**	**INS**	**CF**	**INS**	**CF**	**INS**	**CF**	**INS**	**CF**	**INS**	**CF**	**INS**	**CF**	**INS**	**CF**
Household Size of 1	**3.5%**	**6.0%**	**3.4%**	**6.3%**	**2.8%**	**7.4%**	**3.0%**	**5.9%**	**3.5%**	**5.6%**	0.0%	**6.8%**	**4.6%**	**3.3%**	**2.7%**	**5.5%**	**9.1%**
Household Size of 2	**3.0%**	**6.3%**	**3.0%**	**6.1%**	**2.4%**	**6.6%**	**2.7%**	**5.8%**	**3.2%**	**5.7%**	0.0%	**6.4%**	**4.3%**	**3.5%**	**2.5%**	**5.4%**	**8.1%**
Household Size of 3	0.0%	0.0%	0.0%	0.0%	0.0%	0.0%	0.0%	0.0%	0.0%	0.0%	0.0%	0.0%	0.0%	0.0%	0.0%	0.0%	0.0%
Gender	**13.5%**	**12.6%**	**13.2%**	**12.5%**	**13.2%**	**11.9%**	**12.7%**	**12.5%**	**12.7%**	**12.2%**	0.0%	**11.7%**	**5.3%**	**5.1%**	**13.5%**	**12.3%**	**2.9%**
Black	0.6%	0.6%	0.8%	0.7%	0.7%	0.7%	0.4%	0.5%	0.6%	0.7%	0.0%	0.4%	**5.6%**	**7.6%**	0.6%	0.5%	0.7%
White	**12.1%**	**11.4%**	**11.9%**	**11.2%**	**12.1%**	**11.2%**	**11.5%**	**10.9%**	**11.9%**	**11.4%**	0.0%	**10.7%**	**5.4%**	**4.6%**	**12.2%**	**11.3%**	**5.3%**
Other Race	1.1%	0.8%	0.7%	0.7%	0.8%	**1.7%**	**0.8%**	**1.0%**	**1.7%**	0.7%	0.0%	0.8%	**4.7%**	**3.6%**	0.3%	0.4%	**2.1%**
Hispanic	**1.1%**	**1.0%**	**1.1%**	**1.0%**	**1.1%**	**1.5%**	0.6%	0.6%	**1.2%**	**1.1%**	0.0%	**1.1%**	**4.9%**	**2.5%**	0.7%	**0.6%**	**27.9%**
Preferred English Material	0.8%	**0.9%**	0.7%	0.6%	0.7%	0.6%	0.3%	0.4%	0.8%	0.5%	0.0%	0.4%	**4.0%**	**1.1%**	0.2%	0.2%	**25.7%**
Asthma	**1.1%**	**0.9%**	**1.8%**	**0.9%**	**1.2%**	**1.0%**	**1.4%**	**1.1%**	**0.9%**	**1.0%**	0.0%	**0.9%**	**5.2%**	**6.3%**	**1.1%**	**1.4%**	**1.3%**
Diabetes	0.6%	0.6%	**1.2%**	0.6%	0.7%	0.6%	**0.9%**	0.5%	0.6%	0.4%	0.0%	0.8%	**4.5%**	**4.7%**	0.2%	0.3%	0.3%
High Blood Pressure	**1.5%**	**1.6%**	**1.5%**	**1.6%**	**1.8%**	**1.6%**	**1.5%**	**2.0%**	**1.6%**	**1.5%**	0.0%	**2.7%**	**5.0%**	**5.8%**	**1.6%**	**1.2%**	0.7%
High Cholesterol	**1.2%**	**1.2%**	**1.3%**	**1.0%**	**1.2%**	**1.0%**	**1.2 %**	**1.4%**	**1.1%**	**1.8%**	0.0%	1.0%	**5.2%**	**5.2%**	**0.8%**	**0.9%**	0.5%
Heart Attack	0.0%	0.0%	0.1%	0.0%	0.0%	0.0%	0.0%	0.0%	0.1%	0.1%	0.0%	0.0%	**3.4%**	**3.8%**	0.0%	0.0%	0.0%
Congestive Heart Failure	0.0%	0.0%	0.0%	0.0%	0.0%	0.0%	0.0%	0.0%	0.0%	0.0%	0.0%	0.0%	0.3%	0.2%	0.0%	0.0%	0.0%
Emphysema/COPD	0.0%	0.0%	0.0%	0.0%	0.0%	0.0%	0.0%	0.0%	0.0%	0.0%	0.0%	0.0%	**3.5%**	**5.1%**	0.0%	0.0%	0.0%
Kidney Failure	0.0%	0.0%	0.0%	0.0%	0.0%	0.0%	0.0%	0.0%	0.0%	0.0%	0.0%	0.0%	**2.1%**	**2.3%**	0.0%	0.0%	0.0%
Cancer	0.0%	0.0%	0.0%	0.0%	0.0%	0.0%	0.0%	0.0%	0.0%	0.0%	0.0%	0.0%	**4.8%**	**8.1%**	0.0%	0.0%	0.1%
Depression	**12.4%**	**11.0%**	**12.5%**	**11.1%**	**12.0%**	**10.9%**	**12.8%**	**12.0%**	**12.2%**	**11.7%**	0.0%	**12.2%**	**5.3%**	**6.1%**	**12.5%**	**11.8%**	**3.3%**
High risk	**11.4%**	**10.8%**	**11.3%**	**10.7%**	**11.9%**	**10.4%**	**11.9%**	**10.9%**	**10.8%**	**10.9%**	0.0%	**10.5%**	**5.3%**	**4.9%**	**11.0%**	**10.3%**	**1.5%**
Age 19-34 years	**11.5%**	**10.8%**	**11.3%**	**11.4%**	**11.5%**	**10.8%**	**11.1%**	**10.0%**	**12.1%**	**10.9%**	0.0%	**10.7%**	**5.8%**	**6.0%**	**11.9%**	**11.1%**	**6.1%**
Age 34-49 years	**11.8%**	**11.6%**	**12.3%**	**11.5%**	**12.9%**	**11.1%**	**12.1%**	**11.6%**	**12.6%**	**12.6%**	0.0%	**11.0%**	**5.8%**	**5.3%**	**12.2%**	**11.8%**	**2.0%**
Age 49-64 years	**11.4%**	**10.5%**	**11.3%**	**10.6%**	**11.4%**	**10.3%**	**11.4%**	**10.5%**	**11.1%**	**10.7%**	0.0%	**10.4%**	**5.1%**	**4.7%**	**10.4%**	**11.2%**	**2.5%**

Table A4. Variable importance scores for retained covariates (i.e. those with importance > 20% of the mean importance) in each analysis

Outcome	Mental component score		Physical component score		Amount of out-of-pocket spending		No. prescription drugs		No. office visits		Hospital admissions		Outpatient surgery		Emergency department visits		OHP uptake
**Covariate**	**CF**	**INSF**	**CF**	**INSF**	**CF**	**INSF**	**CF**	**INSF**	**CF**	**INSF**	**CF**	**INSF**	**CF**	**INSF**	**CF**	**INSF**	**CF**
Household Size of 1	3.5%	6.4%	3.4%	6.4%	3.0%	7.1%	3.3%	6.3%	3.5%	5.9%	0.0%	8.3%	6.4%	5.3%	3.0%	5.8%	9.6%
Household Size of 2	3.5%	6.1%	3.2%	5.8%	2.7%	6.7%	2.7%	6.0%	3.2%	6.2%	0.0%	8.6%	6.6%	4.1%	2.6%	6.1%	8.3%
Gender	13.2%	13.0%	13.5%	12.7%	13.4%	12.4%	13.0%	12.7%	13.3%	12.6%	0.0%	11.3%	8.6%	8.2%	13.7%	12.8%	3.2%
Black	0.0%	0.0%	0.0%	0.0%	0.0%	0.0%	0.0%	0.0%	0.0%	0.0%	0.0%	10.6%	0.3%	5.6%	0.0%	0.0%	0.0%
White	12.3%	11.6%	12.2%	11.8%	12.6%	11.0%	11.7%	11.6%	11.9%	11.9%	0.0%	0.0%	8.0%	5.7%	12.3%	11.5%	5.2%
Other Race	0.9%	0.0%	0.0%	0.0%	0.0%	1.0%	0.5%	0.6%	0.8%	0.0%	0.0%	0.0%	1.4%	5.1%	0.0%	0.0%	2.3%
Hispanic	1.4%	1.7%	1.3%	1.3%	1.3%	1.4%	0.0%	0.0%	1.3%	1.2%	0.0%	1.2%	5.5%	3.5%	0.0%	0.0%	28.6%
Preferred English Material	0.0%	0.7%	0.0%	0.0%	0.0%	0.0%	0.0%	0.0%	0.0%	0.0%	0.0%	0.0%	0.3%	1.0%	0.0%	0.0%	24.9%
Asthma	1.2%	1.1%	1.3%	1.2%	1.3%	1.1%	1.5%	1.6%	1.2%	1.3%	0.0%	3.2%	7.8%	8.5%	1.7%	1.3%	1.5%
High Blood Pressure	1.7%	1.6%	2.7%	1.8%	2.1%	1.9%	2.4%	1.9%	2.7%	1.8%	0.0%	1.7%	7.4%	7.4%	1.5%	1.9%	0.0%
High Cholesterol	1.3%	1.8%	1.5%	1.4%	1.8%	1.5%	1.5%	1.5%	1.3%	1.8%	0.0%	1.6%	1.0%	6.9%	0.0%	1.2%	0.0%
Depression	12.4%	11.5%	12.6%	11.7%	12.7%	11.6%	13.0%	12.6%	12.6%	12.1%	0.0%	12.0%	9.7%	8.6%	14.1%	12.7%	3.6%
High Risk	11.5%	10.6%	11.2%	10.5%	11.9%	10.5%	12.2%	11.2%	11.6%	10.7%	0.0%	10.7%	8.1%	6.9%	12.5%	11.2%	2.1%
Age 19-34 years	11.6%	11.2%	11.7%	11.2%	12.3%	11.4%	10.9%	10.2%	11.8%	11.0%	0.0%	9.8%	8.3%	7.0%	12.7%	11.6%	6.2%
Age 34-49 years	12.2%	11.4%	12.1%	11.6%	12.8%	11.6%	12.0%	11.5%	12.5%	12.0%	0.0%	10.2%	8.6%	7.0%	12.7%	11.7%	2.0%
Age 49-64 years	11.9%	10.7%	11.9%	11.1%	11.4%	10.0%	11.8%	10.8%	11.6%	11.0%	0.0%	10.0%	8.3%	6.8%	10.7%	10.7%	2.5%

Table A5. Summary of self-reported reasons for not applying to OHP or denial of their application for the people in our sample who returned their initial mail survey and received an OHP application form.

Reason for:	Selected in lottery (N=2,223)			Not selected in lottery (N=443)		
	White Frequency (% of Total)	Non-white race Frequency (% of Total)[9]	Total frequency (% of non-missing)[10]	White Frequency (% of Total)	Non-white race Frequency (% of Total)	Total frequency (% of non-missing)
**Denial of application**						
High income or assets	198 (80.2)	49 (19.8)	247 (16.6)	19 (82.6)	4 (17.4)	23 (7.5)
Had not been uninsured long enough to qualify for coverage	17 (77.3)	5 (22.7)	22 (1.48)	1 (33.3)	2 (66.7)	3 (1.0)
Could not send paperwork to prove citizenship	20 (69.0)	9 (31.0)	29 (1.95)	0 (0.0)	4 (100.0)	4 (1.3)
Late turning the application in	7 (70.0)	3 (30.0)	10 (0.7)	0 (0.0)	1 (100.0)	1 (1.3)
Could not send other required paperwork	8 (53.3)	7 (46.7)	15 (1.0)	3 (60.0)	2 (40.0)	5 (1.6)
Other reasons	62 (65.3)	33 (34.7)	95 (6.4)	36 (78.3)	10 (21.7)	46 (15.0)
Unknown Reason	35 (55.5)	28 (44.5)	63 (4.2)	47 (63.5)	27 (36.5)	74 (24.2)
Non-Missing	1,092 (73.5)	394 (26.5)	1,486	205 (67.0)	101 (33.0)	306
Missing	547 (74.2)	190 (25.8)	737	85 (62.0)	52 (38.0)	137
**Decision not to apply**						
High income or assets	61 (87.1)	9 (12.9)	70 (11.1)	7 (70.0)	3 (30.0)	10 (9.6)
Found another insurance	65 (87.2)	9 (12.8)	74 (11.8)	6 (75.0)	2 (25.0)	8 (7.7)
Did not finish the application	185 (72.8)	69 (27.2)	254 (40.4)	14 (48.3)	15 (51.7)	29 (27.9)
Decided not to apply	47 (79.7)	12 (20.3)	59 (9.4)	4 (80.0)	1 (20.0)	5 (4.8)
The paper work is hassle	44 (86.3)	7 (13.7)	51 (8.1)	9 (81.8)	2 (18.2)	11 (10.6)
Could not find paperwork to prove citizenship	28 (82.4)	6 (17.6)	34 (5.4)	2 (100.0)	0 (0.0)	2 (1.9)
Could not find other required paperwork (e.g. income proof)	34 (75.5)	11 (24.5)	45 (7.2)	10 (83.3)	2 (16.7)	12 (11.5)
Did not return OHP application for some Other reason	158 (74.5)	54 (25.5)	212 (33.8)	27 (64.3)	15 (35.7)	42 (40.4)
Do not know why did not return the application	7 (63.6)	4 (36.4)	11 (1.8)	2 (33.3)	4 (66.7)	6 (5.8)
Non-missing	475 (75.6)	153 (24.4)	628	62 (59.6)	42 (40.4)	104
Missing	1,164 (73.0)	431 (27.0)	1,595	228 (67.3)	111 (32.7)	339

9 The total frequency sample consists of individuals who responded with Yes to the specified reason.

10 The non-missing sample for each set of reasons which is used to calculate the percentages consists of individuals who responded with either Yes or No to at least one of the reasons.

References

1. Baicker K, Taubman SL, Allen HL, Bernstein M, Gruber JH, Newhouse JP, et al. The Oregon Experiment — Effects of Medicaid on Clinical Outcomes. N Engl J Med. 2013 May 2;368(18):1713–22.
