# Supplementary material for: Heterogeneity within the Oregon Health Insurance Experiment: An application of causal forests
Source: PLoS One. 2024 Jan 18;19(1):e0297205. doi: 10.1371/journal.pone.0297205 (PMC10796043; doi:10.1371/journal.pone.0297205)
Supplement: S3 File — Test calibration for intent-to-treat analysis. (PDF) [file pone.0297205.s003.pdf]

Supplement Appendix:

S2. Omnibus Tests for Heterogeneity:

S2.1. Test Calibration for intent-to-treat analysis:

This test computes the best linear fit of the lottery effects using the out-of-bag predictions from the fitted causal forest and the mean forest prediction [1]. Table A6 below shows two statistics: mean forest prediction and differential forest prediction and the p-values of testing whether the value is greater than zero (P-value 0) and different from one (P-value 1) respectively. The results suggest the absence of heterogeneity the differential forest prediction statistics are not significantly greater than zero except for the OHP uptake outcome where the p-value is less than 0.001 and the predicted heterogeneity is calibrated as well since the statistic is close to 1.

Table A6. Calibration test for intent-to-treat analysis

Outcome		Mean forest prediction	Differential forest prediction
**Mental component score**	Estimate	0.999	-9.814
	SE.	0.393	2.906
	P-value 0	0.006	0.999
	P-value 1	0.797	0.155
**Physical component score**	Estimate	1.0048	-4.931
	SE.	0.563	2.23
	P-value 0	0.04	0.99
	P-value 1	0.798	0.167
**Amount of out-of-pocket spending**	Estimate	0.996	-1.053
	SE.	0.324	1.36
	P-value 0	0.001	0.78
	P-value 1	0.797	0.009
**Prescription Drugs**	Estimate	0.997	-43.89
	SE.	0.241	5.54
	P-value 0	<0.001	1.00
	P-value 1	0.786	0.015
**Office visits**	Estimate	1.006	1.449
	SE.	0.374	1.508
	P-value 0	0.004	0.168
	P-value 1	0.797	0.735
**Hospital admissions**	Estimate	1.119	-4333
	SE.	0.979	160
	P-value 0	0.127	1.00
	P-value 1	0.77	<0.001

**Outpatient surgery visits**	Estimate	0.975	-13.45
	SE.	1.77	4.13
	P-value 0	0.29	0.999
	P-value 1	0.797	0.208
**Emergency department visits**	Estimate	1.045	-8.44
	SE.	5.09	2.67
	P-value 0	0.419	0.999
	P-value 1	0.798	0.006
**OHP - Uptake**	Estimate	0.999	1.08
	SE.	0.024	0.072
	P-value 0	<0.001	<0.001
	P-value 1	0.798	0.189

References

1. Athey S, Wager S. Estimating Treatment Effects with Causal Forests: An Application. ArXiv190207409 Stat [Internet]. 2019 Feb 20 [cited 2022 Feb 4]; Available from: http://arxiv.org/abs/1902.07409
