# Supplementary material for: Heterogeneity within the Oregon Health Insurance Experiment: An application of causal forests
Source: PLoS One. 2024 Jan 18;19(1):e0297205. doi: 10.1371/journal.pone.0297205 (PMC10796043; doi:10.1371/journal.pone.0297205)
Supplement: S4 File — Rank-Weighted Average Treatment Effect (RATE) for insurance-effect analysis. (PDF) [file pone.0297205.s004.pdf]

# Supplement Appendix:

## S2. Omnibus Tests for Heterogeneity:

### S2.2. Rank-Weighted Average Treatment Effect (RATE) for Insurance-effect analysis:

Since the calibration test is not applicable for the instrumental forest, we use the Rank-Weighted Average Treatment Effect (RATE) metric suggested by Yadlowsky et al [1] to evaluate whether there is any noteworthy heterogeneity present, and how good our CATE estimates are at distinguishing subpopulations with different treatment effects. That is, how much benefit there is to prioritizing insurance provision based on the heterogeneity that is obtained by our instrumental forest. This approach gives, based on the estimated CATEs, a high score to individuals estimated to benefit more from insurance and a low score to those with lower benefit. The benefit is the expected increase in outcomes from providing insurance to a fraction of the population with the largest prioritization scores as opposed to giving treatment to a randomly selected fraction of the same size.

The figures A11 & A12 show the Target Operator Characteristic (TOC) curves on the outcomes. These curves chop the population up into groups defined by above mentioned scores, then plot this over all groups where each group is the top $q$-th fraction of individuals with the largest score, and Table A7 represents the areas under these curves (Receiver Operating Characteristic(ROC)) which act as indicators of heterogeneity. Table A7 shows that all of the ROC statistics are either negative or statistically insignificant which is an indicator of the absence of heterogeneity, except for the OHP uptake where it is positive and statistically significant.

**Table A7. RATE estimates and standard errors**

| Outcome | Mental component score | | Physical component score | | Amount of out-of-pocket spending | | No. prescription drugs | | No. office visits | | Hospital admissions | | Outpatient surgery | | Emergency department visits | | OHP uptake | |
|---|---|---|---|---|---|---|---|---|---|---|---|---|---|---|---|---|---|---|
| | RATE | Error | RATE | Error | RATE | Error | RATE | Error | RATE | Error | RATE | Error | RATE | Error | RATE | Error | RATE | Error |
| | -3.50 | 3.6 | -3.14 | 0.97 | -258.34 | 134.9 | -0.43 | 0.22 | -0.27 | 1.00 | -0.38 | 0.073 | -0.044 | 0.04 | -0.55 | 0.18 | 0.042 | 0.007 |

**Figure A11. Targeting Operator Characteristic curve evaluated on mental component score, physical component score, amount of out-of-pocket spending, and number of prescription drugs from health insurance**

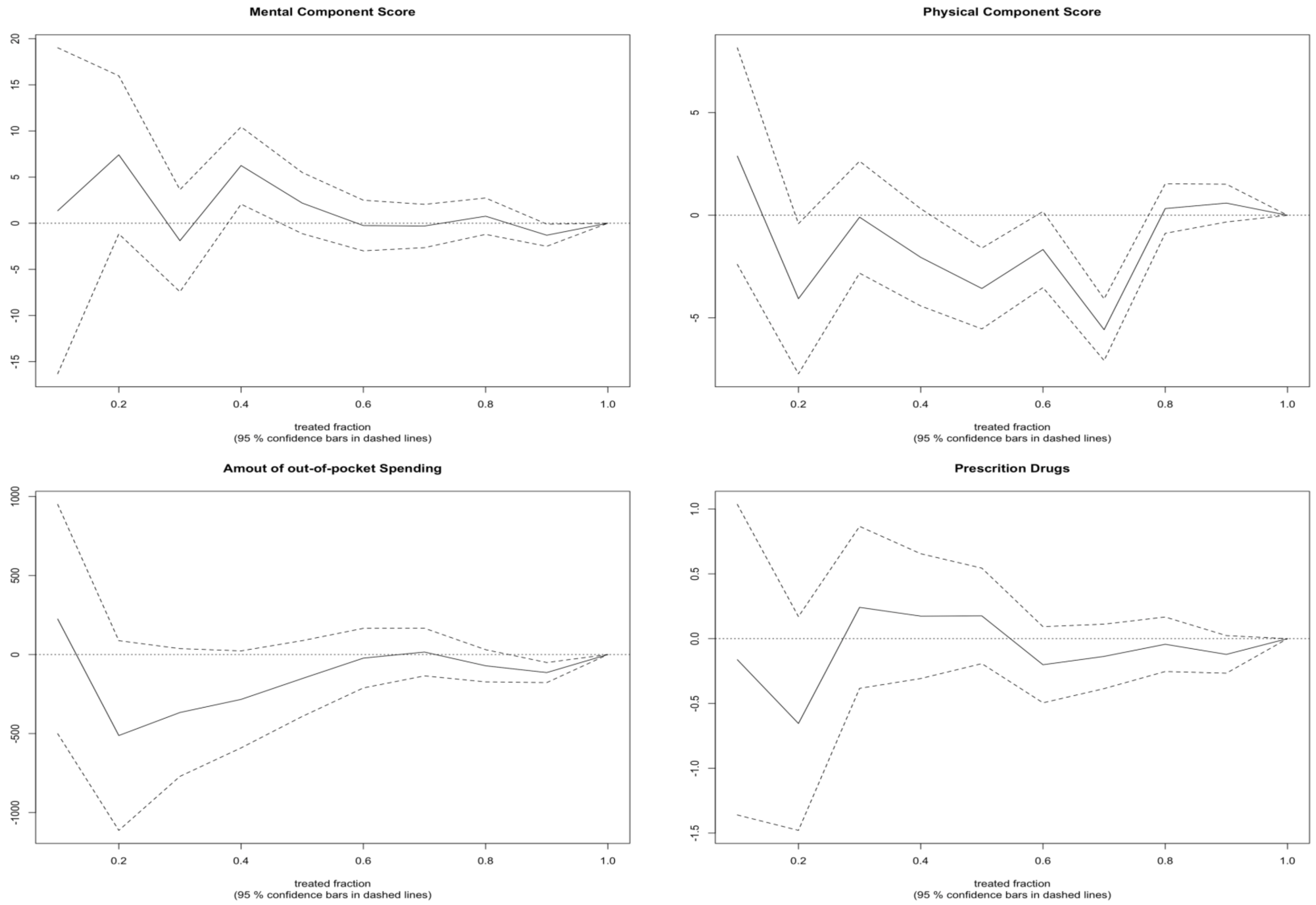

**Figure A12. Targeting Operator Characteristic curve evaluated on number of office visits, hospital admissions, outpatient surgery visits, and emergency department visits from health insurance.**

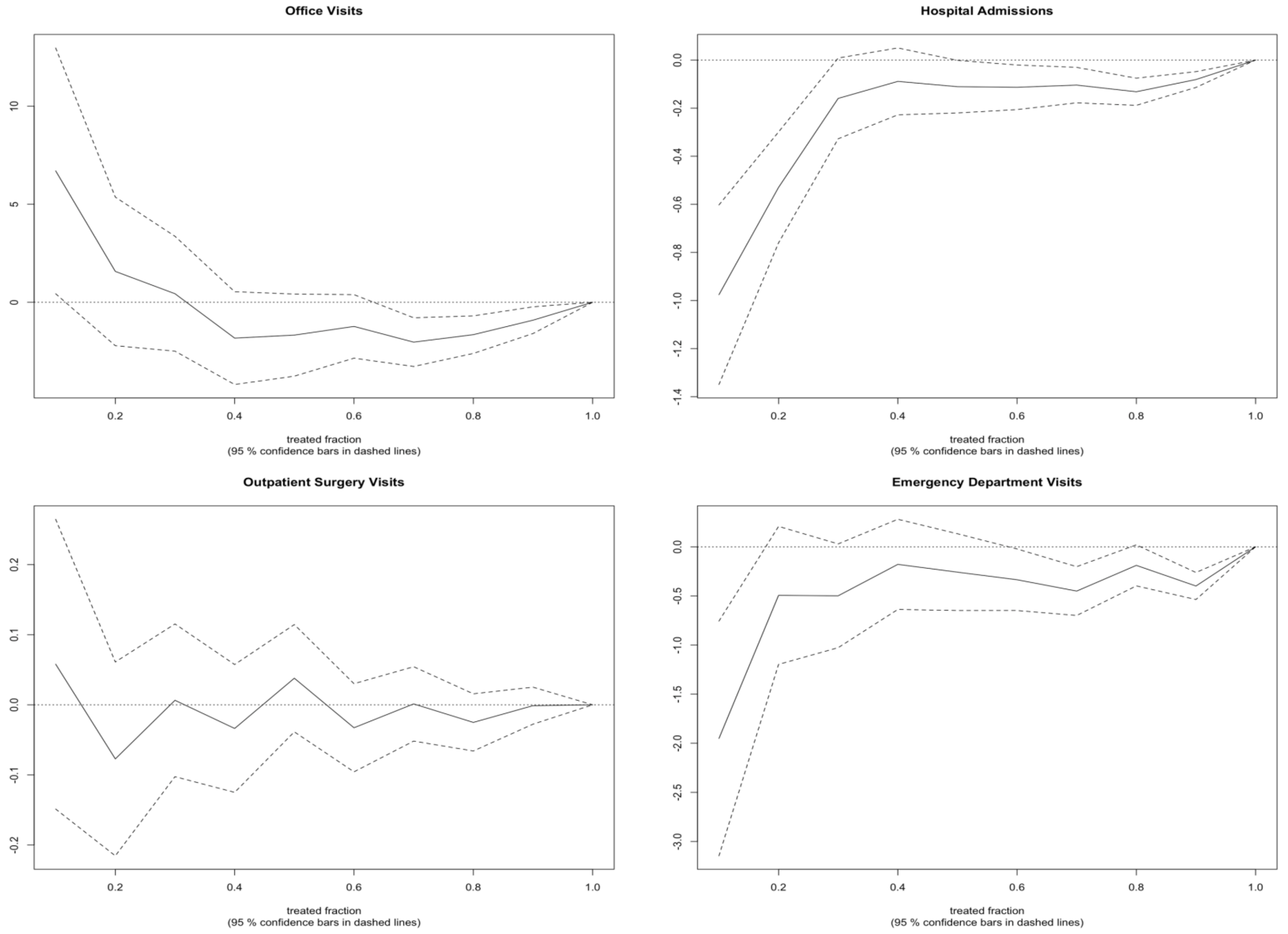

# References

1. Yadlowsky S, Fleming S, Shah N, Brunskill E, Wager S. Evaluating Treatment Prioritization Rules via Rank-Weighted Average Treatment Effects. ArXiv211107966 Stat [Internet]. 2021 Nov 15 [cited 2022 Apr 22]; Available from: http://arxiv.org/abs/2111.07966
