# Supplementary material for: Heterogeneity within the Oregon Health Insurance Experiment: An application of causal forests
Source: PLoS One. 2024 Jan 18;19(1):e0297205. doi: 10.1371/journal.pone.0297205 (PMC10796043; doi:10.1371/journal.pone.0297205)

Supplement Appendix:

S3. Robustness of the results:

S3.1. Analysis of larger dataset.

To assess the sensitivity of our findings, we analyze data for all 23,741 respondents to the mail survey and compared the findings with the in-person-interviews dataset consisting of 12,229 observations. However, it should be noted that the mail survey data excluded the pre-lottery diagnosis covariates, and that data for the following outcomes were unavailable in this dataset: physical & mental component scores and outpatient surgery visits. Moreover, the time-horizon is different among the two samples, 12 months in the mail survey data versus 24 months in the in-person data, thus effects tend to be larger for the latter analysis. In general, the results are similar.

For OHP uptake, the most notable difference is that effects on uptake for individuals aged 49 to 64 appear more similar to the effects for those aged 34 to 49 (Figure A13). Turning to the effects if HI, on the amount of out-of-pocket spending, we obtain narrower confidence intervals using larger the mail survey dataset as we might expect. Although we find more evidence of heterogeneity of insurance effects particularly in high-risk subgroups (Figure A14), we cannot be confident that this is not driven by the inability to control for baseline comorbidities in that analysis. Similar comments apply to the other outcomes, which we do not present here, however results for these outcomes are available upon request.

Figure A13. Forest plot for subgroups' conditional average treatment effects of lottery selection on OHP uptake using in-person and mail survey datasets.

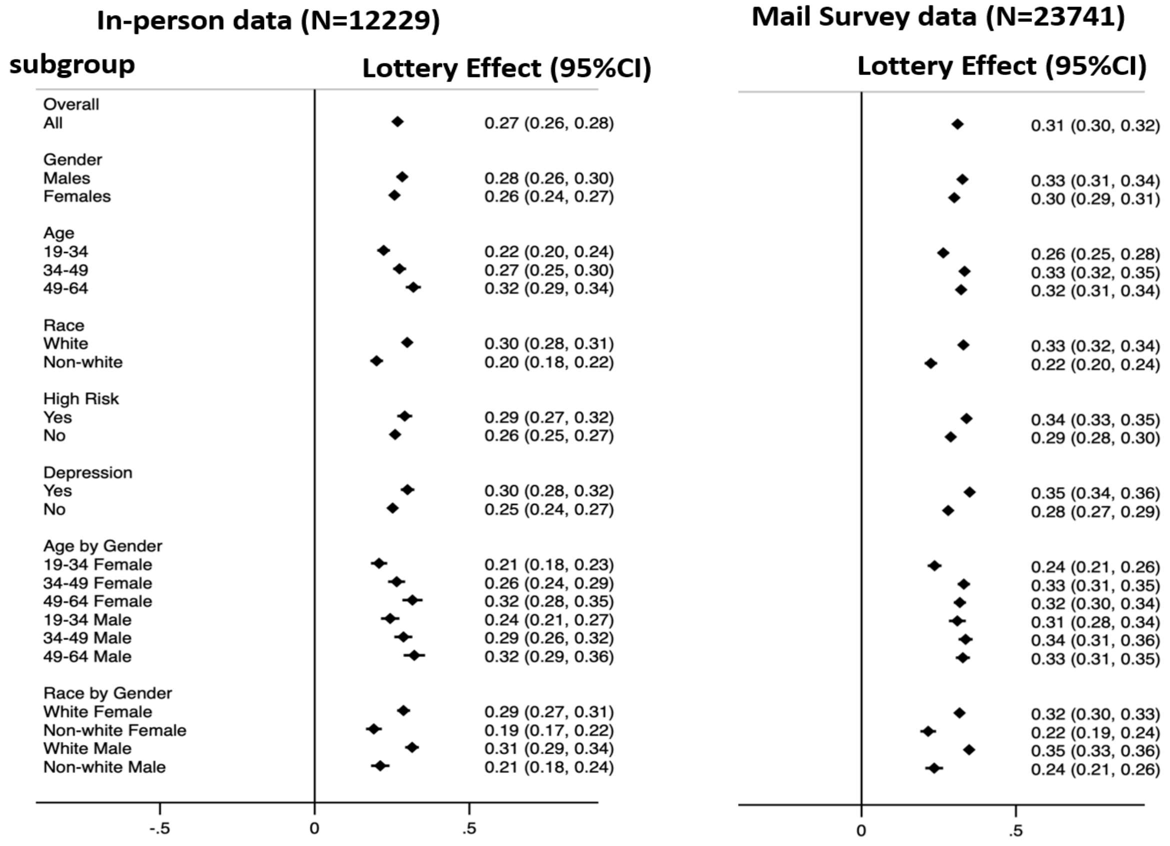

Figure A14. Forest plot for subgroups' conditional average treatment effects of insurance on amount of out-of-pocket spending using in-person and mail survey datasets.

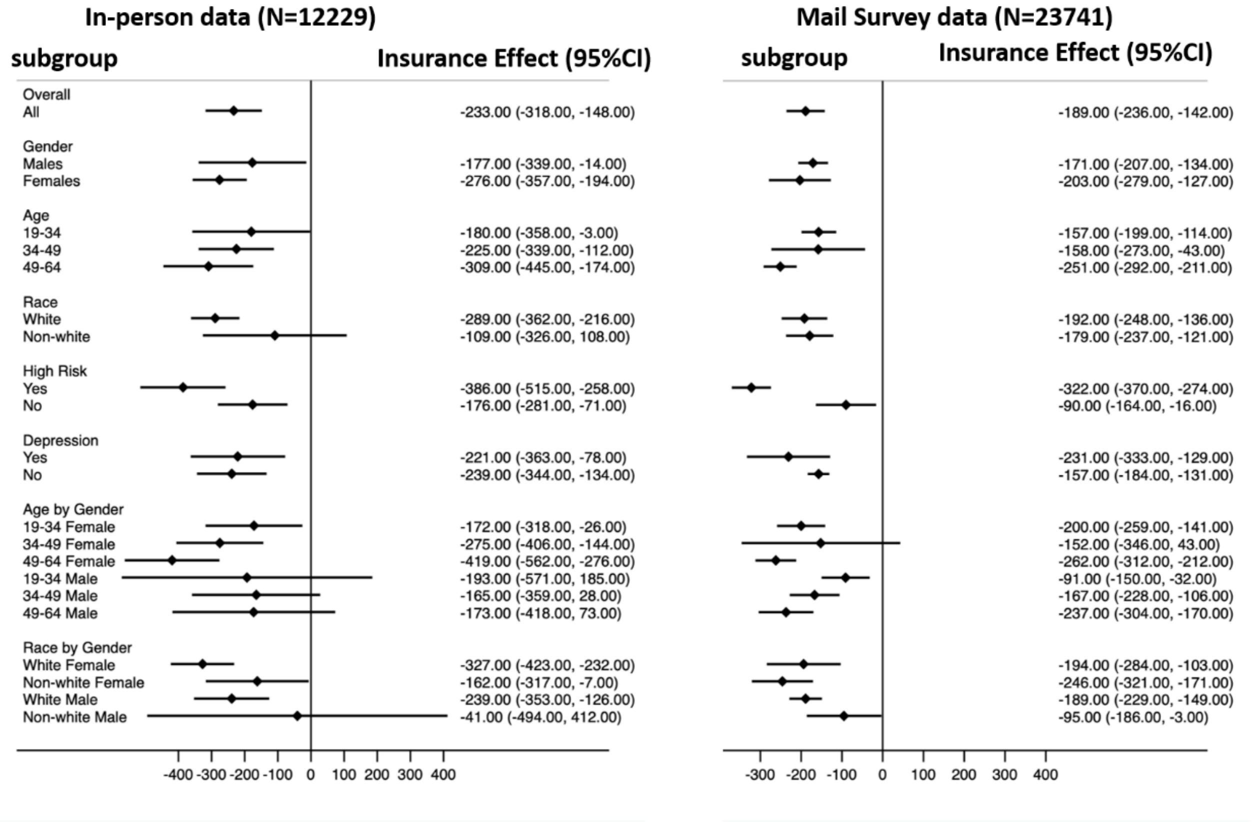

Supplement: S5 File — (PDF) [file pone.0297205.s005.pdf]
