# Supplementary material for: Heterogeneity within the Oregon Health Insurance Experiment: An application of causal forests
Source: PLoS One. 2024 Jan 18;19(1):e0297205. doi: 10.1371/journal.pone.0297205 (PMC10796043; doi:10.1371/journal.pone.0297205)
Supplement: S6 File — Analysis lottery draws. (PDF) [file pone.0297205.s006.pdf]

Supplement Appendix:

S3. Robustness of the results:

S3.2. Analysis lottery draws.

Eight lottery draws occurred in 2008, with two in March and one in each following month through August. Figure A15 shows a heavy concentration of enrollment in the first draw, followed by a slight decrease in enrollment rate over the subsequent draws. While this could be an indication of a censoring effect, Figure A16 demonstrates that baseline characteristics do not differ significantly across the different draws, suggesting there is no evidence of a censoring effect.

Figure A15.Enrollmet rate over the eight lottery draws.

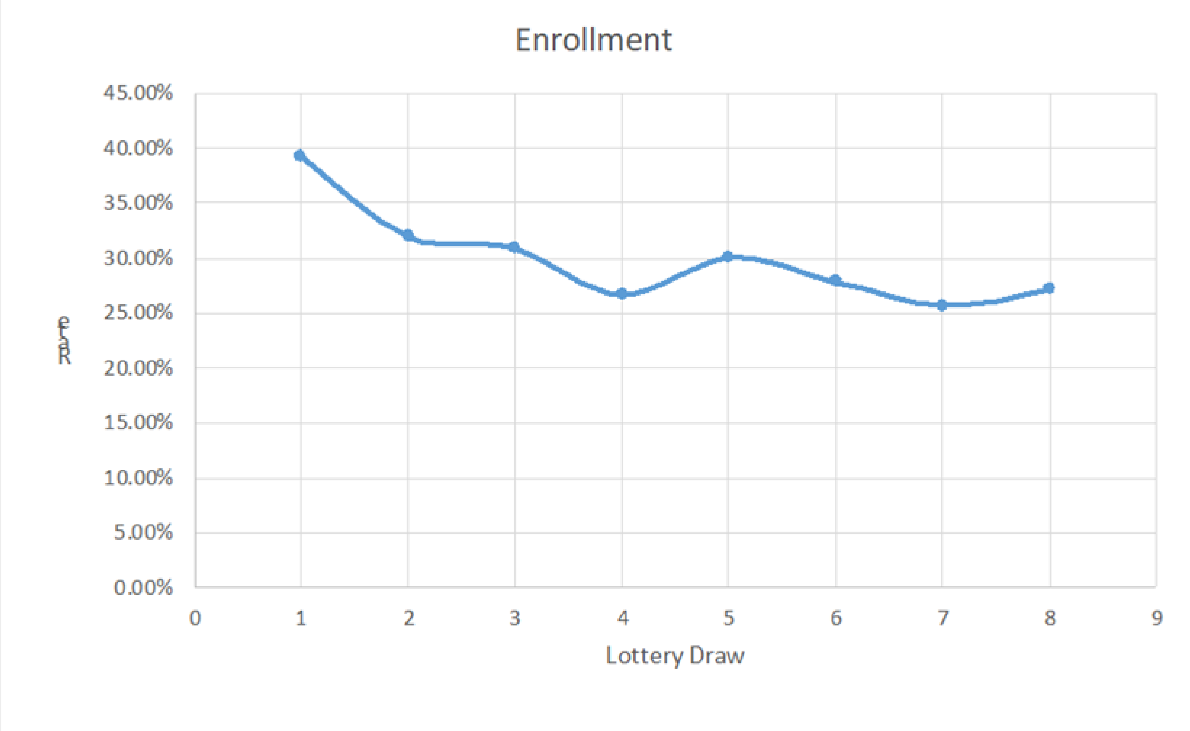

Figure A16. Distribution of baseline characteristics over the eight lottery draws.

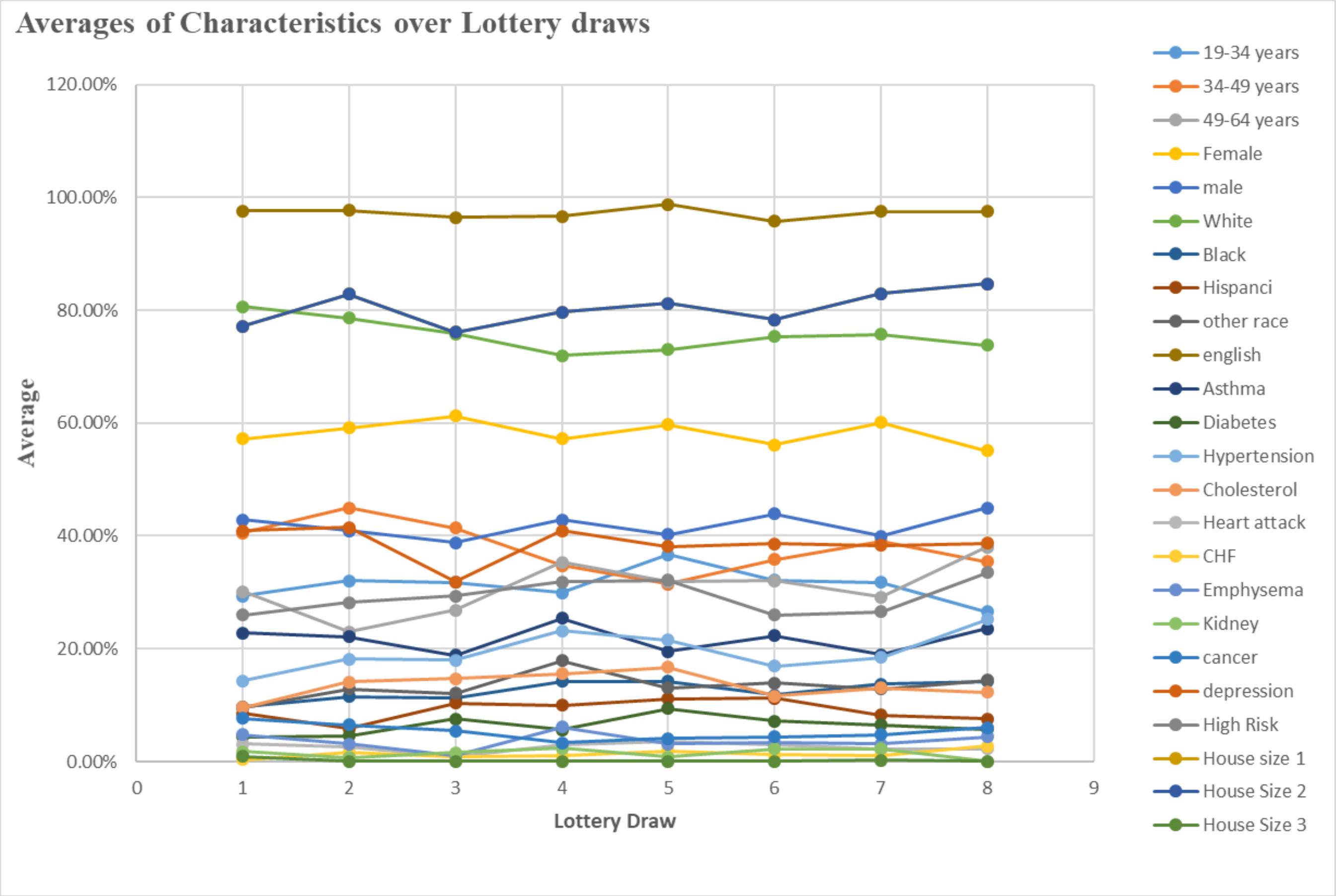

References

Athey, Susan, Julie Tibshirani, and Stefan Wager. 2018. “Generalized Random Forests.” *ArXiv:1610.01271 [Econ, Stat]*, April. http://arxiv.org/abs/1610.01271.

Athey, Susan, and Stefan Wager. 2019. “Estimating Treatment Effects with Causal Forests: An Application.” *ArXiv:1902.07409 [Stat]*, February. http://arxiv.org/abs/1902.07409.

Baicker, Katherine, Sarah L. Taubman, Heidi L. Allen, Mira Bernstein, Jonathan H. Gruber, Joseph P. Newhouse, Eric C. Schneider, Bill J. Wright, Alan M. Zaslavsky, and Amy N. Finkelstein. 2013. “The Oregon Experiment — Effects of Medicaid on Clinical Outcomes.” *New England Journal of Medicine* 368 (18): 1713–22. https://doi.org/10.1056/NEJMsa1212321.

Bargagli Stoffi, Falco J., and Giorgio Gnecco. 2020. “Causal Tree with Instrumental Variable: An Extension of the Causal Tree Framework to Irregular Assignment Mechanisms.” *International Journal of Data Science and Analytics* 9 (3): 315–37. https://doi.org/10.1007/s41060-019-00187-z.

Chen, Jau-er, and Chen-Wei Hsiang. 2019. “Causal Random Forests Model Using Instrumental Variable Quantile Regression.” *Econometrics* 7 (4): 49. https://doi.org/10.3390/econometrics7040049.

Finkelstein, Amy, Sarah Taubman, Bill Wright, Mira Bernstein, Jonathan Gruber, Joseph P. Newhouse, Heidi Allen, Katherine Baicker, and Oregon Health Study Group. 2012. “The Oregon Health Insurance Experiment: Evidence from the First Year*.” *The Quarterly Journal of Economics* 127 (3): 1057–1106. https://doi.org/10.1093/qje/qjs020.

Johnson, Michael, Jiongyi Cao, and Hyunseung Kang. 2021. “Detecting Heterogeneous Treatment Effect with Instrumental Variables.” *ArXiv:1908.03652 [Stat]*, January. http://arxiv.org/abs/1908.03652.

Wang, Guihua, Jun Li, and Wallace J. Hopp. 2021. “An Instrumental Variable Forest Approach for Detecting Heterogeneous Treatment Effects in Observational Studies.” *Management Science*, September, mnsc.2021.4084. https://doi.org/10.1287/mnsc.2021.4084.

Yadlowsky, Steve, Scott Fleming, Nigam Shah, Emma Brunskill, and Stefan Wager. 2021. “Evaluating Treatment Prioritization Rules via Rank-Weighted Average Treatment Effects.” *ArXiv:2111.07966 [Stat]*, November. http://arxiv.org/abs/2111.07966.
