# Supplementary figures and images for: Heterogeneity within the Oregon Health Insurance Experiment: An application of causal forests
Source: PLoS One. 2024 Jan 18;19(1):e0297205. doi: 10.1371/journal.pone.0297205 (PMC10796043; doi:10.1371/journal.pone.0297205)

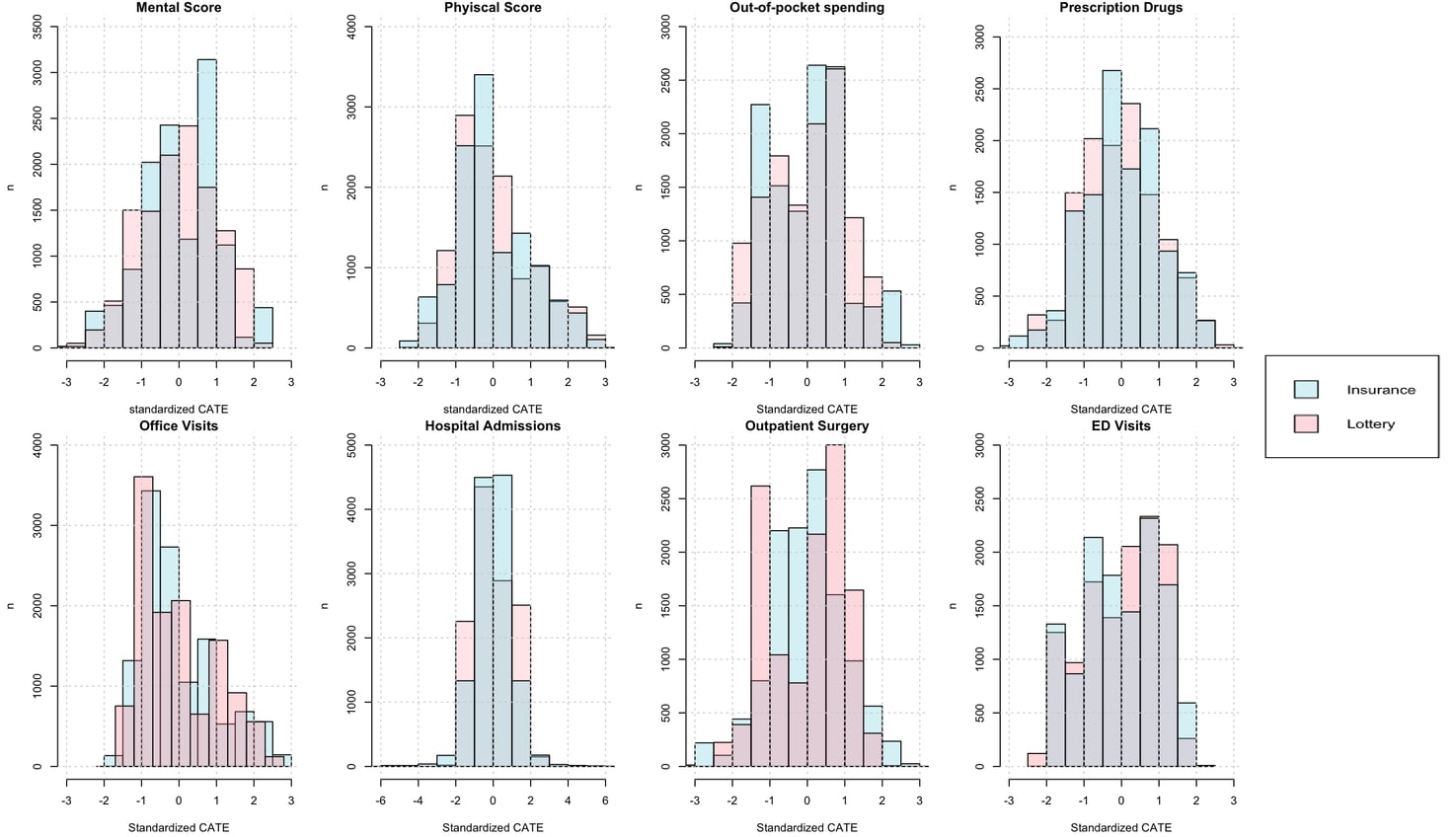

Supplement: S1 Fig — (TIF) [file pone.0297205.s007.tif]

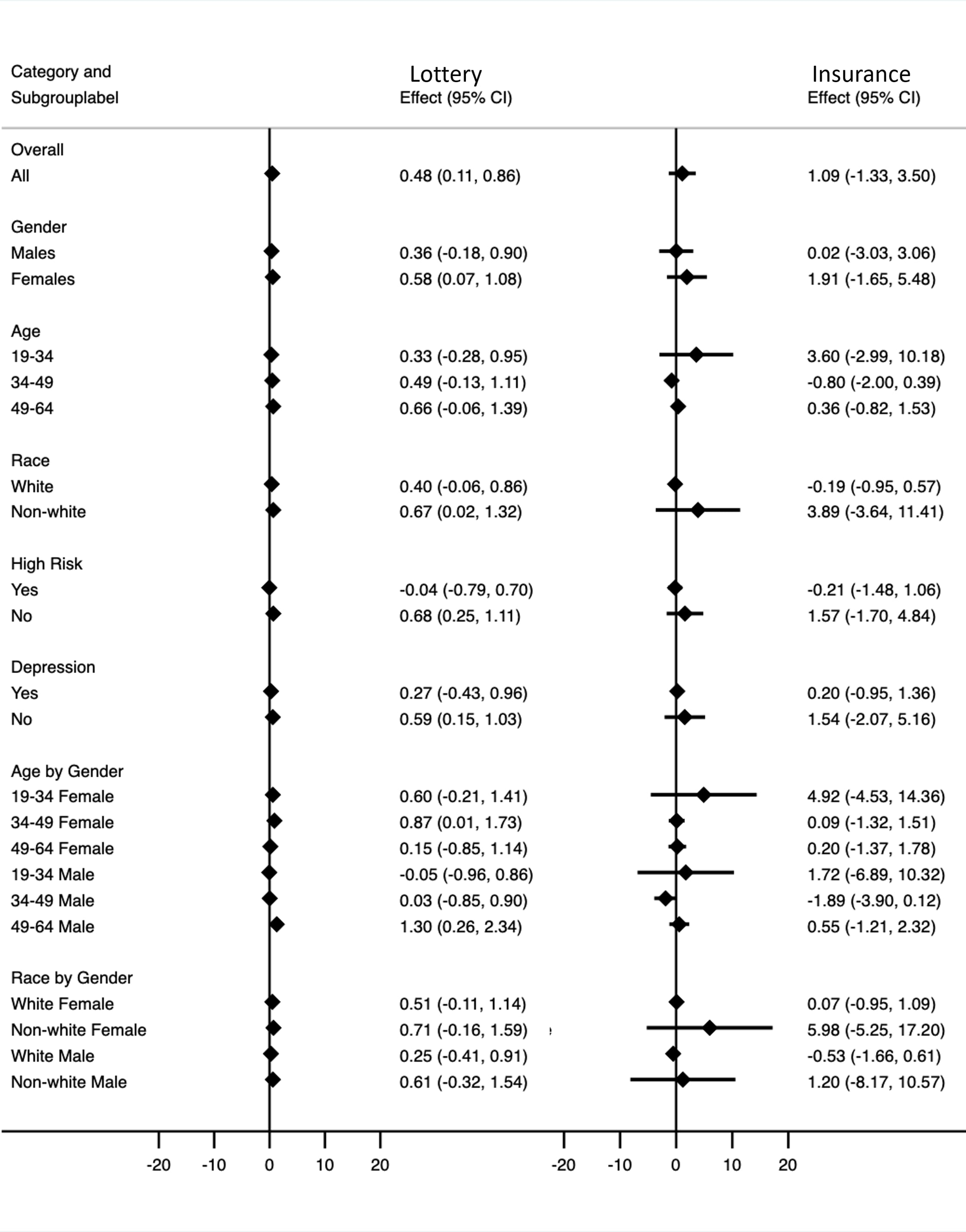

Supplement: S2 Fig — (TIF) [file pone.0297205.s008.tif]

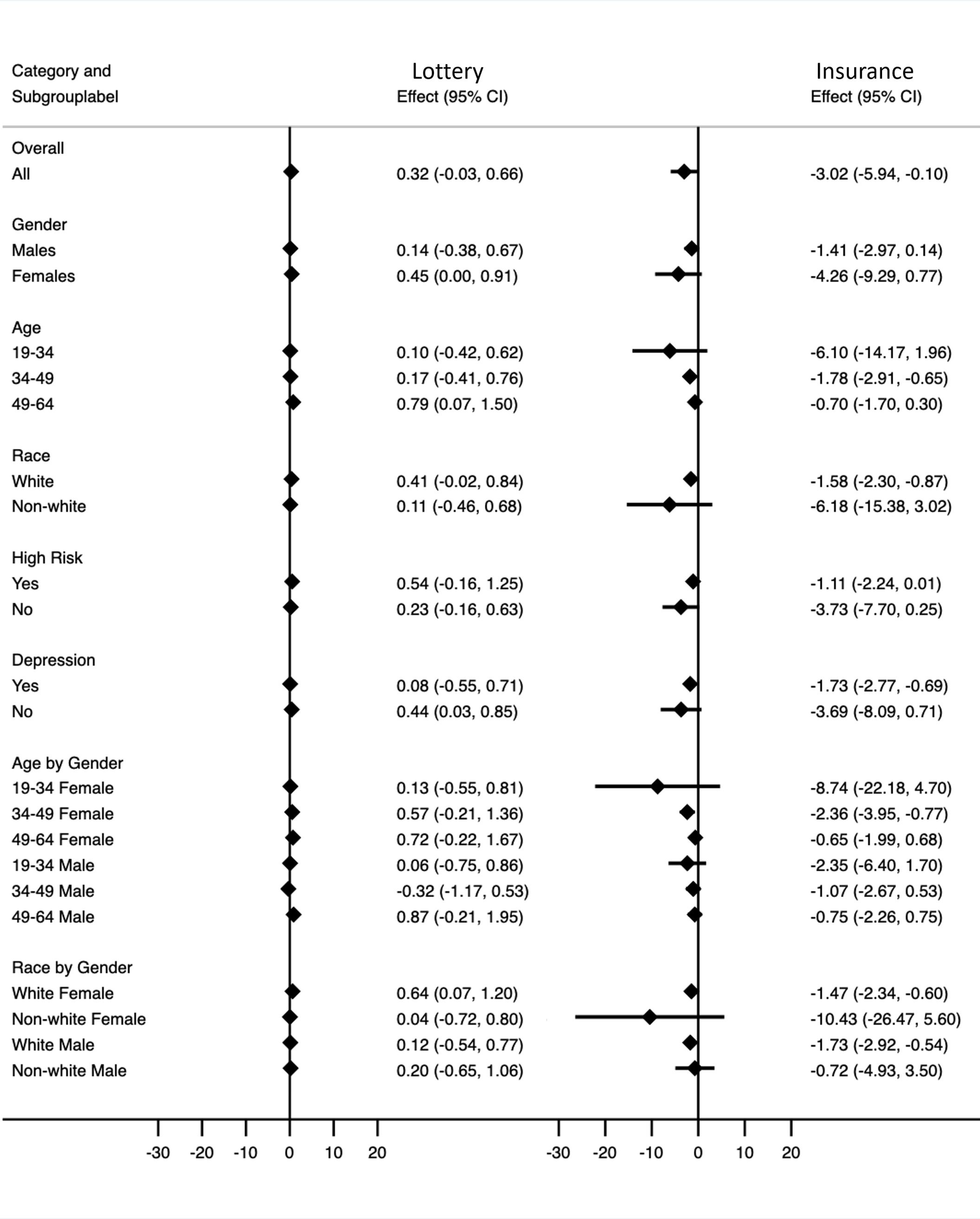

Supplement: S3 Fig — (TIF) [file pone.0297205.s009.tif]

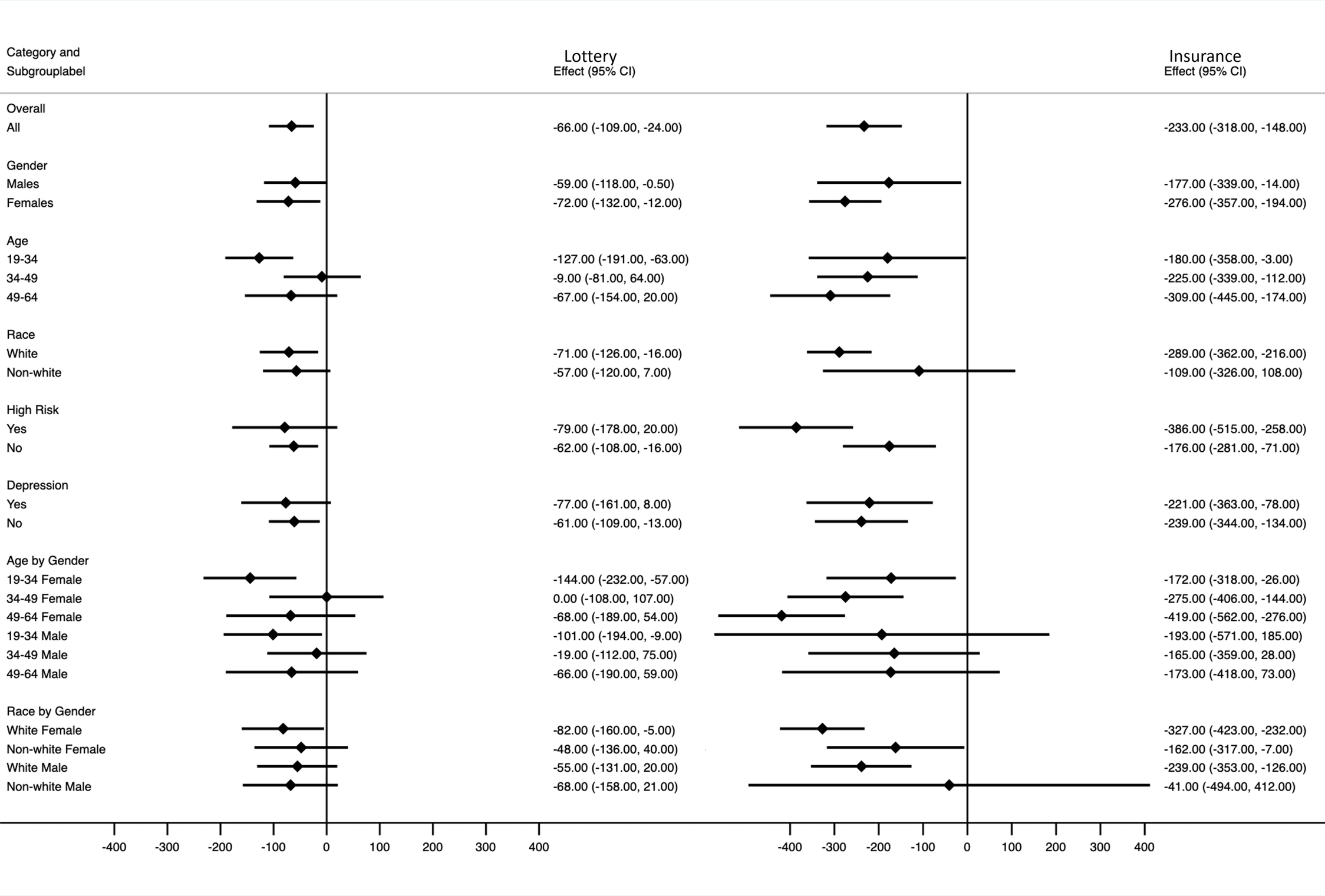

Supplement: S4 Fig — (TIF) [file pone.0297205.s010.tif]

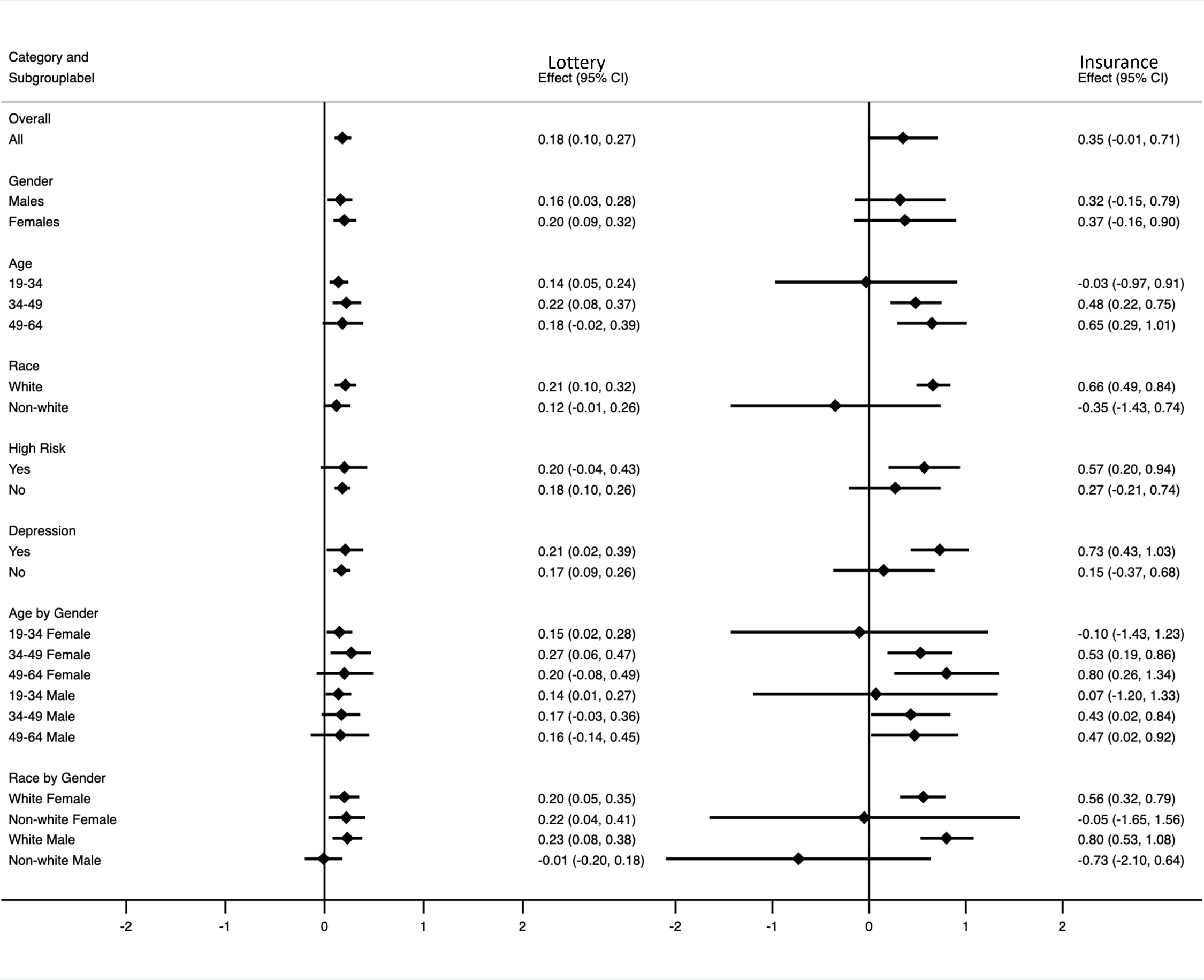

Supplement: S5 Fig — (TIF) [file pone.0297205.s011.tif]

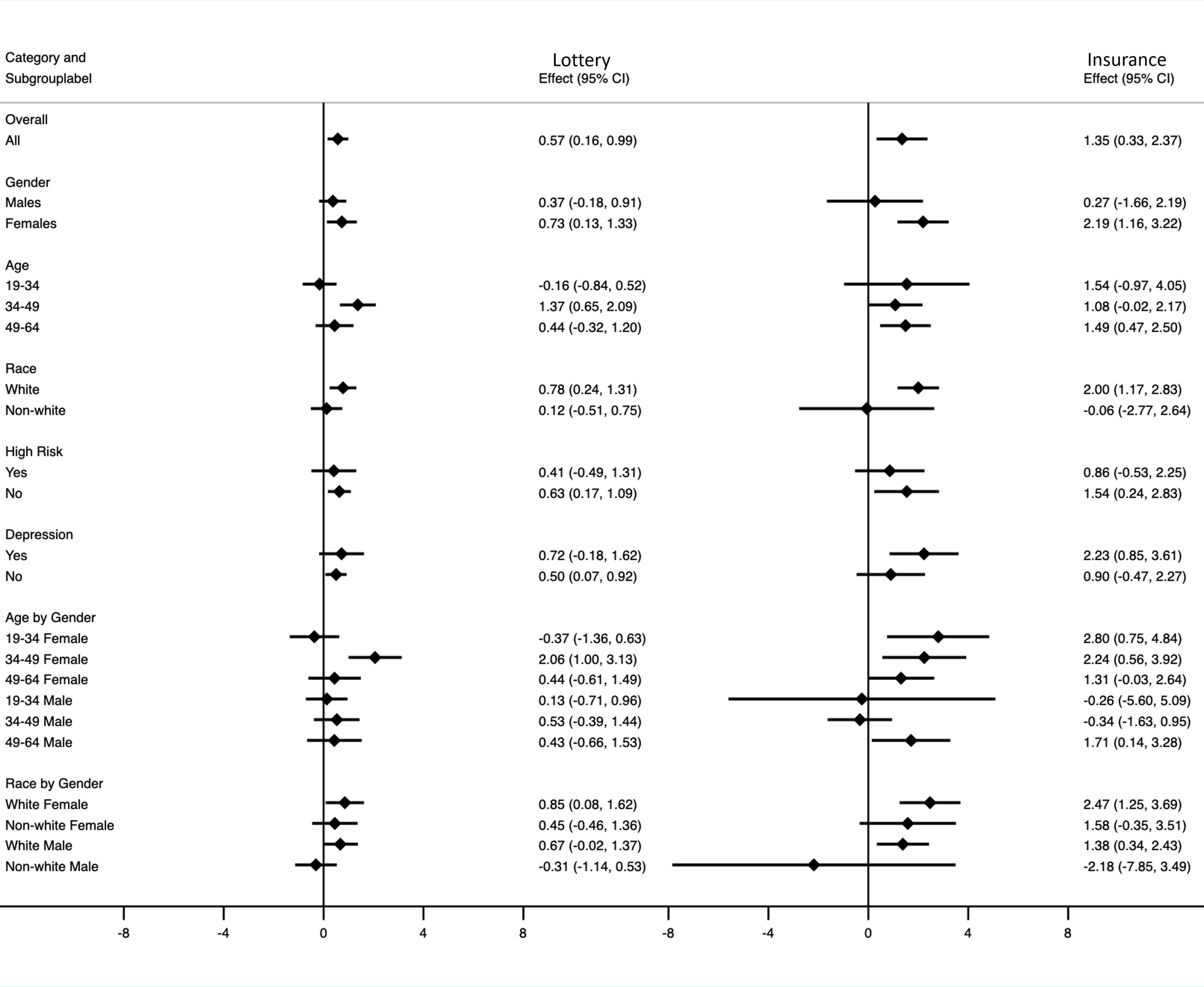

Supplement: S6 Fig — (TIF) [file pone.0297205.s012.tif]

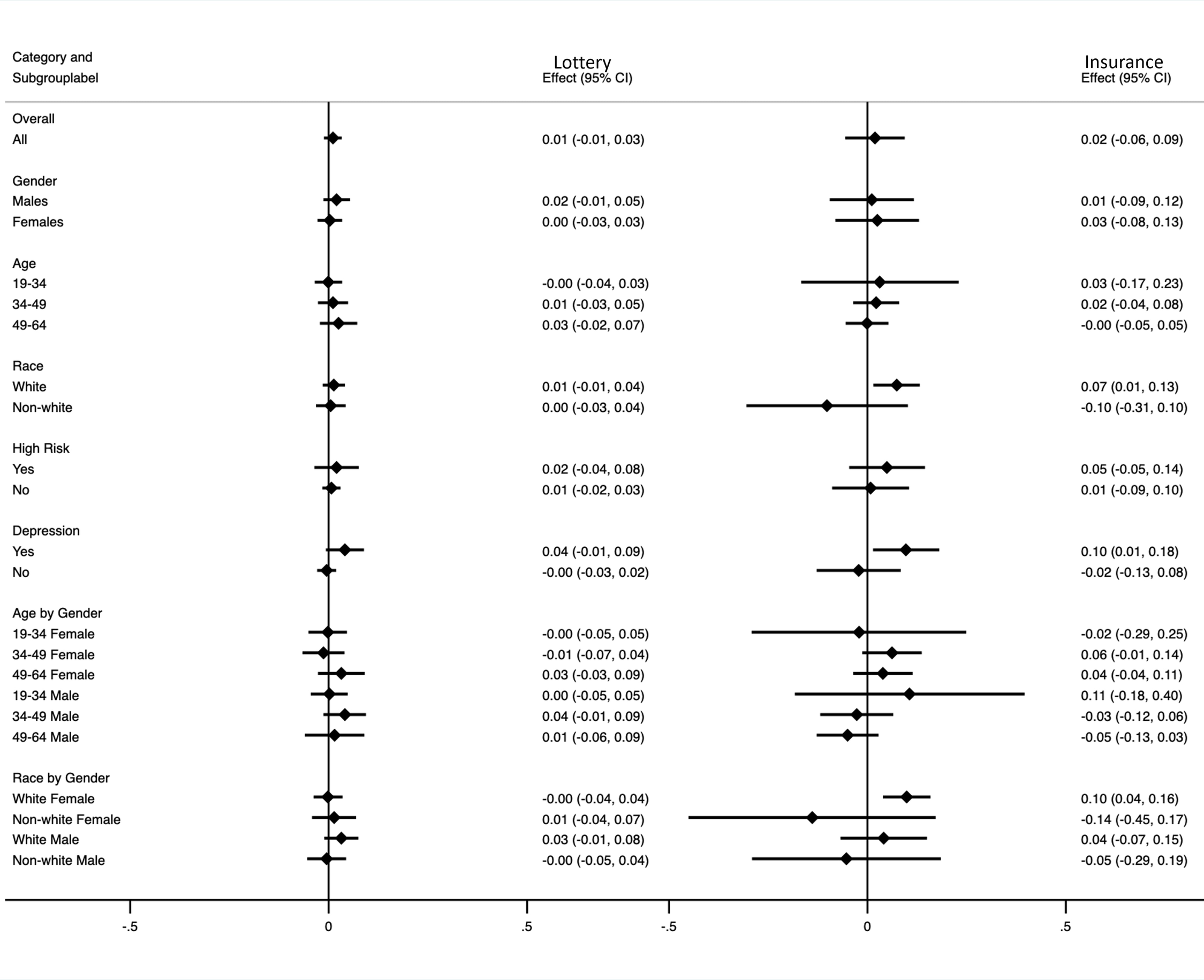

Supplement: S7 Fig — (TIF) [file pone.0297205.s013.tif]

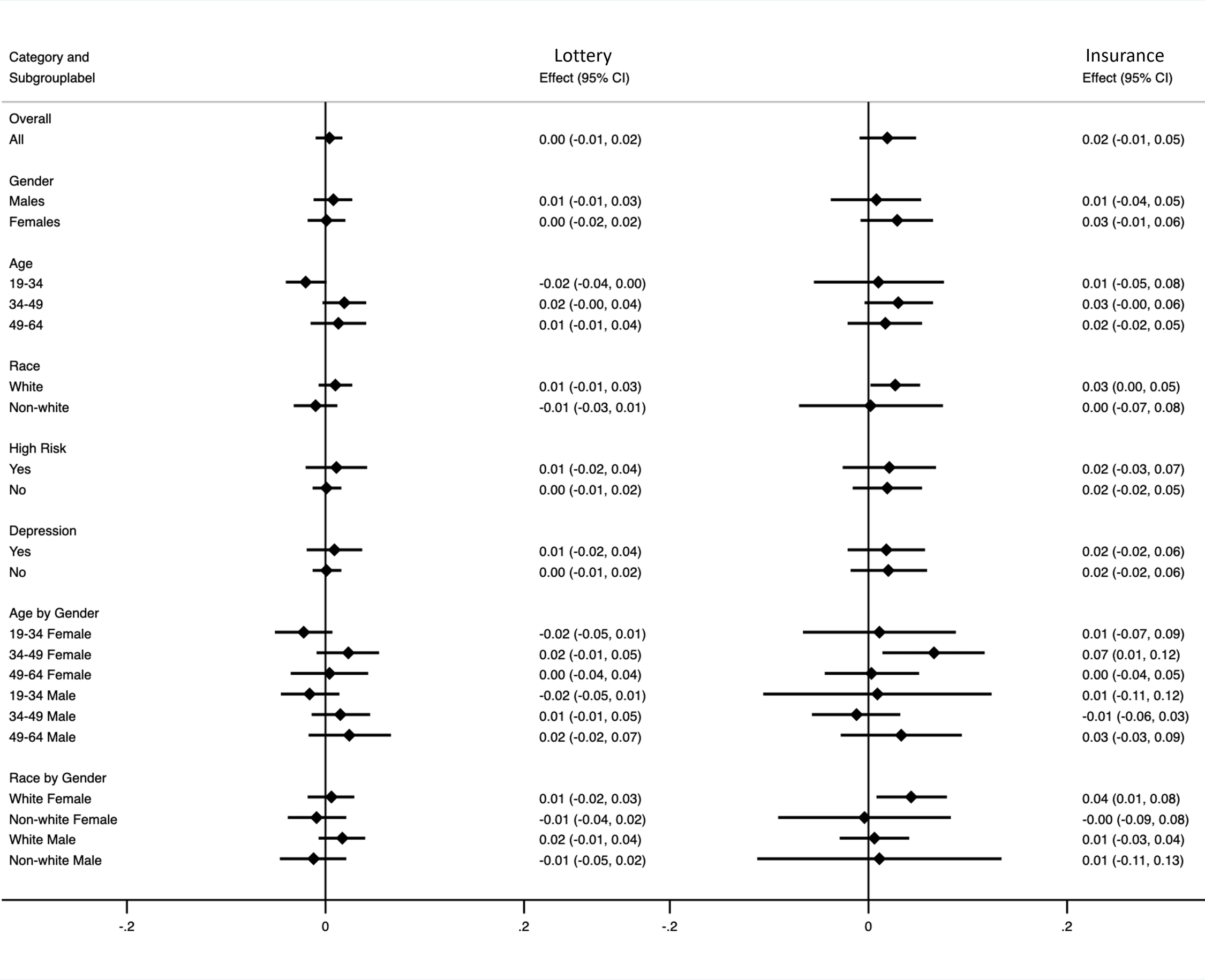

Supplement: S8 Fig — (TIF) [file pone.0297205.s014.tif]

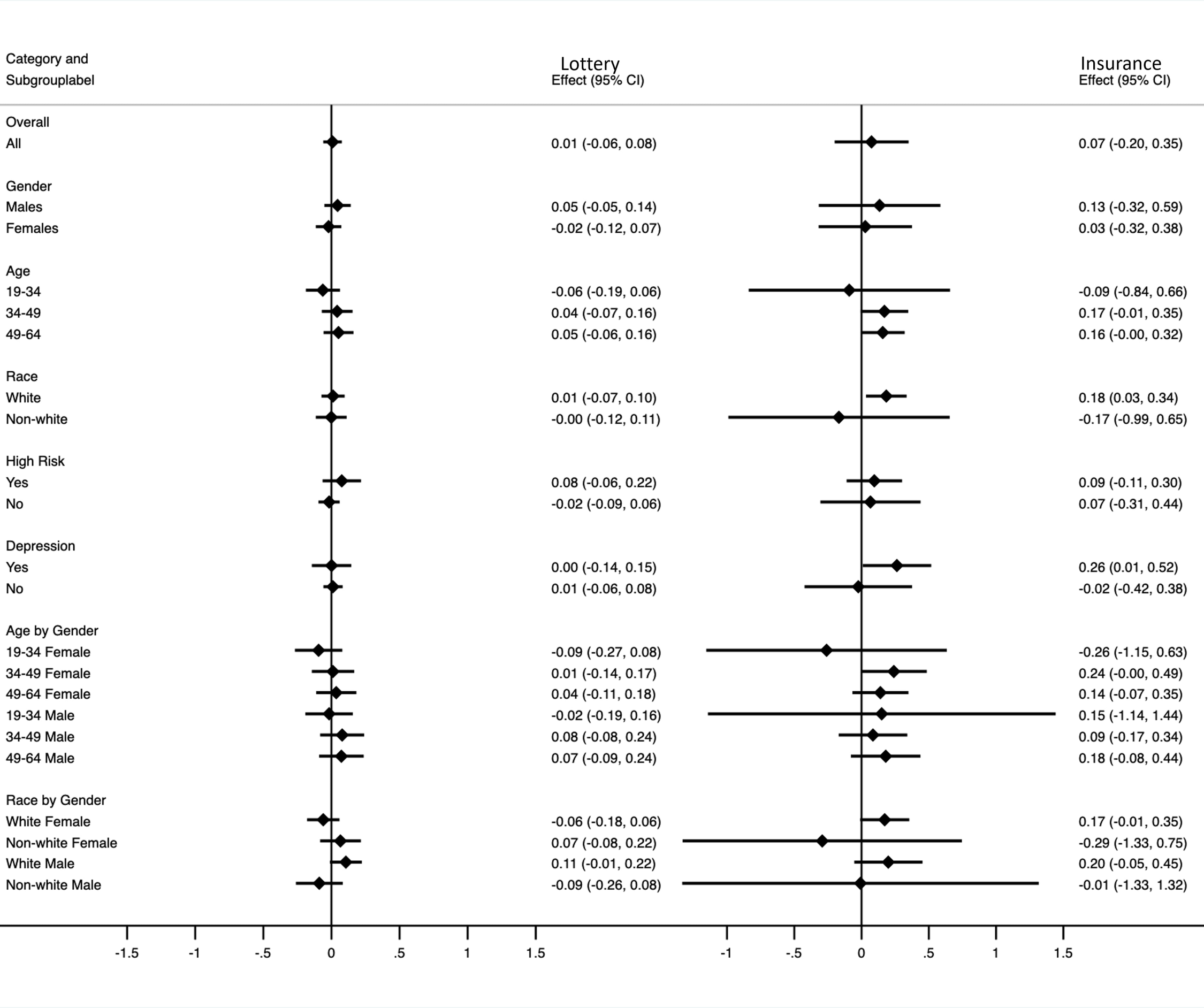

Supplement: S9 Fig — (TIF) [file pone.0297205.s015.tif]

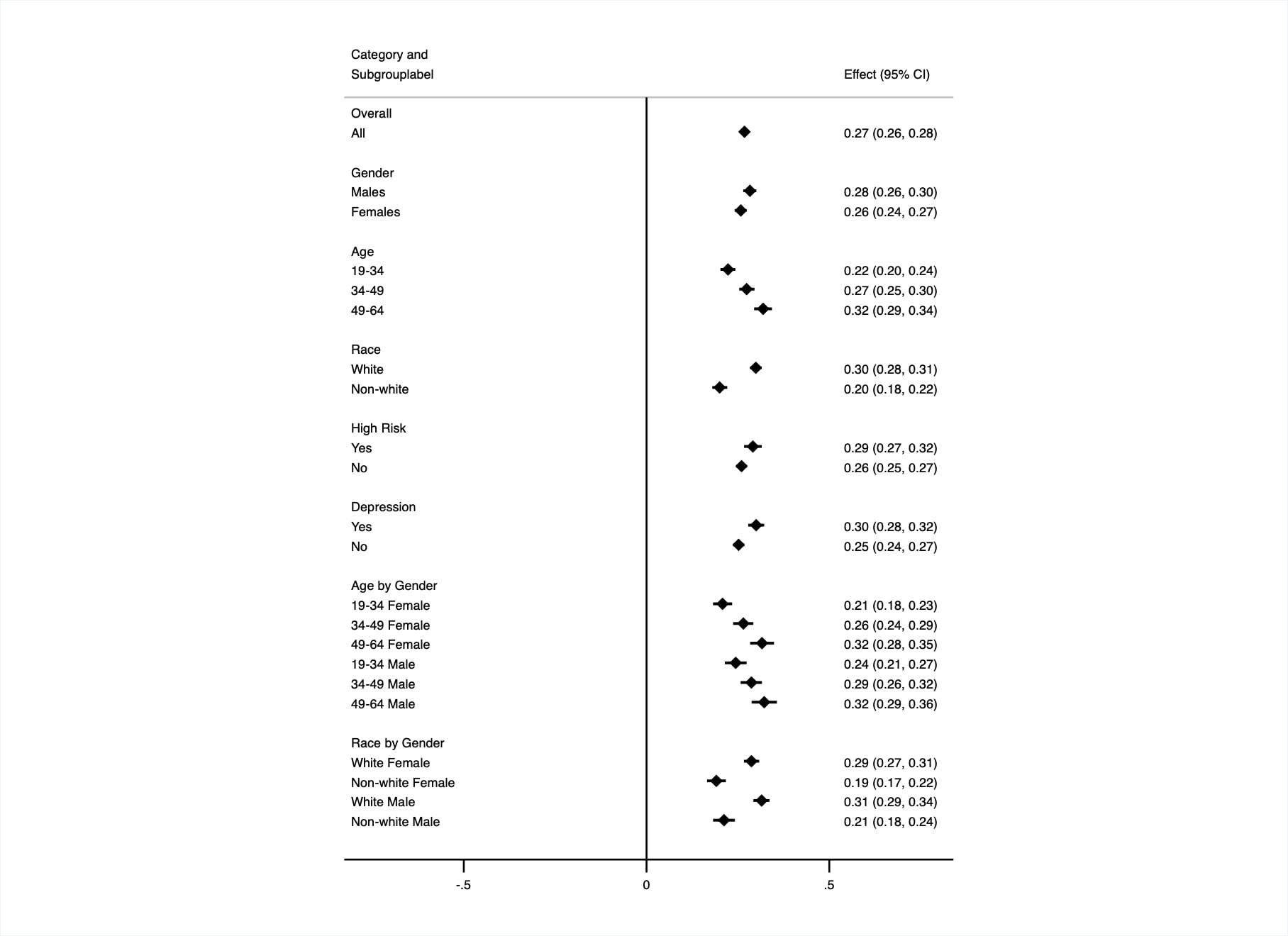

Supplement: S10 Fig — (TIF) [file pone.0297205.s016.tif]

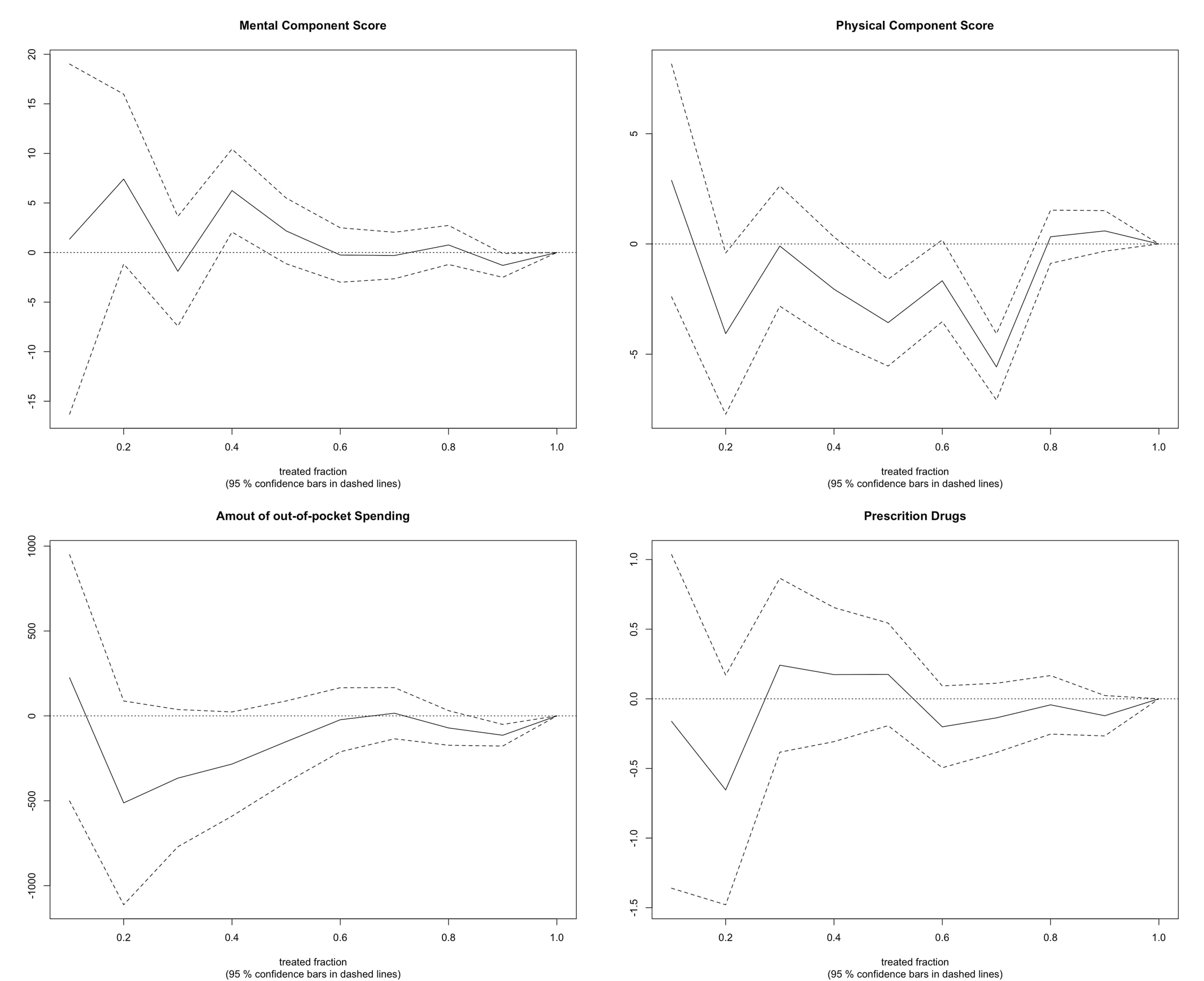

Supplement: S11 Fig — (TIF) [file pone.0297205.s017.tif]

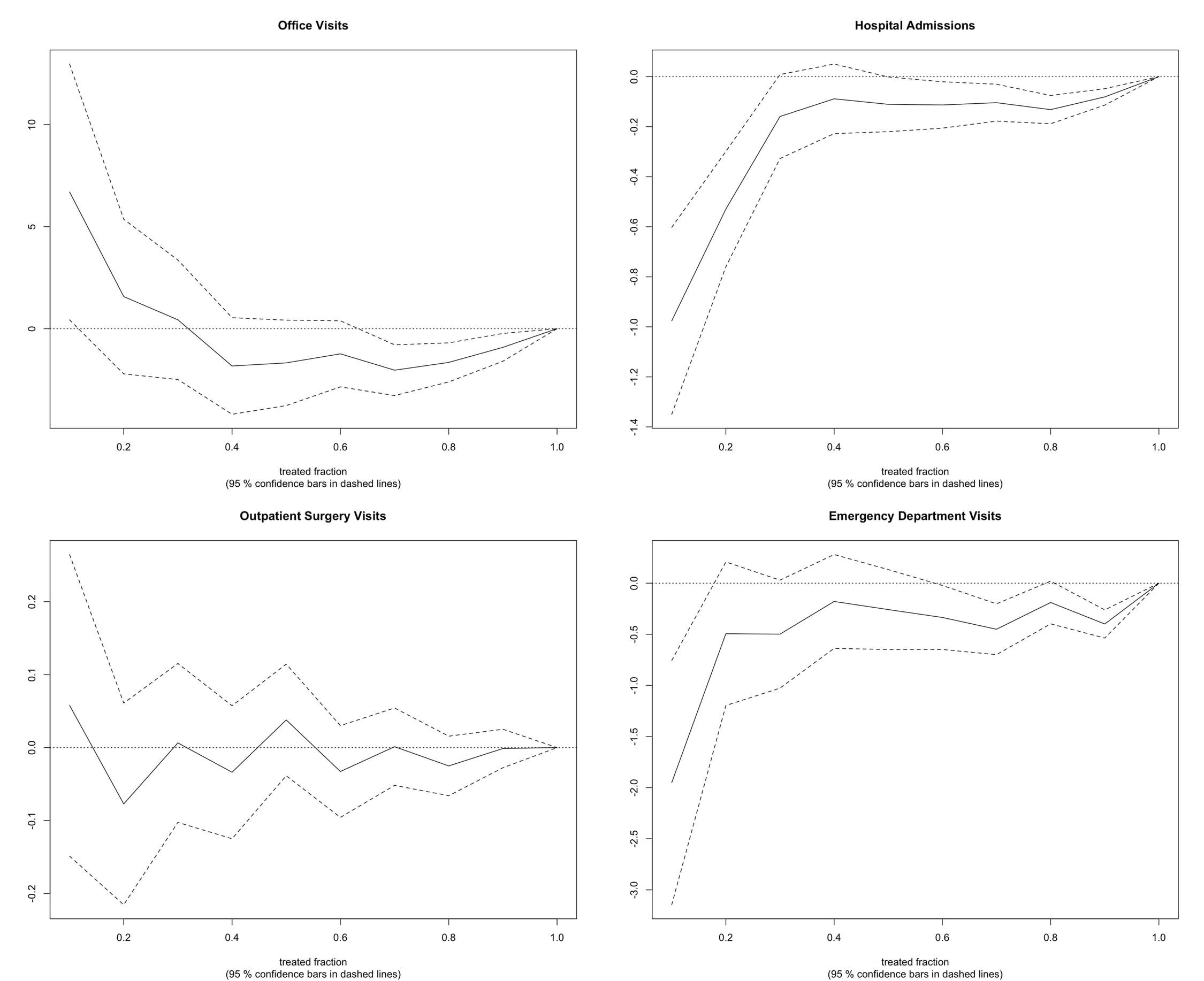

Supplement: S12 Fig — (TIF) [file pone.0297205.s018.tif]

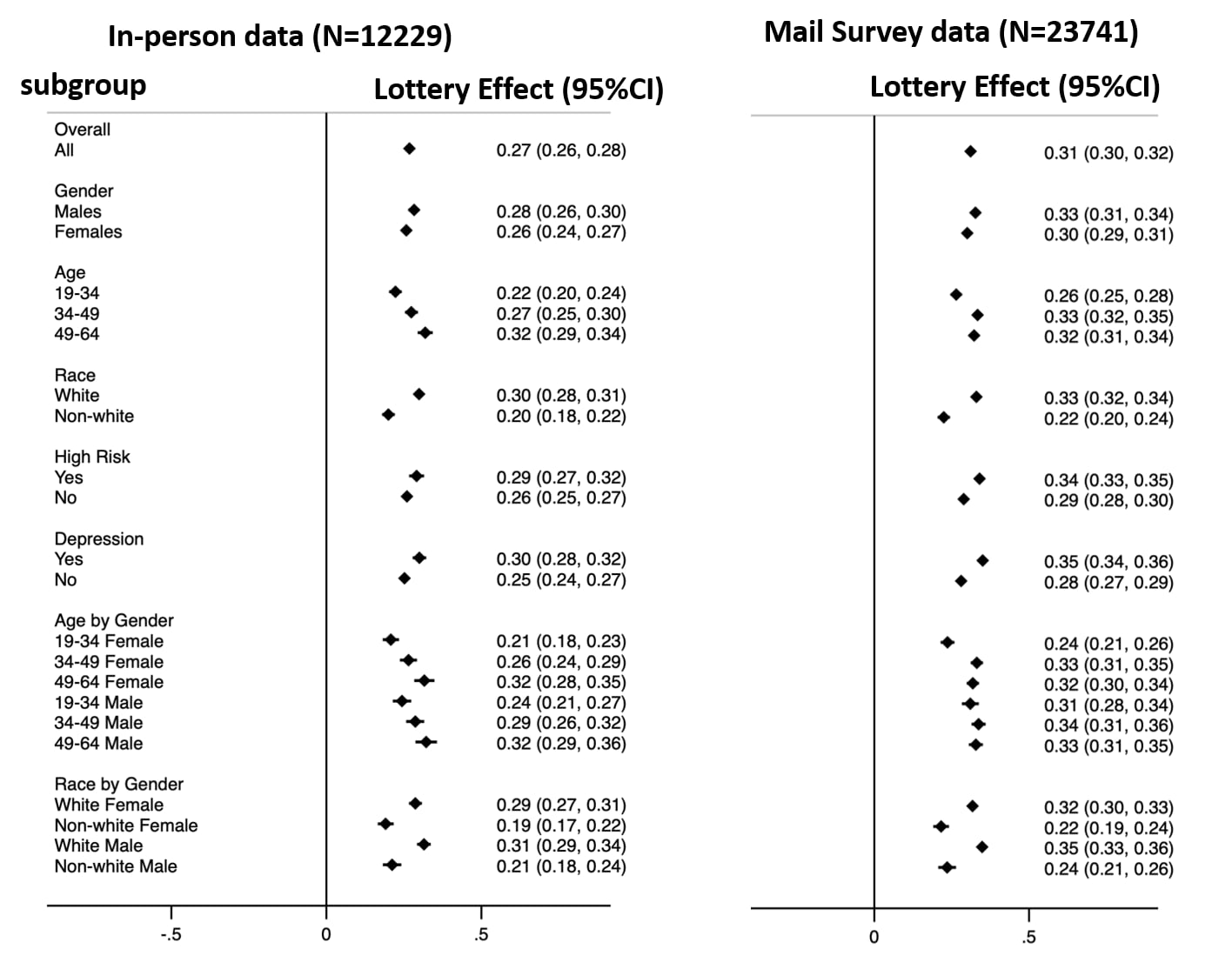

Supplement: S13 Fig — (TIF) [file pone.0297205.s019.tif]

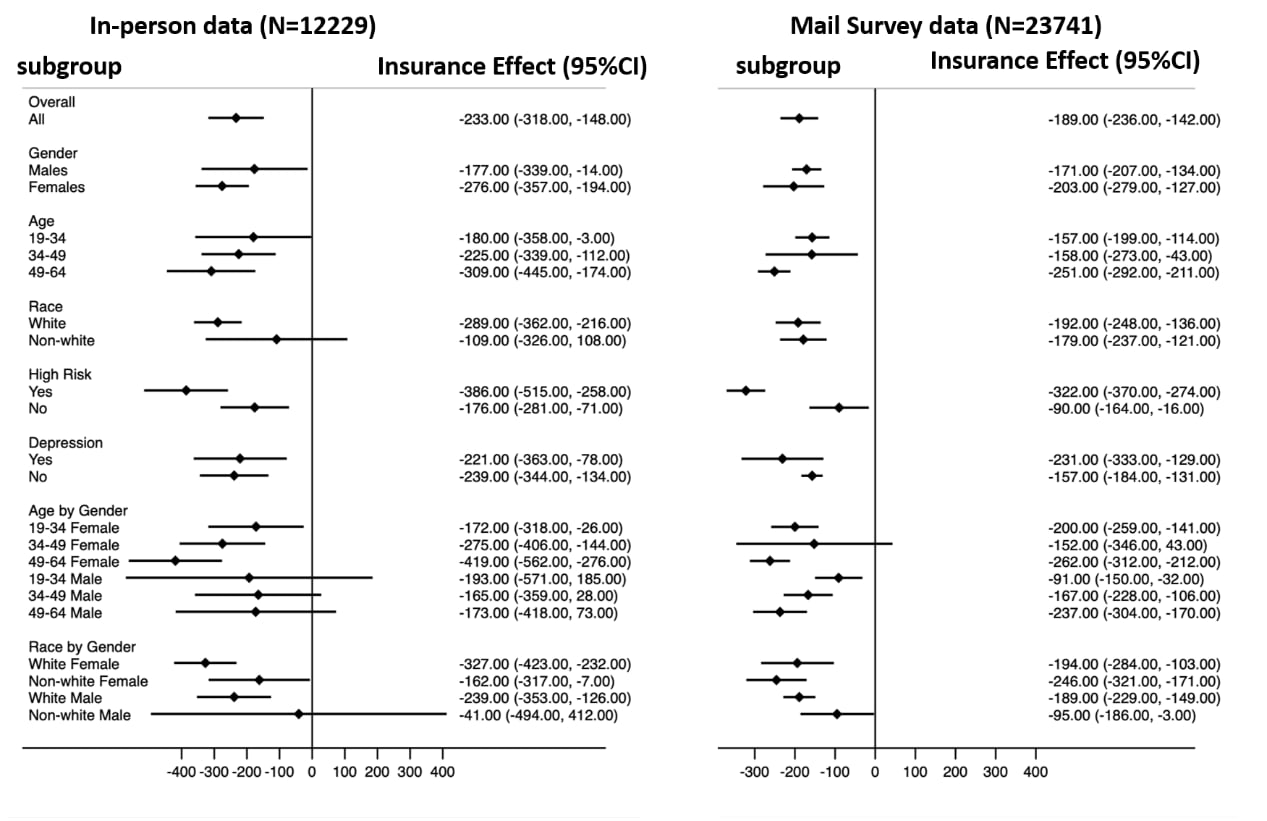

Supplement: S14 Fig — (TIF) [file pone.0297205.s020.tif]

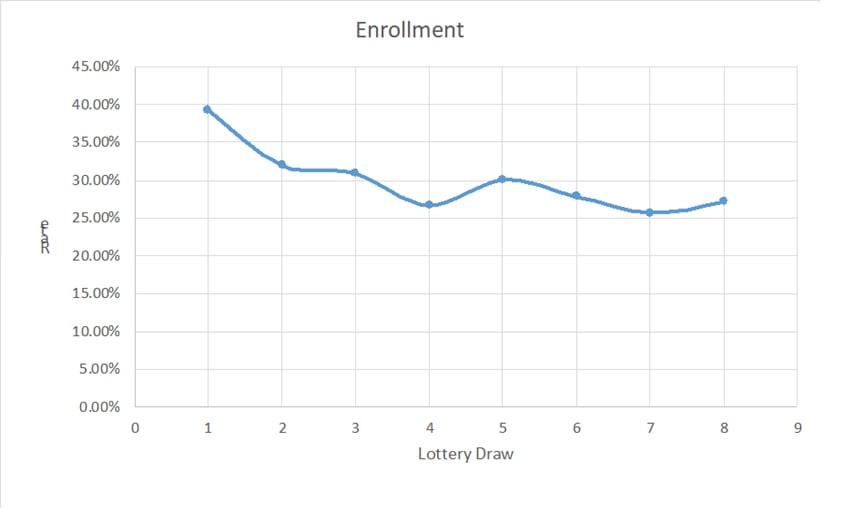

Supplement: S15 Fig — (TIF) [file pone.0297205.s021.tif]

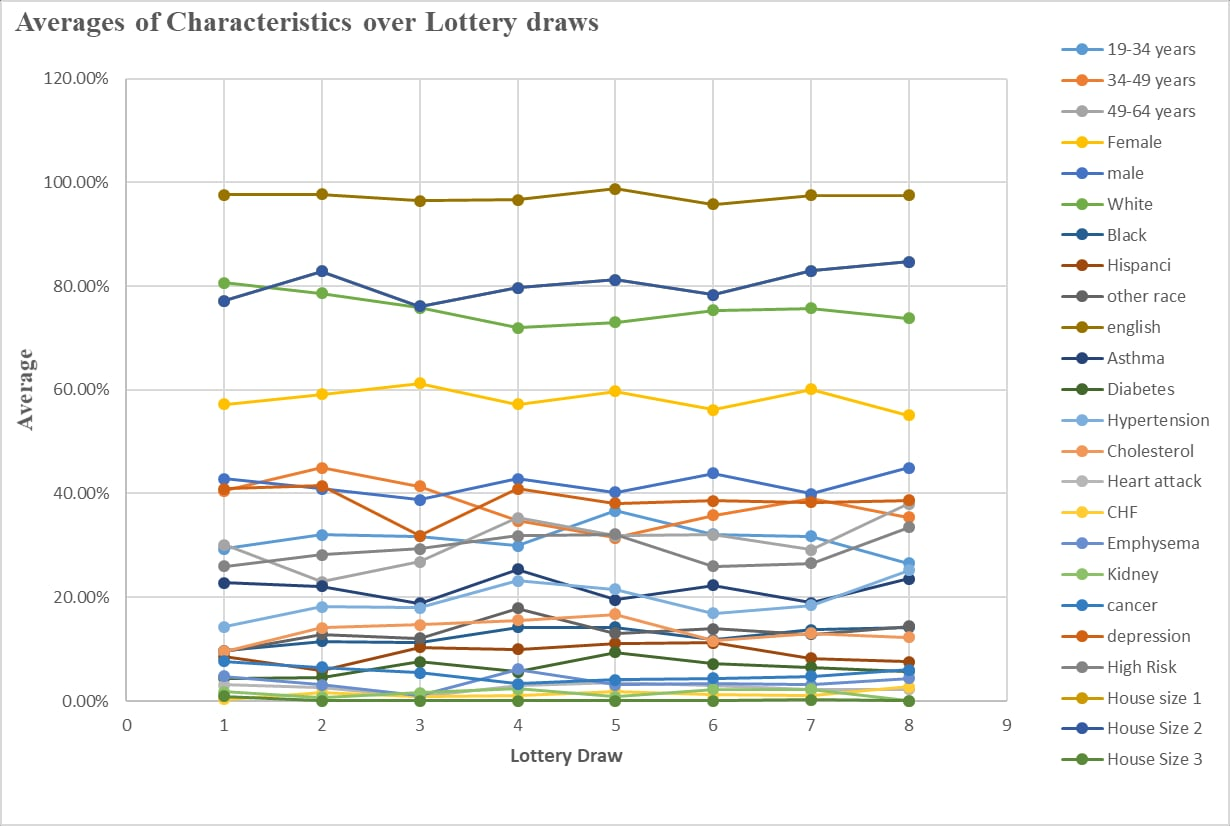

Supplement: S16 Fig — (TIF) [file pone.0297205.s022.tif]

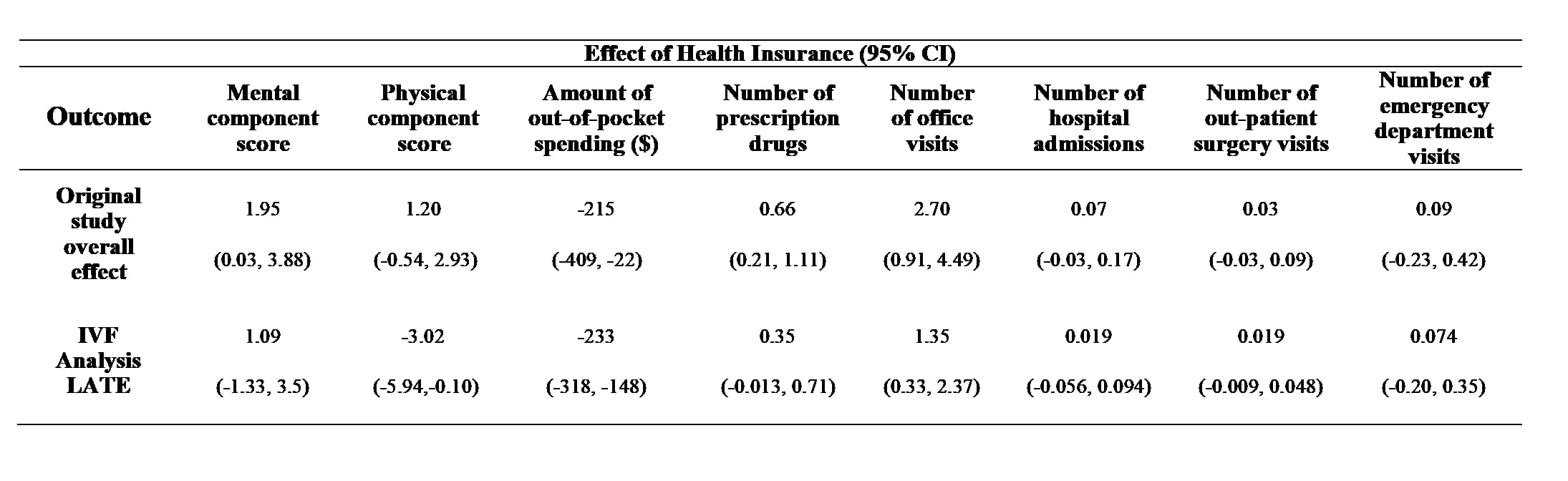

Supplement: S1 Table — (TIF) [file pone.0297205.s023.tif]

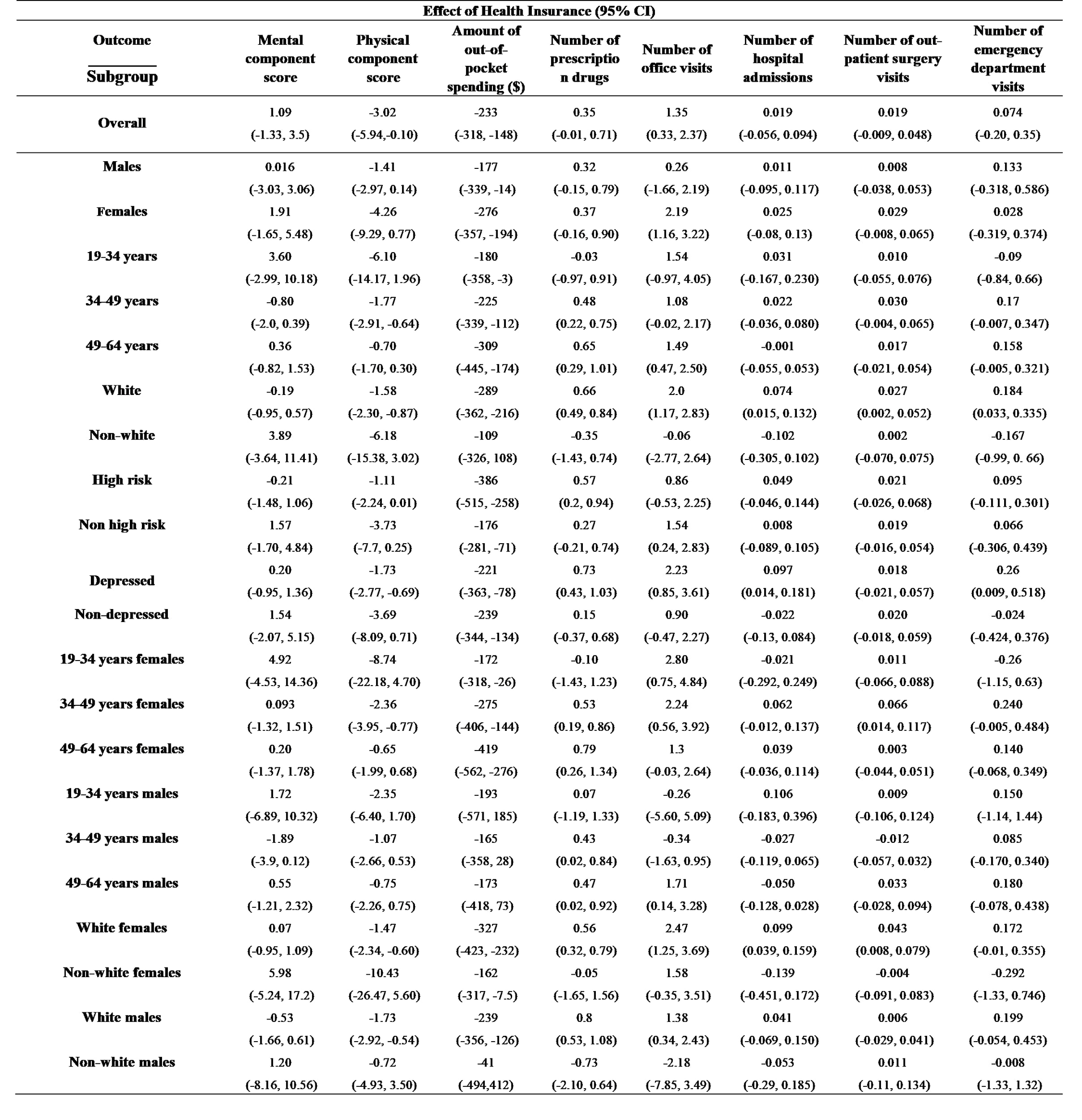

Supplement: S2 Table — (TIF) [file pone.0297205.s024.tif]

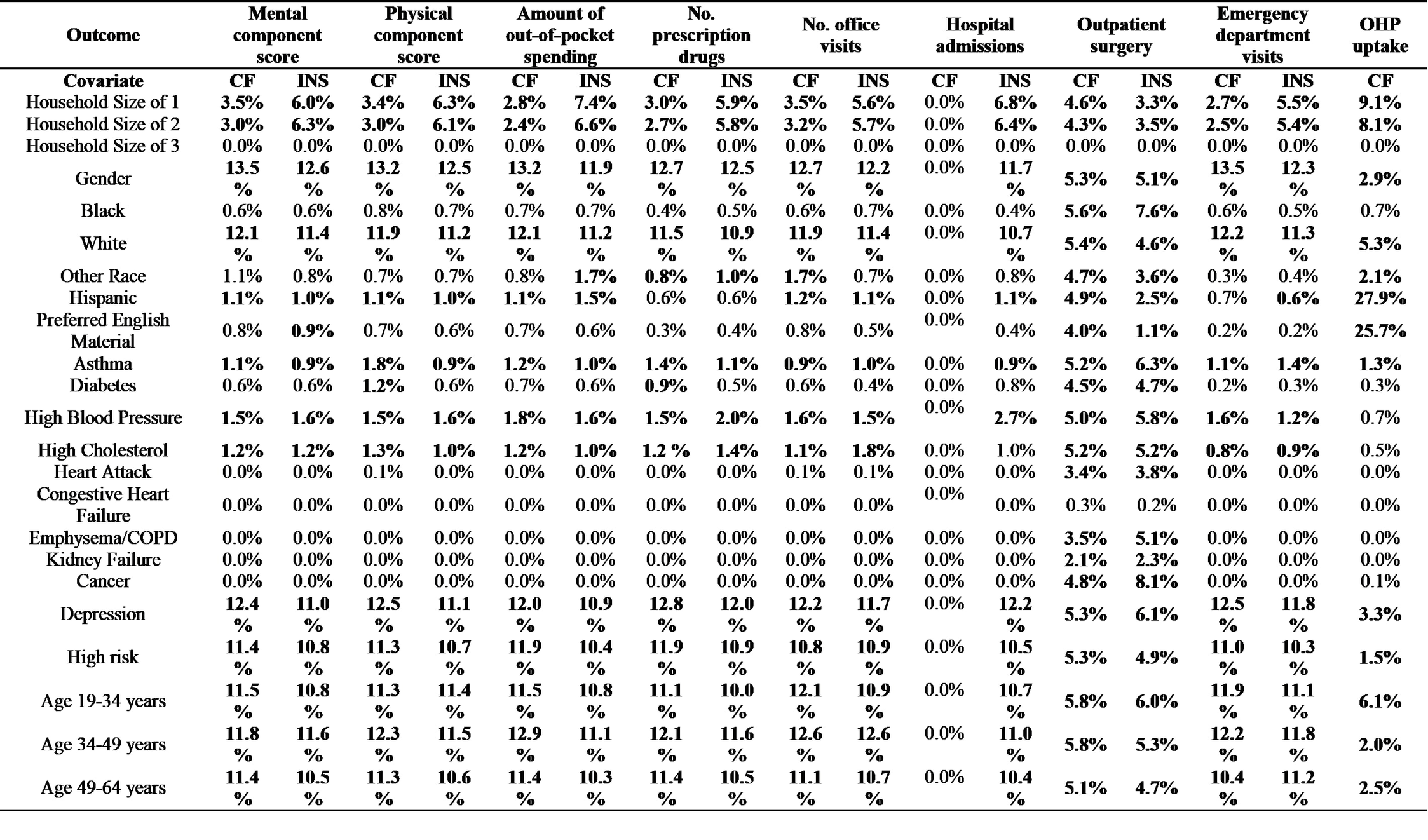

Supplement: S3 Table — (TIF) [file pone.0297205.s025.tif]

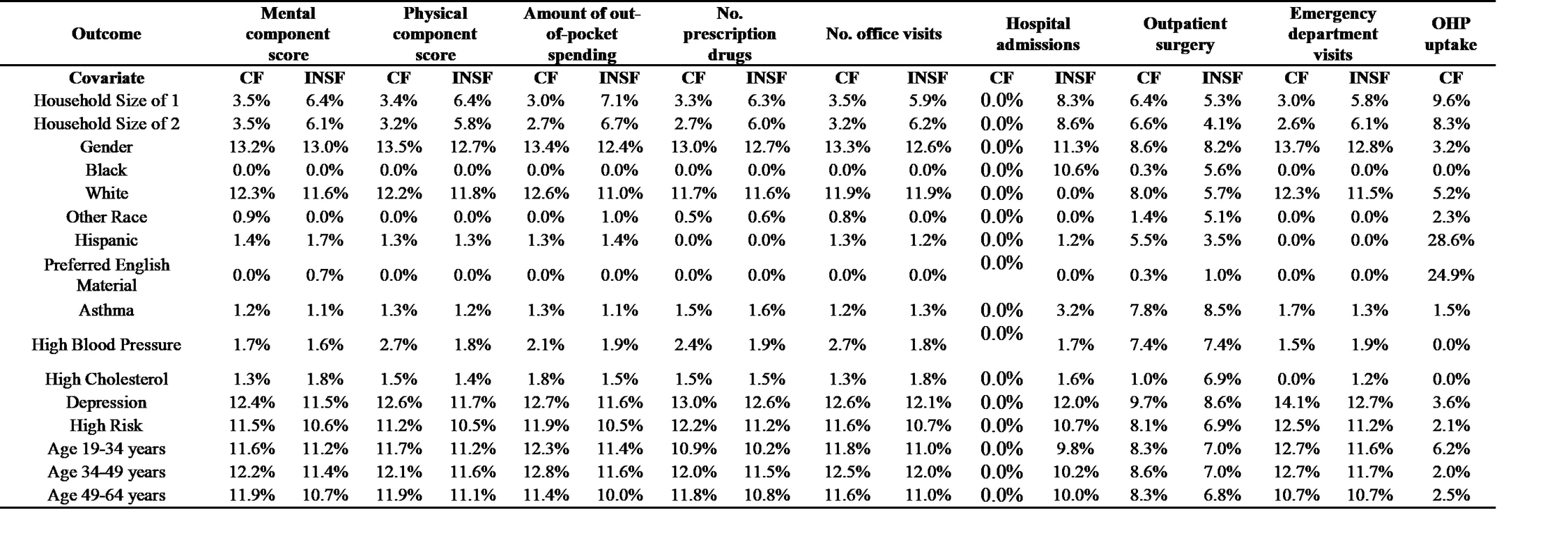

Supplement: S4 Table — (TIF) [file pone.0297205.s026.tif]

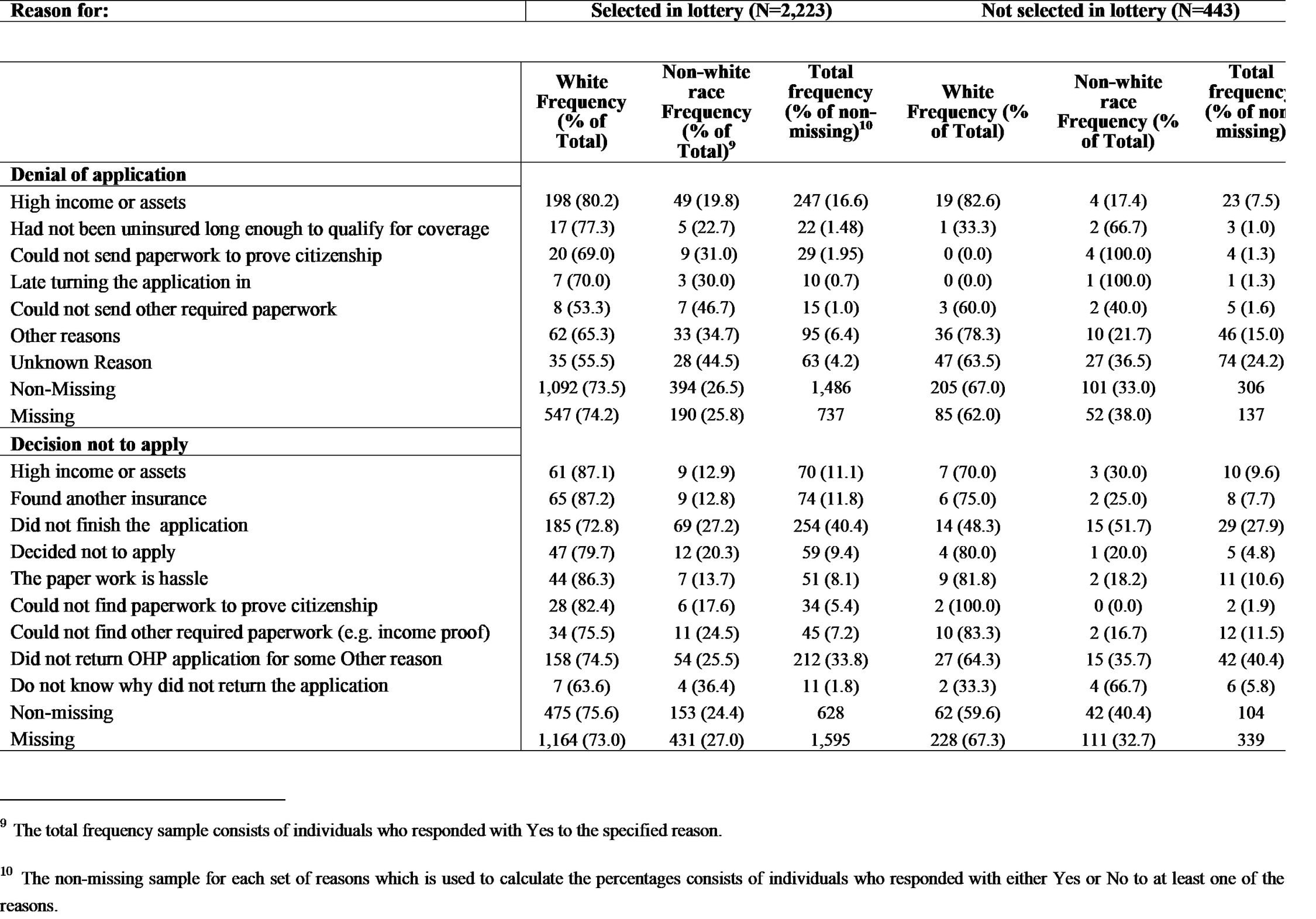

Supplement: S5 Table — (TIF) [file pone.0297205.s027.tif]

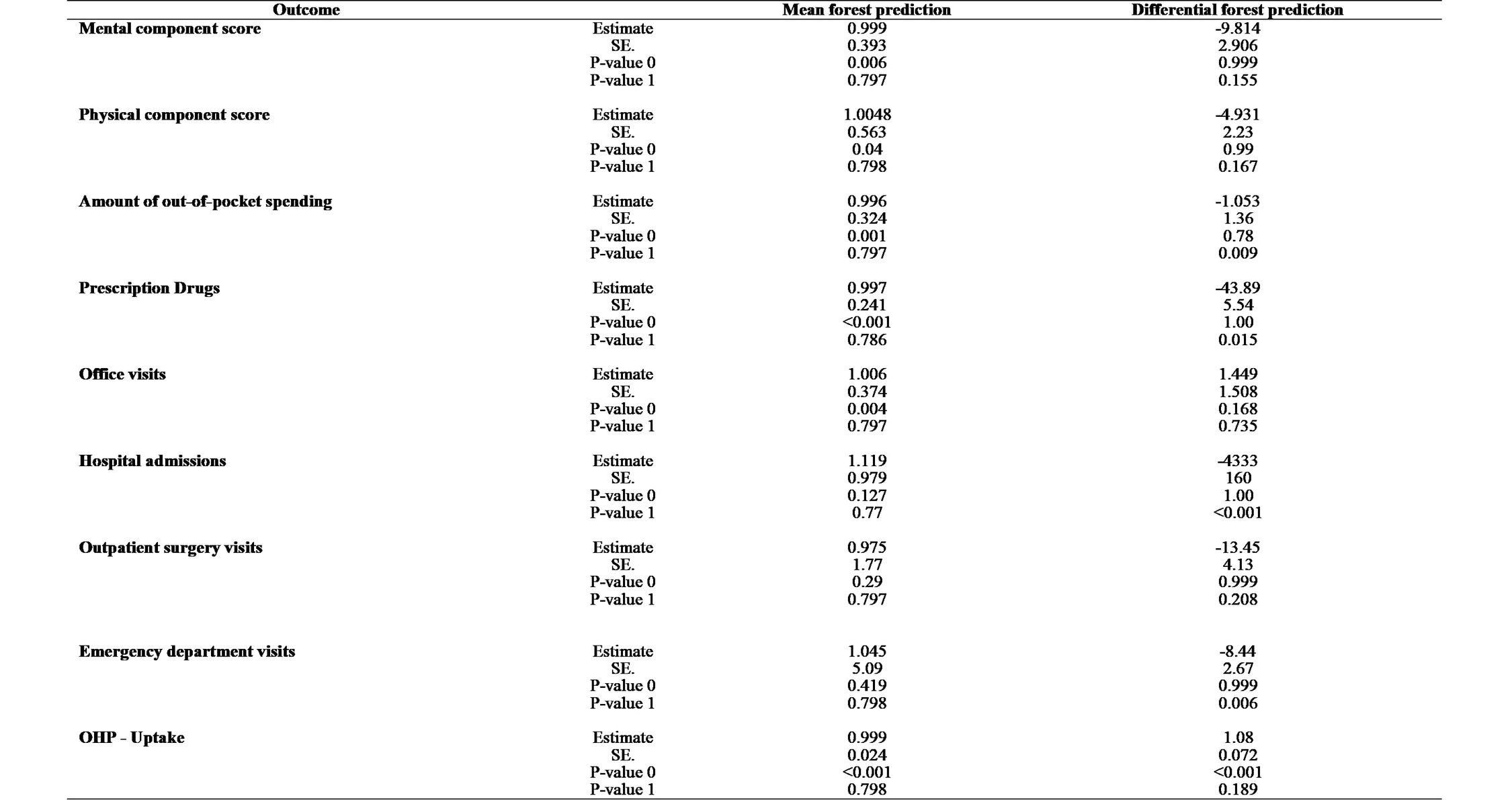

Supplement: S6 Table — (TIF) [file pone.0297205.s028.tif]

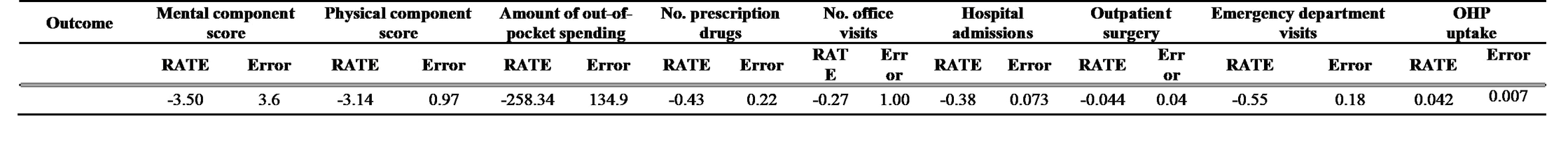

Supplement: S7 Table — (TIF) [file pone.0297205.s029.tif]
